# Impact of the Metal–Ligand Bonding Nature on Electronic States of {Fe(NO)_2_}^9/10^–{Fe(NO)_2_}^9/10^ RREs Unraveled by the Combined X‑ray Spectroscopic Analyses: Relevance to DNIC-Mediated Translational Functionality

**DOI:** 10.1021/acs.inorgchem.6c03317

**Published:** 2026-10-19

**Authors:** Sen-Hung Hsieh, Jia-Yu Lin, Feng-Tian Chang, Chi-Yen Tung, Nozomu Hiraoka, Shu-Chih Haw, Fu-Te Tsai, I-Jui Hsu, Wen-Feng Liaw

**Affiliations:** † Department of Chemistry, 34881 National Tsing Hua University, Hsinchu 300044, Taiwan; ‡ Research and Development Center for Smart Textile Technology, Department of Molecular Science and Engineering, 34877 National Taipei University of Technology, Taipei 106344, Taiwan; § Department of Applied Chemistry, 63107 National Chiayi University, Chiayi 600355, Taiwan; ∥ 57815 National Synchrotron Radiation Research Center (NSRRC), Hsinchu 300092, Taiwan; ⊥ Japan Synchrotron Radiation Research Institute (JASRI/SPring-8), Hyogo 6795198, Japan

## Abstract

Probed by the combined X-ray spectroscopic techniques (N K-edge/Fe L-edge XAS, Fe K-edge HERFD–XANES, Kβ_1,3_ mainline, VtC Kβ_2,5_ XES) and DFT computation, the qualitative/quantitative assignment of Fe/NO oxidation states in dinuclear dinitrosyl iron complexes (DNICs) suggests that the balance between NO^–^ → Fe π-donating and Fe → ^•^NO π-backbonding interactions in the [Fe­(NO)_2_] unit(s) is modulated by the ionicity-covalency competition within the Fe–ER_(2)_ bonds for a series of [ER_(2)_]^−^-bridged {Fe­(NO)_2_}^9/10^–{Fe­(NO)_2_}^9/10^ RREs (E = S, N, O). With the delicately ligand-controlled NO^–^/^•^NO equilibrium in the [Fe­(NO)_2_] unit, the biomimetic activity governed by {Fe­(NO)_2_}^9^/{Fe­(NO)_2_}^10^ DNICs echoes their electronic/spectroscopic features, which not only uncovers molecular identity in complex environments but also decodes the reported H_2_S/NO communication, degradation/repair of [Fe–S] clusters, and translational functionality for NO-to-[N_2_O_2_]^2–^-to-N_2_O and NO-to-HNO/NO^–^ transformations.

## Graphical abstract

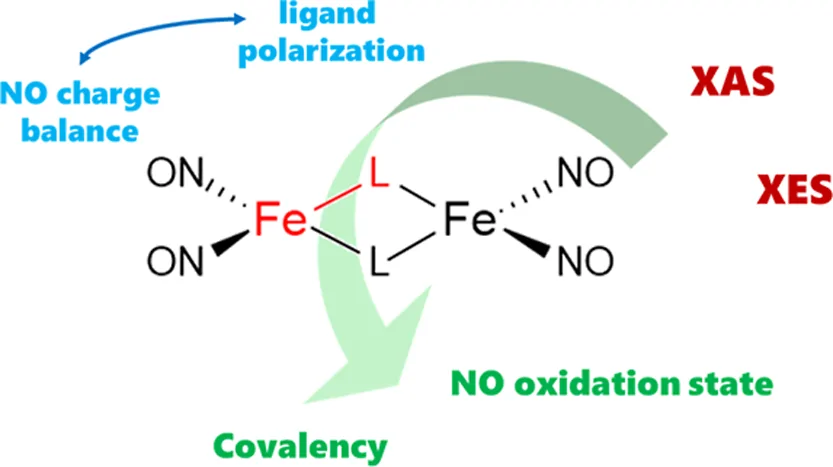

## 1 Introduction

Nitric oxide (NO), a ubiquitous endocrine, is a vital signaling molecule involved in the regulation of the cardiovascular system, cancer biology, cell apoptosis, neurotransmission, redox homeostasis, and immune responses.^1–9^ Under nitrosative stress, dinitrosyl iron complexes (DNICs) as an intrinsic NO-derived species from the degradation of [Fe–S] clusters, iron-containing proteins, and a cellular labile iron pool have been characterized with distinctive electron paramagnetic resonance (EPR) signal at *g* = 2.03.^10–15^ Roussin’s red ester (RRE), the dimeric form of DNICs to exert similar physiological functions as DNICs, is diamagnetic and EPR-silent because of the strong antiferromagnetic coupling between two {Fe­(NO)_2_}^9^ motifs. Interestingly, the dependence of the dynamic equilibrium between EPR-active DNICs and EPR-silent RREs on the pH condition and thiol/imidazole concentration in chemistry may correlate to the question of which species were more prevalent upon nitrosylation of [Fe–S] protein and Rieske [2Fe–2S] cluster in biology.^16–20^ With readily available access to glutathione, glutathione transferases (GSTs) have been discovered as potential DNIC storage and transport proteins. Particularly, the protein-bound DNIC derived from the nitrosylation of human GST P1–1 by a dinitrosyldiglutathionyl iron complex *in vitro*/*in vivo* was identified by X-ray crystallography.^21–23^ For post-translational modification, low-molecular-weight (LMW)–DNICs covalently and reversibly modify cysteine residues of a protein to generate *S*-nitrosothiols (RSNOs) that control active levels of the protein in cell signaling. Besides, LMW–DNICs can transfer a dinitrosyl iron unit [Fe­(NO)_2_] to a protein to form protein-bound DNICs that exert their unique effects on the suppression of glutathione reductase, the inhibition of Na/K ATPase, and the activation of nonselective cation channels.^24–30^

Owing to the noninnocent character of NO and the small energy difference between Fe 3d and NO π* orbitals, the delocalized electron density of the Fe–NO covalent fragment complicates the “physical” assignment of Fe/NO oxidation states in the dinitrosyl iron unit ([Fe­(NO)_2_]) and the illustration of a cooperative relationship among Fe, NO, and S/N/O-containing coordinate ligands.^30–35^ Previously, the combination of Fe K-edge pre-edge energy and S K-edge X-ray absorption spectroscopy (XAS) studies revealed that the oxidation state of Fe is estimated as 2.59 for [(PhS)_2_Fe­(NO)_2_]^−^ when the oxidation states of [Fe^III^(SPh)_4_]^−^ and [Fe^II^(SPh)_4_][Bibr ref2] ^–^ are defined as 3.0 and 2.0, respectively, which lends support to the assignment of NO oxidation state as (−0.77 ± 0.18) using valence-to-core (VtC) Fe X-ray emission spectroscopy (XES).^36,37^ Parallel with the comparable NO stretching frequencies between [(PhS)_2_Fe­(NO)_2_]^−^ (IRν_NO_: 1737 s, 1693 vs cm^–1^ (THF); average IRν_NO_, 1715.0 cm^–1^) and [(PhO)_2_Fe­(NO)_2_]^−^ (IRν_NO_: 1739 s, 1674 vs cm^–1^ (THF); average IRν_NO_, 1706.5 cm^–1^), [(MeO)_2_Fe­(NO)_2_]^**®**^ (IRν_NO_: 1725 s, 1662 vs cm^–1^ (THF); average IRν_NO_, 1693.5 cm^–1^) exhibits the NO stretching frequencies close to those of [(EtS)_2_Fe­(NO)_2_]^−^ (IRν_NO_: 1715 s, 1674 vs cm^–1^ (THF); average IRν_NO_, 1694.5 cm^–1^).^38–42^ Noticeably, the coordination of the hard [OR]^−^ ligands (vs soft [SR]^−^) makes the {Fe­(NO)_2_}^9^ motif display NO stretching frequencies similar to the [SR]^−^-coordinate {Fe­(NO)_2_}^9^ DNICs but induce the electron-deficient Fe core in the {Fe­(NO)_2_}^9^ moiety according to the Fe K-edge pre-edge peak energy ([(PhS)_2_Fe­(NO)_2_]^−^, 7113.5 eV; [(PhO)_2_Fe­(NO)_2_]^−^, 7113.8 eV; [(EtS)_2_Fe­(NO)_2_]^−^, 7113.5 eV; [(MeO)_2_Fe­(NO)_2_]^−^, 7114.2 eV). This paradoxical observation implies that the nature (covalent vs ionic character) of Fe–ER (E = O, S) bonds may play crucial roles in tailoring the iron electronic structure preserved by the noninnocent nature of the NO ligand.^42^

To unravel the relationship between the nature of Fe–ER bonds and the electronic configuration of a dinitrosyl iron unit ([Fe­(NO)_2_]), high-energy-resolution fluorescence-detected (HERFD)–X-ray absorption near edge spectroscopy (XANES) at the Fe K-edge and XAS at the Fe L-edge/N K-edge were employed.^43–51^ Through probing the energetics of the unfilled/partial-filled Frontier orbitals,^43,44^ N K-edge XAS measurement enables the investigation of the changes in the electronic structure of Fe-bound NO ligands induced by the corresponding Fe–ER bonds. The 1s → 3d pre-edge signatures in XANES have been shown to be sensitive to spin state, oxidation state, coordination geometry, and bridge ligation owing to symmetry reflectance of the lowest unoccupied (LUMO) states of iron centers. By means of detecting an emissive final state with a longer lifetime of 3p hole, the Fe K-edge HERFD–XANES technique allows us to theoretically investigate the influence of metal–ligand covalency on LUMO electronic structures in detail.^45–48^ In the Fe L-edge region, the acceptor orbital of a dimeric DNIC could be described as a linear combination of Fe 3d orbitals and ligand np orbitals, of which the normalized wave function is written as ψ* = (1 – α^2^)^1/2^|Fe_3d_⟩ −α|*L*_np_⟩, and α^2^ denotes the ligand character of metal–ligand bonding interaction. Since the L-edge excitation originates from a Fe-centered 2p orbital, the intensity of the electric dipole-allowed 2p → 3d pre-edge transition to the Fe-centered component of the acceptor orbital wave function probes the Fe 3d manifold, and the integrated intensity of the Fe L-edge XAS spectrum is related to the covalency (1−α^2^) of the partially and/or fully unoccupied Fe 3d orbitals. Pioneered by Cramer and Solomon, the total L_2_/L_3_-edge intensity scales proportionally with an Fe 3d character in acceptor orbitals by taking account of atomic and ligand field/charge transfer multiplet effects.^49–51^ Accordingly, combined with single-crystal X-ray diffraction and infrared absorption spectroscopy, the physical oxidation state of Fe center in the dinitrosyl iron unit ([Fe­(NO)_2_]) for a series of {Fe­(NO)_2_}^9/10^–{Fe­(NO)_2_}^9/10^ RREs may be clearly assigned via the shift in the energy of the pre-edge peak in the Fe K-edge HERFD-XANES spectrum.

The paramagnetic nature of mononuclear {Fe­(NO)_2_}^9^ DNICs allows for their extensive characterization through EPR measurements, of which both the distinct g value and hyperfine coupling constants (*a*_NO_/*a*_ligand_) provide rich pictures of electronic and geometric structures in a singly occupied molecular orbital.^33,34,52–55^ In contrast, for EPR-silent {Fe­(NO)_2_}^9^-{Fe­(NO)_2_}^9^ and {Fe­(NO)_2_}^10^-{Fe­(NO)_2_}^10^ RREs, a complementary technique to XAS that offers additional insight into electronic/geometric structures of Fe centers in [Fe­(NO)_2_] units is limited. Kβ XES involves the ionization of a Fe 1s electron and then the detection of the photons emitted from the transition of the electrons in the occupied orbitals to the 1s core hole. Owing to the dominant 3p-3d exchange interaction in the 1s^2^3p^5^3d^n^ final state, the Kβ′/Kβ_1,3_ mainline originating from the Fe 3p → 1s transition is sensitive to metal oxidation state and spin state. Recently, Frank Neese and Serena DeBeer confirmed Kβ mainline as an experimental probe of metal–ligand covalency.^45,56,57^ The relationship between the splitting of the Kβ mainline features (Kβ_1,3_–Kβ′) and the metal–ligand covalency deciphers that covalent reduction of the 3p–3d exchange integral caused by the higher covalency of metal–ligand bonds results in the smaller separation between Kβ_1,3_ and Kβ′ energies, as opposed to the larger split by the prevailing metal–ligand ionicity. In Fe K-edge VtC–XES, a 1s electron is excited nonresonantly and the emissive valence to a metal 1s relaxation channel is monitored. Given that the features represent transitions from filled orbitals that are dominantly ligand in nature, the VtC–XES technique definitively probes the occupied metal-3d/ligand-localized hybrid orbitals by detecting relaxation of electrons from ligand-centered 2p/2s valence orbitals to the metal-centered 1s orbital.^45,57–59^ With regard to the σ-/π-bonding nature of end-on NO^–^/^•^NO/NO^+^ ligands, the lower energy split peaks in the Kβ_2_,_5_ region predominantly arise from Fe–NO molecular orbitals with substantial NO σ_2s_* and σ_2p_ characters, whereas its higher energy portion features the contributions from NO π-derived orbitals.^37,58,60^ In addition to the NO oxidation state being estimated based on the difference of the NO σ_2s_* and σ_2p_ VtC energy splitting (Δ*E*_σ2s*−σ2p_), the analysis of the Kβ_2,5_ features provides significant insights into the relationship between the nature of Fe–NO bonding and electronic structure of the highest occupied (HOMO) orbital in a dimeric DNIC.

The utilization of the complementary XAS and XES spectroscopies is critical for assessing the complicated electronic structures of highly covalent [Fe­(NO)_2_] systems, conquering the limitations of analyzing results from either technique in isolation (Scheme 1). In this work, the Fe/NO oxidation states as well as the correlation among Δ*E*_σ2s*−σ2p_, Kβ_1,3_–Kβ′ and the Fe L-edge integrated area are experimentally delineated by Fe K-edge, Kβ_1,3_/Kβ_2,5_ XES, Fe K-edge HERFD–XANES, and Fe L-edge/N K-edge XAS spectra. Of most importance, the employment of the combined X-ray spectroscopic techniques enables us to explore the electronic states of HOMO/LUMO orbitals within a series of [SR]^−^-bridged {Fe­(NO)_2_}^9/10^–{Fe­(NO)_2_}^9/10^ RREs, [NR_2_]^−^-bridged {Fe­(NO)_2_}^9^–{Fe­(NO)_2_}^9^ RREs and [OR]^−^-bridged {Fe­(NO)_2_}^10^–{Fe­(NO)_2_}^10^ RREs shown in Chart 1, which may lay foundations for relating the nature of an Fe–ER_(2)_ (E = N, O, S) bond to physical oxidation states of an Fe–NO moiety and correlating electronic synergism between the Fe–ER_(2)_ bond and [Fe­(NO)_2_] unit to structure-driven translational functionality.

**Scheme 1:**
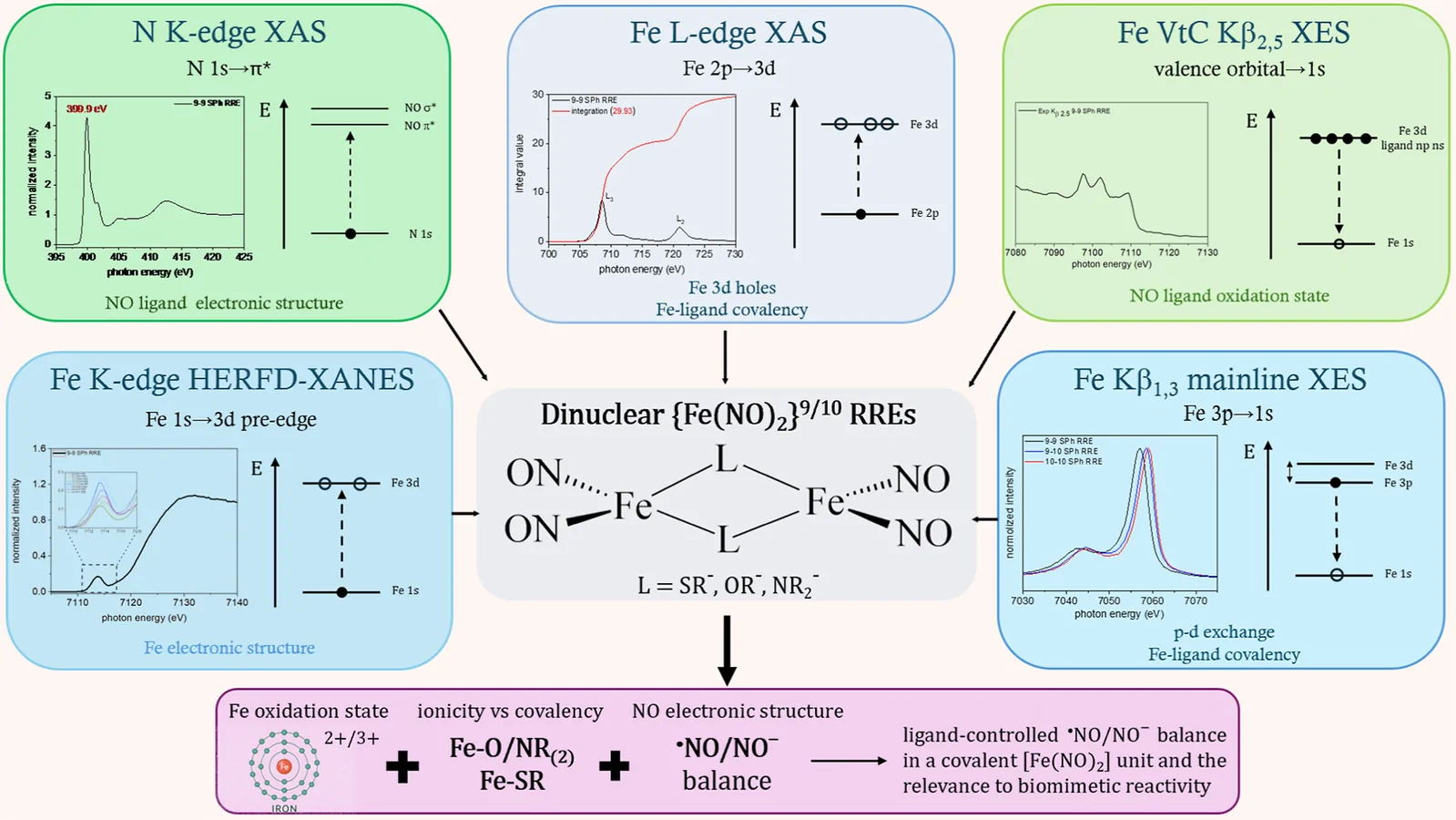


**Chart 1:**
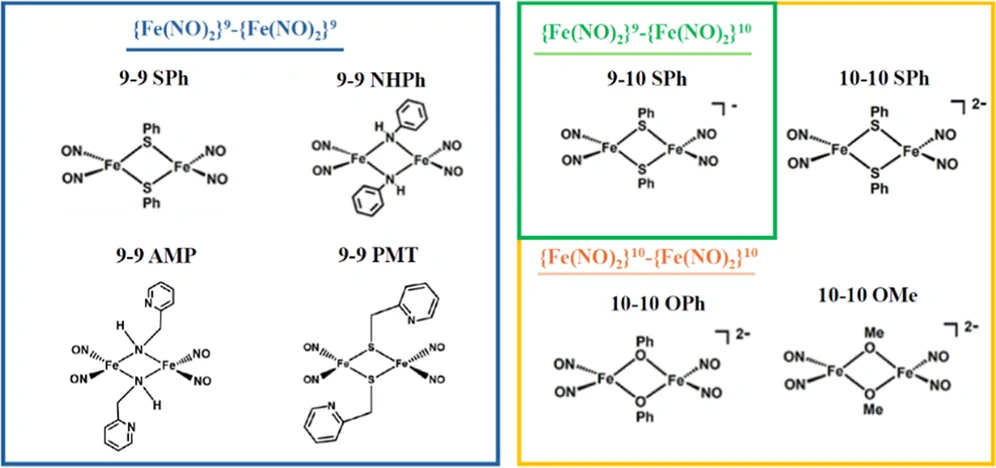


## 2 Results and Discussion

### 2.1 N K-Edge Absorption Spectroscopy

Since the pre-edge feature of N K-edge spectra probes the unfilled/partial-filled Frontier orbitals composed of Fe 3d and NO σ*/π* orbitals, the apparent peak in the range of 398–400 eV (fwhm, 1.0∼1.1 eV) is attributed to N_1s_ → π* (NO) transition, and the broad peak around 413 eV is ascribed to N_1s_ → σ* (NO) transition (Figures 1 and S1).^43,44^ In comparison with **9–9 SPh RRE** displaying a transition peak at 399.9 eV, the migration of the N_1s_ → π* (NO) transition to the lower energy observed for 1e^–^-reduced **9–10 SPh RRE** (399.6 eV) and 2e^–^-reduced **10–10 SPh RRE** (399.4 eV) suggests the more NO^–^ character in the reduced [SR]^−^-bridge RRE, supported by the increase of N–O bond lengths and the decrease of IRν_NO_ stretching frequencies (Table 1). For the series of {Fe­(NO)_2_}^9^–{Fe­(NO)_2_}^9^ and {Fe­(NO)_2_}^10^–{Fe­(NO)_2_}^10^ RREs, the lower N_1s_ → π* (NO) energy of [NR_2_]^−^-bridge {Fe­(NO)_2_}^9^–{Fe­(NO)_2_}^9^ RREs (399.7 eV for **9–9 NHPh RRE** and **9–9 AMP RRE**) and [OR]^−^-bridge {Fe­(NO)_2_}^10^–{Fe­(NO)_2_}^10^ RREs (399.3 eV for **10–10 OPh RRE**, 399.2 eV for **10–10 OMe RRE**), in contrast to that of [SR]^−^-bridged {Fe­(NO)_2_}^9^–{Fe­(NO)_2_}^9^ RRE (399.9 eV for **9–9 SPh RRE** and **9–9 PMT RRE**) and {Fe­(NO)_2_}^10^–{Fe­(NO)_2_}^10^ RREs (399.4 eV for **10–10 SPh RRE**) indicates that the hard/soft nature of the coordinate ligand has an effect on the electronic structure of Fe-bound NO ligands. As described in Table 1, in comparison with **9–9 SPh RRE** displaying the shortest N–O bond length (1.156(4) Å), the highest N K-edge energy (399.9 eV), and IRν_NO_ vibrations (1786, 1759 cm^–1^), **10–10 OMe RRE** has the lowest energy N K-edge feature at 399.2 eV as well as the longest N–O bond lengths at 1.223(3) Å, and the lowest energy IR stretching frequencies at 1620, 1574 cm^–1^. This correlation among N–O bond lengths, IRν_NO_ stretching frequencies, and N_1s_ → π* (NO) transition energy shows that the decrease in N_1s_ → π* (NO) transition energy occurs along with the increase in N–O bond lengths and the decrease in NO vibrational frequencies. In addition to redox chemistry of dimeric DNICs affecting the NO oxidation state, these results implicate that the partially/fully negative-charged NO ligands induced by the enhanced ionicity of Fe–(N/O)­R_(2)_ bond accommodate the [Fe­(NO)_2_] unit to the polarized Fe center imposed by the coordinated hard ligands.

**Figure 1:**
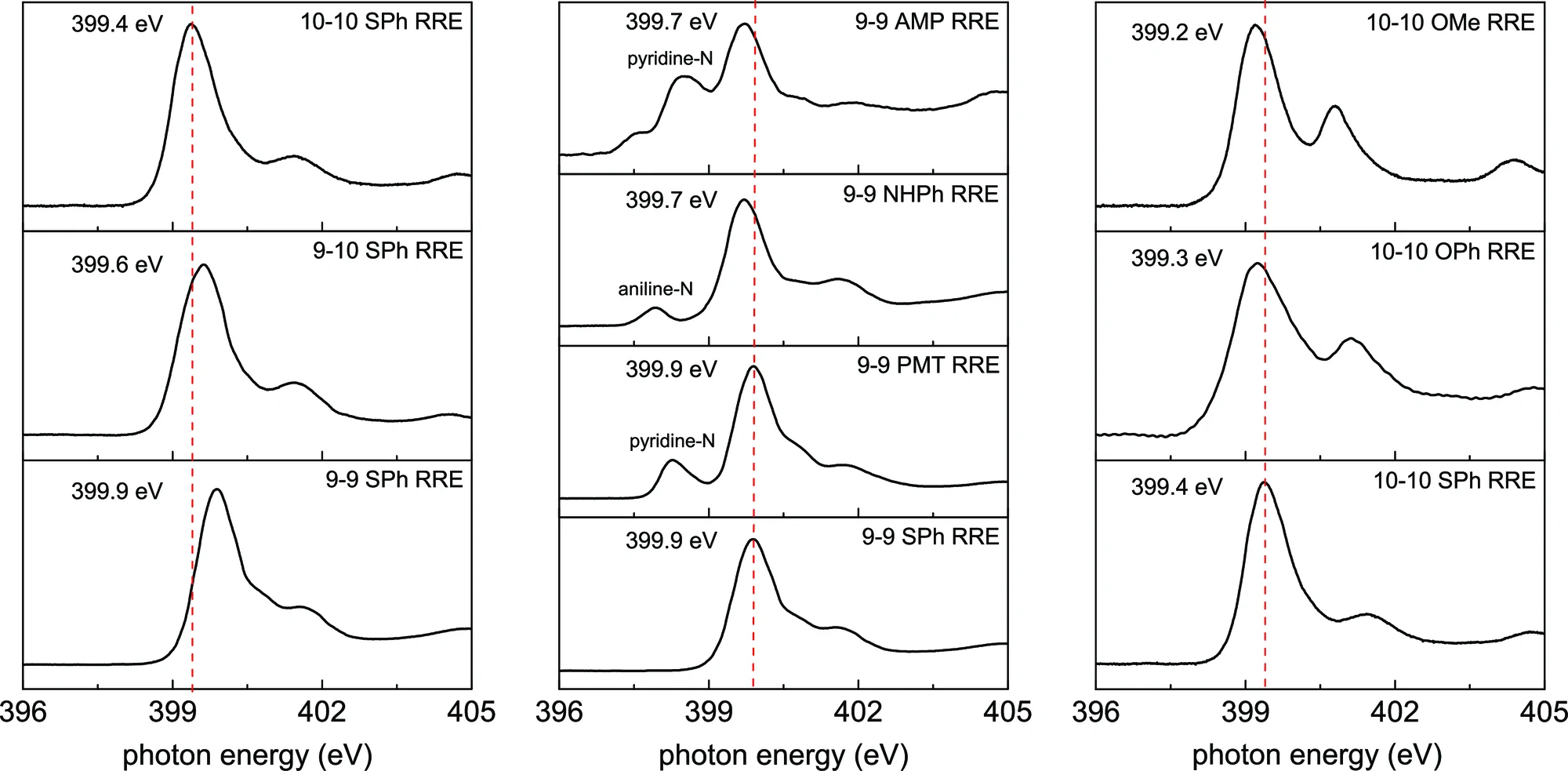
N_1s_ → π* (NO) energies of N K-edge XAS spectra for a series of {Fe­(NO)_2_}^9/10^-{Fe­(NO)_2_}^9/10^ RREs.

**Table 1:** IRν_NO_, Fe/N K-Edge Energies, Fe L-Edge Total Intensity, NO Bond Length, and Experimental Assignment of the Fe Oxidation State for a series of {Fe­(NO)_2_}^9/10^–{Fe­(NO)_2_}^9/10^ RREs

RREs	IRν_NO_ (cm^–1^)^a^ (average)	N–O bond length (Å)^b^	N_1s_ → π* (eV) (fwhm)	Fe 3d holes^c^	Fe L-edge total intensity (per hole)	Fe oxidation state^c^ (pre-edge energy (eV))
**9–9 SPh**	1786, 1759 (1772.5)	1.156 (4)	399.9 (1.0)	9.12	29.93 (3.28)	2.56 (7113.8)
**9–10 SPh**	1680, 1665 (1672.5)	1.184 (5)	399.6 (1.1)	8.44	28.70 (3.40)	2.22 (7113.5)
**10–10 SPh**	1644, 1604 (1624.0)	1.207 (2)	399.4 (1.0)	8.00	28.41 (3.55)	2.00 (7113.3)
**9–9 PMT**	1781, 1751 (1766.0)	1.179 (3)	399.9 (1.0)	9.34	30.91 (3.31)	2.67 (7113.9)
**9–9 NHPh**	1761, 1731 (1746.0)	1.174 (3)	399.7 (1.0)	8.88	39.04 (4.39)	2.44 (7113.7)
**9–9 AMP**	1753, 1725 (1739.0)	1.183 (6)	399.7 (1.1)	8.88	39.40 (4.44)	2.44 (7113.7)
**10–10 OPh**	1657, 1602 (1629.5)	1.211 (2)	399.3 (1.1)	8.22	51.57 (6.27)	2.11 (7113.4)
**10–10 OMe**	1620, 1574 (1597.0)	1.223 (3)	399.2 (1.0)	8.22	54.13 (6.59)	2.11 (7113.4)

a KBr pellets.

b Average N–O bond length by single-crystal X-ray diffraction at 200 K.

c Assuming that the formal Fe 3d holes of the dimeric iron complexes are 8, 9, and 10 for [Fe^II^–Fe^II^], [Fe^II^–Fe^III^], and [Fe^III^–Fe^III^] cores adapting tetrahedral geometry, respectively, a calibration line, Fe 3d holes = 2­(Fe oxidation state) + 4, is applied to calculate Fe L-edge total intensity per hole based on Fe K-edge pre-edge analyses of HERFD–XANES spectra.

### 2.2 Fe L-Edge Absorption Spectroscopy

For the sake of interrogating Fe 3d character contributions to the acceptor orbitals in the ground-state electronic configurations, L_2_/L_3_-edge XAS of formal Fe^II^/Fe^III^ O_h_/T_d_ complexes have been thoroughly interpreted using the valence-bond configuration interaction model invoking atomic and ligand field/charge transfer multiplets.^49–51^ Considering that the N–O bond lengths of {Fe­(NO)_2_}^9/10^–{Fe­(NO)_2_}^9/10^ RREs (Table 1) are in between NO^–^ and ^•^NO (^•^NO, 1.15 Å; NO^–^, 1.26 Å) and the Fe–N–O bond angle ranges from 163.8° to 172.5°,^33,34,70^ it is assumed that the Fe–NO covalency does not change much between the different complexes. With regard to the covalent character of [Fe­(NO)_2_] unit and the difficulty in losing NO under vacuum, we qualitatively describe the nature of Fe–ER_(2)_ (E = O, N, S) bonds through the total intensity of Fe L-edge XAS spectra. Figure S2 and Table 1 show the normalized L-edge absorption spectra of the surveyed dimeric DNICs and the corresponding integrated values. It needs to be noted that, to obtain a covalency trend from the experimental peak area, the number of Fe 3d holes in acceptor orbitals should be taken into account. Because the total intensity at the Fe L-edge reflects the total Fe 3d character in the unoccupied orbitals, the smaller integrated L-edge intensities (per hole) of **9–9 SPh RRE** (29.93 units, 3.28 units per hole) and **9–9 PMT RRE** (30.91 units, 3.31 units per hole) are indicative of the higher covalency of Fe–SR bonds, as compared with **9–10 SPh RRE** (28.70 units, 3.40 units per hole) and **10–10 SPh RRE** (28.41 units, 3.55 units per hole). More importantly, in contrast to the smaller integrated values shown by [SR]^−^-bridge {Fe­(NO)_2_}^9^–{Fe­(NO)_2_}^9^ and {Fe­(NO)_2_}^10^–{Fe­(NO)_2_}^10^ RREs (Table 1), the higher ionic character of Fe–(N/O)­R_(2)_ bonds is disclosed by the larger integrated L-edge intensity of [NR_2_]^−^-bridge {Fe­(NO)_2_}^9^–{Fe­(NO)_2_}^9^ RREs (**9–9 NHPh RRE**, 39.04 units, 4.39 units per hole; **9–9 AMP RRE**, 39.40 units, 4.44 units per hole) and [OR]^−^-bridge {Fe­(NO)_2_}^10^–{Fe­(NO)_2_}^10^ RREs (**10–10 OPh RRE**, 51.57 units, 6.27 units per hole; **10–10 OMe RRE**, 54.13 units, 6.59 units per hole). The combination of Fe L-edge XAS, N K-edge XAS, IRν_NO_, and N–O bond lengths analyses reveal that the population of NO^–^ character of [Fe­(NO)_2_] unit is enhanced by the increased ionic character of Fe-(N/O)­R_(2)_ bonds.

The Fe-3d-character (per hole) trend unveiled by the integrated Fe L-edge intensity (per hole) could be comprehended by hard and soft acids and base (HSAB) theory (Table 1). In accordance with HSABs concept, much of the hard–soft distinction hinges on polarizability so that more electrons and larger size lead to softer behavior. Definitely, [OR]^−^ and [SR]^−^ ligands are classified as hard base and soft base, respectively, while the [NR_2_]^−^ ligand is in the group of hard or borderline base.^61^ Given that the covalent character increases when a ligand is highly polarizable (Fajans’ rule),^62^ the general order of bonding covalency is M–SR > M–NR_2_ > M–OR as these ligands coordinated to the same [Fe­(NO)_2_] fragments. This order stems from orbital differences: [SR]^−^ features a larger ionic radius with diffuse 3p valence electrons, facilitating better orbital overlap with Fe 3d-orbitals. Conversely, [OR]^−^ and [NR_2_]^−^ localize electron density within compact 2p orbitals, restricting covalent sharing. Together, the more the polarizability in the np orbital of [ER_(2)_]^−^ ligand, the higher the covalency of the Fe–ER_(2)_ bond, the less Fe 3d character (per hole) in the acceptor orbital, and the smaller the integrated Fe L-edge intensity (per hole). To further clarify the correlation between Fe–ER_(2)_ covalency and the integrated Fe L-edge intensity, the density functional theory (DFT)-computed 3d-orbital populations at Fe1 and Fe2 of every RRE are listed in Table 2. The 3d-orbital populations could be divided into three groups with ∼7.0*e*, ∼7.1*e*, and ∼7.3*e* based on Löwdin populations (or ∼6.4*e*, ∼6.5*e*, and ∼6.6*e* based on Mulliken populations), corresponding to the RREs with [OR]^−^, [NR_2_]^−^, and [SR]^−^ bridge ligands, respectively. Remarkably, the observed 3d-orbital population trend follows entirely with HSAB framework, implying that the integrated intensity (per hole) of Fe L-edge absorption reflects the polarizability of 2p/3p orbitals in [ER_(2)_]^−^ ligands and thus the covalency of Fe–OR, Fe–NR_2_, and Fe–SR bonds. As shown in Table 1, the average value of Fe L-edge integrated intensity (per hole) for [SR]^−^, [NR_2_]^−^, and [OR]^−^ of ∼29.5 (∼3.4), ∼39.2 (∼4.4), and ∼52.9 (∼6.3) indicates the order of covalent characters as Fe–SR > Fe–NR_2_ > Fe–OR, commensurate with Table 2 where the number of holes in the 3d-orbital population is in the order ∼2.7 < ∼2.9 < ∼3.0. Considering the insight into [SR]^−^-bridged {Fe­(NO)_2_}^9/10^–{Fe­(NO)_2_}^9/10^ RREs (Table 2), the decrease in 3d-orbital populations from 7.328*e*, 7.318*e* to 7.223*e* suggests the decrease in Fe–SR covalency, resulting in more π back-donation (Fe 3d → NO π*) to reduce the Fe–N­(O) distance but increase the N–O distance and negative charge (Table 1). These observations well align with the electronic structure described by Lo et al., wherein the HOMO state of **10–10 SPh RRE** is dominated by Fe (3d)–S (3p) antibonding and Fe (3d)–NO (π*) bonding interactions. Therefore, the subsequent oxidation to **9–10 SPh RRE** and **9–9 SPh RRE** shortens the Fe–S bonds along with an elongation of the Fe–N­(O) bond.^72^

**Table 2:** DFT Computed 3d-Orbital Populations for a Series of {Fe­(NO)_2_}^9/10^–{Fe­(NO)_2_}^9/10^ RREs

	Löwdin			Mulliken		
RREs	Fe1	Fe2	avg	Fe1	Fe2	avg
**9–9 SPh**	7.328	7.328	7.328	6.596	6.596	6.596
**9–10 SPh**	7.315	7.322	7.318	6.575	6.584	6.579
**10–10 SPh**	7.223	7.223	7.223	6.521	6.521	6.521
**9–9 PMT**	7.318	7.318	7.318	6.597	6.597	6.597
**9–9 NHPh**	7.115	7.115	7.115	6.524	6.524	6.524
**9–9 AMP**	7.116	7.116	7.116	6.536	6.536	6.536
**10–10 OPh**	7.030	7.030	7.030	6.458	6.458	6.458
**10–10 OMe**	6.994	6.994	6.994	6.422	6.422	6.422

### 2.3 Fe K-Edge HERFD-XANES Analyses

Since the pre-edge feature coincides with Fe 1s excitation into the unresolved unoccupied states comprised of hybrid Fe 3d–ER_(2)_ 2p/3p acceptor orbitals and the orbitals gaining metal hole character through Fe–NO π back-bonding in the dimeric DNIC system, HERFD–XANES measurements were performed to unveil the Fe oxidation state and Fe–ER_(2)_ covalency order by integrating over a narrow range centered on Kβ_1,3_ fluorescence line (Fe 3p → 1s transition) with a fixed radiative decay energy for the acquirement of high-energy-resolution XAS spectra in a partial fluorescence yield. The normalized Fe K-edge HERFD–XANES spectra for the dimeric DNIC complexes are compiled in Figure 2a, and the energies obtained from the first derivative of the pre-edge peaks (dμ/d*E* = 0) are provided in Table 1. All of pre-edge energies fall within the reported range of {Fe­(NO)_2_}^9^ DNICs (7113.5–7114.2 eV) and {Fe­(NO)_2_}^10^ DNICs (7113.0–7113.4 eV).^33,34,70^ The lower pre-edge energy displayed by **9–10 SPh RRE** (7113.5 eV) and **10–10 SPh RRE** (7113.3 eV) implies the effective nuclear charge (*Z*_eff_) of the Fe center decreases upon 1e^–^/2e^–^-electron reduction of **9–9 SPh RRE** (7113.8 eV). Taking the pre-edge position of [NR_2_]^−^-bridge {Fe­(NO)_2_}^9^–{Fe­(NO)_2_}^9^ RREs and [OR]^−^-bridge {Fe­(NO)_2_}^10^–{Fe­(NO)_2_}^10^ RREs for comparison, the harder nature (vs [SR]^−^-bridge **10–10 SPh RRE** (7113.3 eV)) of phenoxide/methoxide ([OR]^−^) ligands realizes the small increase in *Z*_eff_ charge of the Fe center for **10–10 OPh RRE** (7113.4 eV) and **10–10 OMe RRE** (7113.4 eV). The more NO^–^ character induced by the hard [NR_2_]^−^ ligand (Figure 1) and the electron-donating ability (vs soft thiolate ligand, [SR]^−^-bridge **9–9 PMT RRE** (7113.9 eV)) of the amide ligand work together to rationalize the decreasing pre-edge energy of **9–9 NHPh RRE** (7113.7 eV) and **9–9 AMP RRE** (7113.7 eV). When the oxidation states of [Fe^III^(OPh)_4_]^−^ and [(μ-SPh)­Fe^II^(SPh)_2_]_2_^2–^ are defined as 3.0 and 2.0 standards, respectively, the Fe oxidation state of [Fe­(NO)_2_] unit in every RRE could be spectroscopically assigned by interpolating the regression line, pre-edge energy = 0.9 (Fe oxidation state) + 7111.5 (Figure 2b and Table 1). For the covalent [Fe­(NO)_2_] unit, the assignment of Fe oxidation state uncovers the increased covalent order of the metal–ligand bond as [OR]^−^ < [NR_2_]^−^ < [SR]^−^, suggesting that the modulation of the 1s-LUMO gap (1s → 3d pre-edge energy) is achieved through the regulatory contribution of Fe–ER_(2)_ bonding covalency. Echoed with Fe L-edge and N K-edge XAS results, Fe K-edge pre-edge energy unravels the noninnocent nature of NO ligands as an electron buffer to attenuate the electronic deficiency of the Fe center induced/polarized by the incremental ionicity of Fe–(N/O)­R_(2)_ bonds such that the highly covalent [Fe­(NO)_2_] unit could be preserved. In accordance with the antibonding (partially/fully unoccupied) orbitals being characterized by X-ray absorption spectroscopy, the LUMO orbital of [Fe­(NO)_2_] unit in {Fe­(NO)_2_}^10^–{Fe­(NO)_2_}^10^ RREs may be portrayed as an {Fe^II^(NO^–^)_2_}^10^ electronic configuration. On the contrary, the LUMO orbital of the [Fe­(NO)_2_] unit in {Fe­(NO)_2_}^9^–{Fe­(NO)_2_}^9^ RREs may be qualitatively described as a resonance hybrid of {Fe^II^(NO^–^)­(^•^NO)}^9^ and {Fe^III^(NO^–^)_2_}^9^ electronic structures, and the prevalence of the {Fe^III^(NO^–^)_2_}^9^ electronic structure may be ascribed to the dominant ionic contribution of Fe–(N/O)­R_(2)_ bonds.

**Figure 2:**
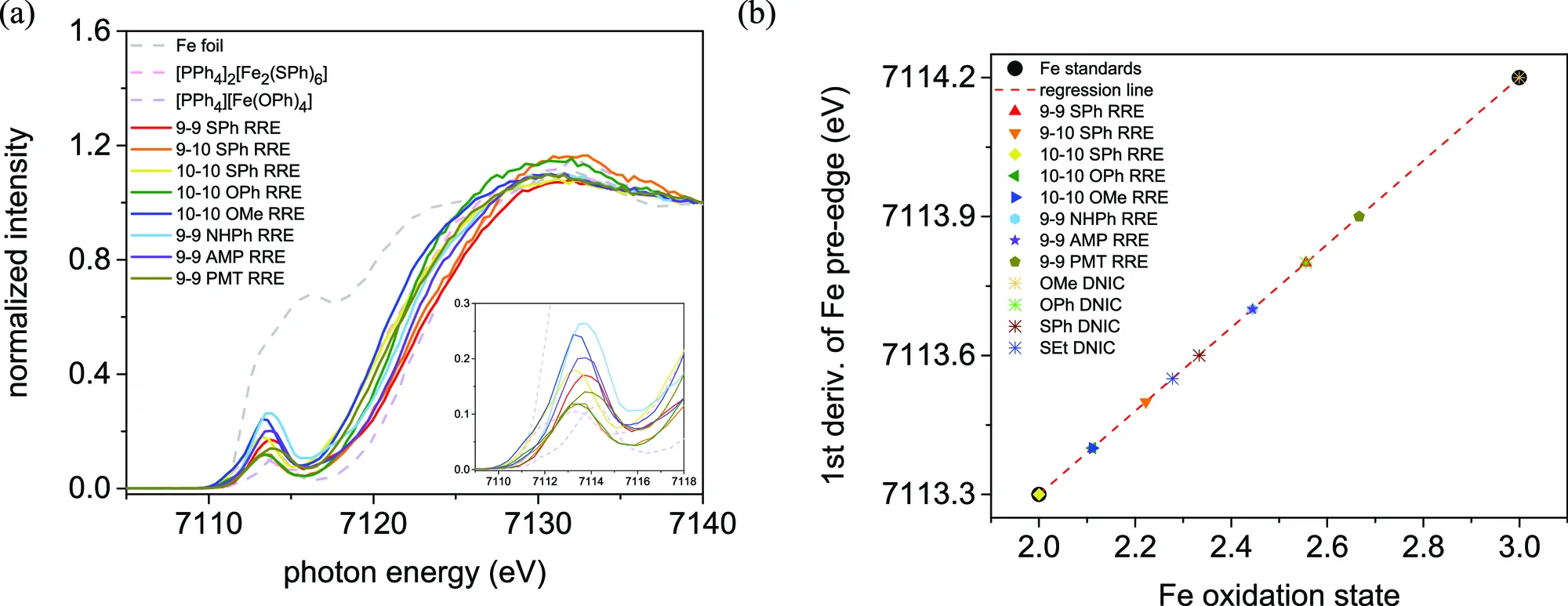
(a) HERFD–XANES spectra for a series of {Fe­(NO)_2_}^9/10^–{Fe­(NO)_2_}^9/10^ RREs. The inset is the enlarged Fe–K-edge pre-edge features. (b) Experimental assignment of Fe oxidation states for a series of {Fe­(NO)_2_}^9/10^–{Fe­(NO)_2_}^9/10^ RREs and {Fe­(NO)_2_}^9^ DNICs by interpolation. With [(μ-SPh)­Fe­(SPh)_2_]_2_^2–^ and [Fe­(OPh)_4_]^−^ as the standards for Fe^II^ and Fe^III^ oxidation states, the linear regression line follows the equation: pre-edge energy = 0.9 (Fe oxidation state) + 7111.5 with *R* ^2^ = 1.0.

### 2.4 Kβ Mainline and VtC Emission Spectroscopy

Regarding that the Kβ_1,3_–Kβ′ splitting energy governed by the final-state exchange coupling between unpaired 3p holes and unpaired 3d electrons is modulated by the metal–ligand covalency,^45,56^ the nominal Fe 3d spin information and Fe–ER_(2)_ bond covalent character for a series of {Fe­(NO)_2_}^9/10^–{Fe­(NO)_2_}^9/10^ RREs were examined by Kβ_1,3_ XES spectra (Figure S3). The high-spin configuration of tetrahedral geometry adapted by the Fe center gives rise to relatively intense Kβ′ features and the visible energy separation between Kβ_1,3_ and Kβ′ peaks. Considering that the cancellation may occur between the changes in oxidation state and in metal–ligand covalency, the effect of the Fe–ER_(2)_ bonding character on Kβ_1,3_–Kβ′ splitting energy is discussed separately based on the same bridge ligand or/and oxidation state. For [SR]^−^-bridge RREs, the increase in Kβ_1,3_–Kβ′ energy separation resulting from 1e^–^/2e^–^ reduction of {Fe­(NO)_2_}^9^–{Fe­(NO)_2_}^9^ RRE (**9–9 SPh RRE**, 14.31 eV; **9–10 SPh RRE**, 14.44 eV; **10–10 SPh RRE**, 14.87 eV) is indicative of the decreasing Fe–SR bond covalency. In parallel with the larger split being induced by the coordination of the harder [OMe]^−^ ligand (vs [OPh]^−^ and [SPh]^−^) in {Fe­(NO)_2_}^10^–{Fe­(NO)_2_}^10^ RREs (**10–10 SPh RRE**, 14.87 eV; **10–10 OPh RRE**, 15.27 eV; **10–10 OMe RRE**, 15.43 eV), the incremental ionicity of Fe–NR_2_ bonds (vs Fe–SR bonds) in {Fe­(NO)_2_}^9^–{Fe­(NO)_2_}^9^ RREs leads to the increase in the energy difference between Kβ_1,3_ and Kβ′ peaks (**9–9 PMT RRE**, 14.32 eV; **9–9 NHPh RRE**, 14.71 eV; **9–9 AMP RRE**, 14.73 eV). With respect to bonding covalency as the operative factor of the decreasing mainline splits,^56^ the largest splitting (15.43 eV) between Kβ_1,3_ and Kβ′ signals observed in **10–10 OMe RRE**, as opposed to the smallest splitting (14.31 eV) shown by **9–9 SPh RRE**, explicates that Fe–ER_(2)_ bonding covalency decreases when electron-rich Fe center ({Fe­(NO)_2_}^10^ motif) is bound to the harder [OR]^−^/[NR_2_]^−^ligands (Table 3), lending support to the correlation between Fe L-edge peak area per hole and Kβ_1,3_–Kβ′ energy split (Figure 3a).

**Table 3:** Kβ_1,3_ Energy, Kβ′ Energy, Kβ_1,3_–Kβ′ Splitting Energy, VtC Kβ_2,5_ XES NO σ_2s_* and NO σ_2p_ Energy Splitting (Δ*E*_σ2s*−σ2p_) and Experimental Assignment of the NO Oxidation State for a Series of {Fe­(NO)_2_}^9/10^–{Fe­(NO)_2_}^9/10^ RREs

RREs	Kβ_1,3_ energy (eV)	Kβ′ energy (eV)	Kβ_1,3_–Kβ′ (eV)	VtC Kβ_2,5_ XES Δ*E*_σ2s*−σ2p_ (eV)	NO oxidation state^a^
**9–9 SPh**	7056.99	7042.68	14.31	5.02	+0.11
**9–10 SPh**	7058.47	7044.03	14.44	5.15	–0.13
**10–10 SPh**	7058.98	7044.11	14.87	5.46	–0.69
**9–9 PMT**	7059.46	7045.24	14.32	5.07	+0.02
**9–9 NHPh**	7059.06	7044.35	14.71	5.22	–0.26
**9–9 AMP**	7058.79	7044.06	14.73	5.12	–0.08
**10–10 OPh**	7058.75	7043.48	15.27	5.56	–0.87
**10–10 OMe**	7059.11	7043.68	15.43	5.62	–0.98

a The linear correlation of oxidation state of NO and the energy difference between σ_2s_* and σ_2p_ orbitals of NO is described as follows, Δ*E*_σ2s*−σ2p_ = −0.550 (NO oxidation state) + 5.079 with a standard error of 0.11.^37^

**Figure 3:**
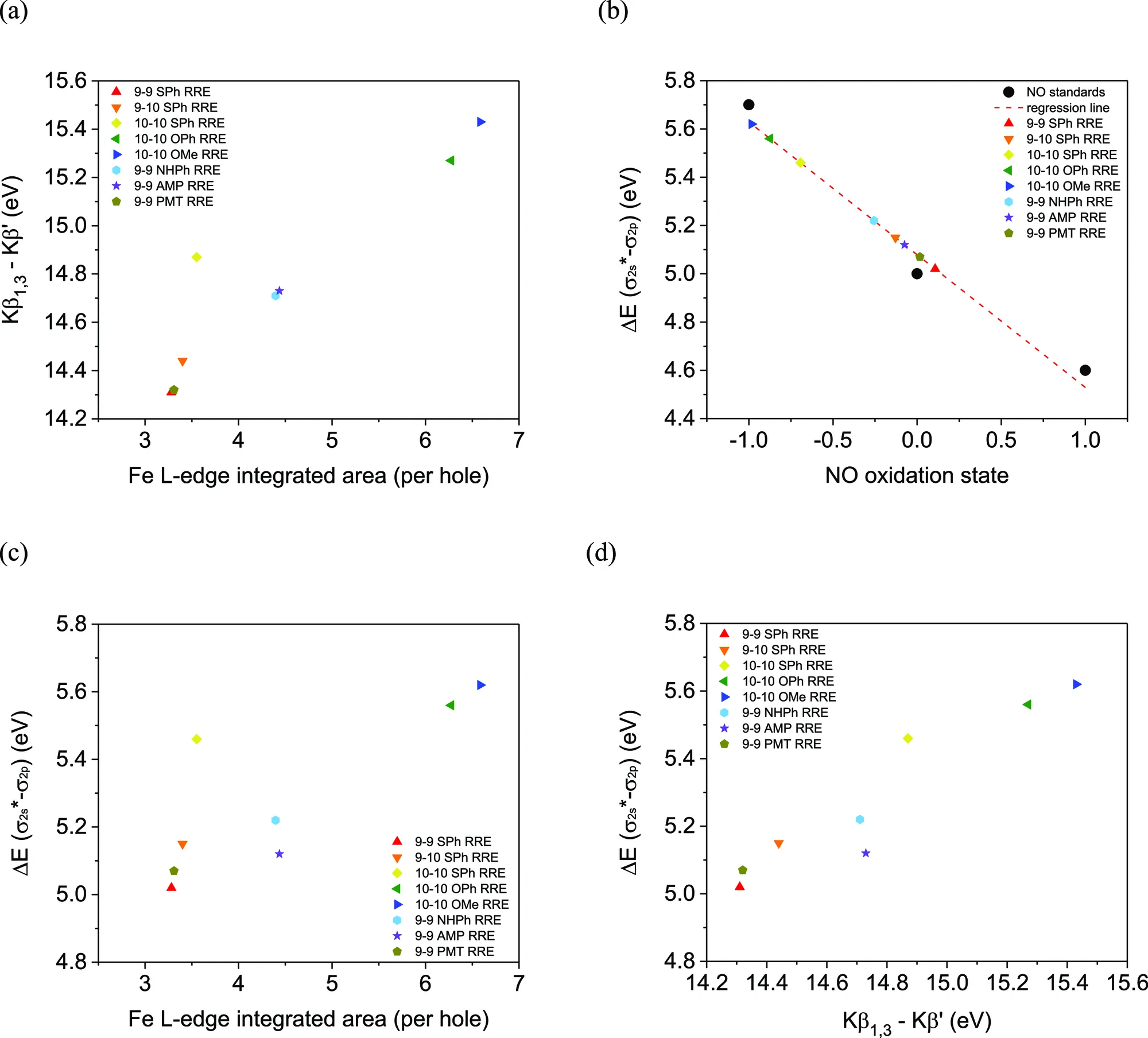
(a) The correlation between Fe L-edge peak area per hole and Kβ_1,3_–Kβ′ energy splitting. (b) Experimental assignment of NO oxidation states based on VtC Kβ_2,5_ XES fitting results by interpolation. The linear regression line follows the equation: Δ*E*_σ2s*−σ2p_ = −0.550 (NO oxidation state) + 5.079 with a standard error of 0.11.^37^ The correlation between (c) Δ*E*_σ2s*−σ2p_ and Fe L-edge peak area per hole and (d) Δ*E*_σ2s*−σ2p_ and Kβ_1,3_–Kβ′ energy splitting.

By virtue of the sensitivity of VtC XES toward the valence nature of the bound ligand, the characterization of mononuclear tetrahedral Fe–NO complexes with [SPh]^−^/[OPh]^−^ coordinated ligands demonstrates that the oxidation state of the Fe-bound NO ligand could be distinguished and estimated by VtC Kβ_2,5_ XES spectra.^37,45,58,60^ Because Kβ_2,5_ VtC emissions stem from transition from the filled NO valence 2s/2p orbitals to the Fe 1s core hole, the VtC region of RRE complexes shown in Figure S4 could be deconvoluted into five prominent peaks that are assigned from the lowest to the highest emission energy as NO σ_2s_ (Kβ″), NO σ_2s_*, NO σ_2p_ and two hybrids of π*-NO/π-SR/π-OR/π-NR_2_ ligands, where the energies near 7098 and 7103 eV are corresponding to NO σ_2s_* and NO σ_2p_ emissions, respectively (see the Supporting Information Note). In terms of the NO oxidation state as a function of the NO σ_2s_* and NO σ_2p_ energy splitting (Δ*E*_σ2s*−σ2p_ = −0.550 (NO oxidation state) + 5.079),^37^ the interpolated NO oxidation states shown in Figure 3b and Table 3 delineate that the NO^–^ character is predominant in {Fe­(NO)_2_}^10^–{Fe­(NO)_2_}^10^ RREs, and the NO oxidation state of {Fe­(NO)_2_}^9^–{Fe­(NO)_2_}^9^ RREs is in between NO^–^ and ^•^NO. In details, the gradual reduction in oxidation state of Fe-bound NO ligand from 0.11 (NO^0.11+^) to −0.26 (NO^0.26–^) and from −0.69 (NO^0.69–^) to −0.98 (NO^0.98–^) is derived from the increase in the hardness (from [SR]^−^ to [OR]^−^/[NR_2_]^−^) of coordinate ligands in {Fe­(NO)_2_}^9^–{Fe­(NO)_2_}^9^ and {Fe­(NO)_2_}^10^–{Fe­(NO)_2_}^10^ RREs, respectively. Obviously, the coordination of “hard” [OR]^−^/[NR_2_]^−^ ligands polarizes the [Fe­(NO)_2_] core to possess the dominant ionicity of Fe–OR/NR_2_ bonds, which makes the NO ligand behave like nitroxyl (NO^–^) to stabilize the [Fe­(NO)_2_] unit through the NO^δ−^ → Fe π covalent bonding pathway. Commensurate with the relationship between Fe L-edge peak area per hole and Kβ_1,3_–Kβ′ energy splitting (Figure 3a), the more NO^–^ character observed in VtC Kβ_2,5_ XES is along with the larger Kβ_1,3_–Kβ′ energy separation and Fe L-edge peak area per hole, qualitatively demonstrating covalent contribution of Fe–ER_(2)_ (E = O, N, S) bonds as the origin that Δ*E*_σ2s*−σ2p_, Kβ_1,3_–Kβ′ energy split, and Fe L-edge total intensity per hole are strongly correlated (Figure 3c,d). Based on XES probing the bonding (partially/fully occupied) orbitals of tetrahedral Fe–NO complexes, the portrait of the HOMO orbital in the [Fe­(NO)_2_] unit may be an {Fe^II^(NO^–^)_2_}^10^ electronic configuration for {Fe­(NO)_2_}^10^–{Fe­(NO)_2_}^10^ RREs. By comparison, the description of the HOMO orbital in the [Fe­(NO)_2_] unit may be a resonance hybrid of {Fe^II^(NO^–^)­(^•^NO)}^9^ and {Fe^I^(^•^NO)_2_}^9^ electronic structures for {Fe­(NO)_2_}^9^–{Fe­(NO)_2_}^9^ RREs, and the predominance of the {Fe^II^(NO^–^)­(^•^NO)}^9^ electronic structure may be attributed to the majority in ionicity character of Fe–(N/O)­R_(2)_ bonds.

In an attempt to further verify the nature of these spectral transitions, DFT computation was employed to simulate and deconvolute VtC Kβ_2,5_ XES signatures. With regard to all VtC Kβ_2,5_ XES spectra showing similar features, **9–9 SPh RRE** was selected for the discussion in details, and the others are displayed in Figures S5–S11. The calculated VtC Kβ_2,5_ XES of **9–9 SPh RRE** together with experimental data are displayed in Figure 4. The emission peak around 7097 eV corresponds primarily to a transition from N–O (σ_2s_*) molecular orbital which features an antibonding character between N (2s) and O (2s) orbitals. Meanwhile, the peak near 7102 eV is assigned to the N–O (σ_2p_) bonding orbital from an N (2p) and O (2p) head-to-head interaction. Lastly, the feature around 7109 eV is predominantly derived from the S (π)–Fe (3d)–NO (π*) bonding orbital formed by the hybridization of Fe (3d) with S (3p) and NO (π*) orbitals. According to these assignments, the energy separations between NO σ_2s_* and NO σ_2p_ orbitals are calculated and listed in Table 4. Throughout this DFT-assisted deconvolution, the Δ*E*_σ2s*−σ2p_ values are ∼4.8 eV and ∼5.1 eV for {Fe­(NO)_2_}^9^–{Fe­(NO)_2_}^9^ and {Fe­(NO)_2_}^10^–-{Fe­(NO)_2_}^9/10^ RREs, respectively, in parallel with the experimental trend (Figures 3b, S4 and Table 3). Of particular promise for VtC Kβ_2,5_ XES as a spectroscopic tool to probe electronic state of Fe-bound NO, the corresponding NO charge analysis unravels that NO^–^ character is prevailing in the {Fe­(NO)_2_}^10^–{Fe­(NO)_2_}^10^ system, whereas the ^•^NO radical feature is dominant in {Fe­(NO)_2_}^9^–{Fe­(NO)_2_}^9^ RREs, consistent with the observed tendency in N K-edge XAS (Figure 1 and Table 1).

**Figure 4:**
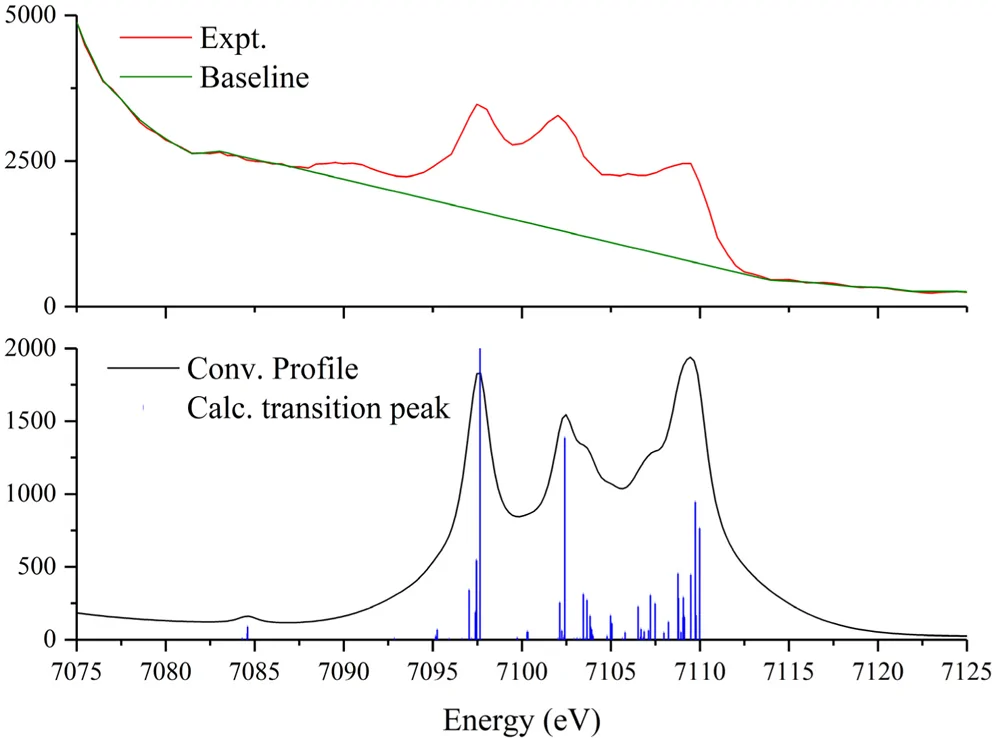
Experimental and calculated VtC Kβ_2,5_ XES of **9–9 SPh RREs**. The blue lines represent the calculated transitions, while the black line is the convoluted spectrum using a Voigt profile with a 1.5 eV Lorentzian and 10 eV Gaussian line width. To make a comparison with the experimental spectrum, the energy of the convoluted profile was aligned based on the transition of the NO σ_2s_* peak in the experimental datum.

**Table 4:** Calculated Energy Split (Δ*E*_σ2s*−σ2p_) of NO σ_2s_* and NO σ_2p_ Orbitals in VtC Kβ_2,5_ XES^b^

RREs	NO σ_2s_* (eV)	NO σ_2p_ (eV)	Δ*E*_σ2s*−σ2p_ (eV)	average NO charge (Löwdin)	average NO charge (Mulliken)
**9–9 SPh**	7097.64	7102.41	4.77	+0.0230	–0.2059
**9–10 SPh** ^a^	7098.61	7103.68	5.07	–0.2377	–0.5397
**10–10 SPh**	7098.80	7103.92	5.12	–0.6362	–0.9882
**9–9 PMT**	7099.46	7104.25	4.79	+0.0029	–0.2515
**9–9 NHPh**	7099.09	7103.90	4.81	–0.0574	–0.3883
**9–9 AMP**	7098.80	7103.56	4.76	–0.0842	–0.4060
**10–10 OPh**	7098.73	7103.80	5.07	–0.7006	–1.1089
**10–10 OMe**	7097.58	7102.75	5.17	–0.7780	–1.2343

a The average value of two strong transitions at 7098.24 and 7098.98 eV.

b The energy shift of the computed spectra is based on the experimental spectra for a series of {Fe­(NO)_2_}^9/10^–{Fe­(NO)_2_}^9/10^ RREs.

More importantly, the synergism between NO^–^/^•^NO balance within the [Fe­(NO)_2_] unit and ionicity-covalency competition within the Fe–ER_(2)_ bond was explored by DFT computation. The molecular orbital energy diagrams of all [ER_(2)_]^−^-bridged {Fe­(NO)_2_}^9/10^–{Fe­(NO)_2_}^9/10^ complexes are displayed in Figures S12–S19. In the unrestricted open-shell calculation, the corresponding contributions of Fe (3d) and NO (π*) orbitals are listed in Tables S2 and S3, and the overall statistics are summarized in Table S4. The average Fe (3d) population in the corresponding molecular orbitals featured with bridged atoms of S, N, and O are ∼10.8*e* ^–^, ∼10.4*e* ^–^, and ∼9.3*e* ^–^, respectively. The result indicates the covalent character is in the order of Fe–SR > Fe–NR > Fe–OR bonds, consistent with Löwdin and Mulliken analyses based on Fe 3d-orbital population (Table 2). Meanwhile, the NO (π*) contribution in the corresponding molecular orbitals of {Fe­(NO)_2_}^9^–{Fe­(NO)_2_}^9^, {Fe­(NO)_2_}^9^–{Fe­(NO)_2_}^10^, and {Fe­(NO)_2_}^10^–{Fe­(NO)_2_}^10^ RREs are ∼5.1*e* ^–^, ∼5.6*e* ^–^, and ∼6.5*e* ^–^, implicating that NO electronic structure of {Fe­(NO)_2_}^9^–{Fe­(NO)_2_}^9^ and {Fe­(NO)_2_}^10^–{Fe­(NO)_2_}^10^ systems is ^•^NO and NO^–^, respectively. In combination with VtC Kβ_2,5_ XES analyses, the increase in NO (π*) contribution resulting from 1*e* ^–^/2*e* ^–^ reduction of [SR]^−^-bridged {Fe­(NO)_2_}^9^–{Fe­(NO)_2_}^9^ RRE (**9–9 SPh RRE**, 5.153*e* ^–^; **9–10 SPh RRE**, 5.644*e* ^–^; **10–10 SPh RRE**, 6.135*e* ^–^) is indicative of the decreasing Fe → ^•^NO π back bonding along with the increasing NO^–^ → Fe π-donating interaction. For {Fe­(NO)_2_}^10^–{Fe­(NO)_2_}^10^ RREs (**10–10 SPh RRE**, 6.135*e* ^–^; **10–10 OPh RRE**, 6.564*e* ^–^; **10–10 OMe RRE**, 6.942*e* ^–^), the incremental ionicity of Fe–OR bonds (vs Fe–SR bonds) leads to the incremental contribution of the NO^–^ → Fe π-donating interaction. In comparison with soft [SR]^−^-bridged ligand, the dominant ionicity of Fe–NR_2_ bonds (vs Fe–SR bonds) in {Fe­(NO)_2_}^9^–{Fe­(NO)_2_}^9^ RREs results in the increase in NO (π*) contribution (**9–9 SPh RRE**, 5.153*e* ^–^; **9–9 PMT RRE**, 5.091*e* ^–^; **9–9 NHPh RRE**, 5.387*e* ^–^; **9–9 AMP RRE**, 5.487*e* ^–^), suggesting that, for the preservation of a highly covalent {Fe­(NO)_2_}^9^ core, the enhancement of NO^–^ → Fe π-donating contribution along with the decrease in the contribution of Fe → ^•^NO π back bonding induced by the hard nature of the N-donor ligand realizes Fe electron homeostasis evidenced by the reported pre-edge energy within the narrow range of {Fe­(NO)_2_}^9^ DNICs (7113.5–7114.2 eV) and {Fe­(NO)_2_}^10^ DNICs (7113.0–7113.4 eV).^33,34,70^ Overall, it is demonstrated that the balance between NO^–^ → Fe π-donating and Fe → ^•^NO π-backbonding interactions in the [Fe­(NO)_2_] unit(s) is modulated by the hard/soft-ligand-induced ionicity-covalency competition imposed on iron center(s) in the isolable, thermally stable [ER_(2)_]^−^-bridged {Fe­(NO)_2_}^9/10^–{Fe­(NO)_2_}^9/10^ RREs (E = S, N, O).

Ligand-controlled ionicity-covalency competition is commonly invoked in Fe-mediated NO chemistry. To further understand how the more ionic/covalent interaction of the first coordination sphere with the Fe center modulates the strength of NO^–^ → Fe π-donating interaction responsible for [Fe­(NO)]_2_ management or ON–NO coupling, we make a comparison of {Fe­(NO)_2_}^9^ DNIC with a “non-classical” hs-{Fe­(NO)}^7^ (hs = high spin) complex. In a highly covalent {Fe­(NO)_2_}^9^ unit, NO^–^/^•^NO balance is governed by the ligand-controlled ionicity-covalency competition imposed on the iron center. In this sense, the {Fe­(NO)_2_}^9^ core could be considered as a stable transfer/storage unit in GSTs and the building block in [Fe–S] cluster repair. As characterized by the rhombic EPR signal with *g*_av_ = ∼2.025, average IR_νNO_ as ∼1740 cm^–1^, and Fe–N–O bond angle close to 169°,^33,34,70^ the coordination of hard N/O-donor ligands leads to an increase in the *Z*_eff_ of the iron center, which realizes the strong NO^–^ → Fe π-donating interaction to maintain Fe electronic homeostasis for preserving the {Fe­(NO)_2_}^9^ core. In contrast, for the “non-classical” hs-{Fe­(NO)}^7^ complex mimicking the first intermediate of NO reduction catalyzed by flavodiiron NO reductase, the acute Fe–N–O bond angle (149°), axial EPR pattern (*g*_eff_ = 4, 2 for S_t_ = 3/2), and the lowest NO stretching frequency (1689 cm^–1^) suggest Fe­(III)–^3^NO^–^ electronic structure, where a reduction in π-donicity from ^3^NO^–^ to Fe­(III) results in the weak Fe–NO/N–O bonds and the partial oxidation of the phenolate coligands. This weak NO^–^ → Fe π-donating interaction means that a “non-classical” hs-{Fe­(NO)}^7^ complex has a large amount of unpaired electron density in the bound ^3^NO^–^ π* orbitals, which lowers the activation barrier for ON–NO coupling and is suitable for N_2_O formation.^63^

### 2.5 Implications of Ligand-Controlled NO^–^/^•^NO Balance for DNIC-Mediated Translational Functionality

With the Fe/NO electronic states in the dinitrosyl iron unit ([Fe­(NO)_2_]) and the interplay among Fe, NO, and S/N/O-containing bridge ligands being “physically” illustrated by the combined X-ray spectroscopic techniques and DFT computation, we apply this result, NO^–^/^•^NO balance within [Fe­(NO)_2_] unit modulated by the ligand-controlled ionicity-covalency competition imposed on the iron center, to comprehensively understand the system in which the covalent [Fe­(NO)_2_] unit supported by redox-active hard/soft ligands acts as a revolutionary platform to resemble transformative reactivity and subsequently establish the relationship between electronic structure and DNIC-mediated translational functionality (Scheme 2).

**Scheme 2:**
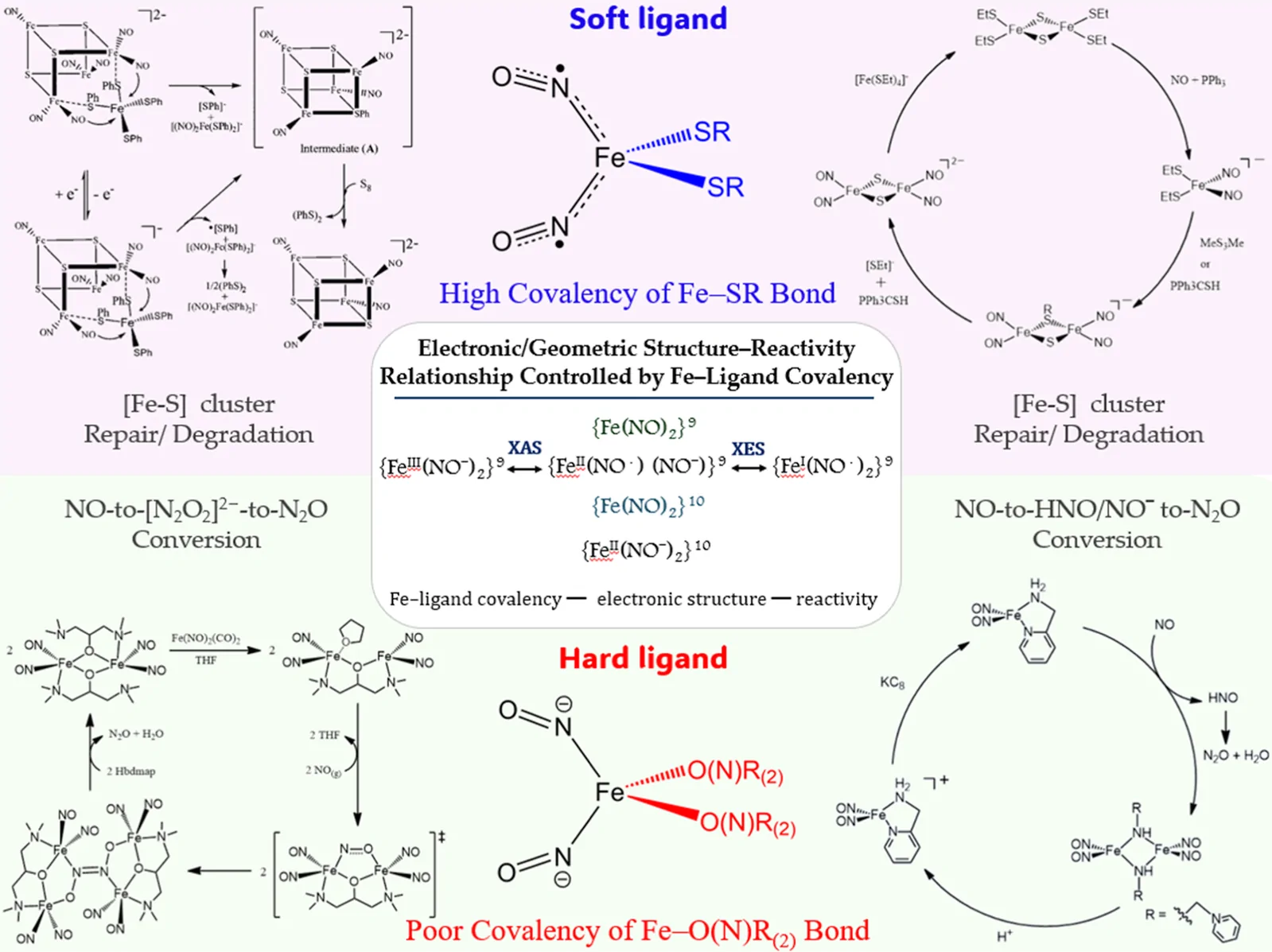


### 2.6 DNIC as Conservator of [Fe–S] Clusters and a Point of NO/H_2_S Crosstalk

Commensurate with covalency-induced resonance hybrid of {Fe^III^(NO^–^)_2_}^9^ and {Fe^II^(NO^–^)­(NO^•^)}^9^ electronic structures of {Fe­(NO)_2_}^9^ core, repair of DNICs back to the original [2Fe–2S] and [4Fe–4S] clusters could be achieved via preassembled clusters, [(NO)_4_Fe_2_(μ-S)_2_]^2–^ and [(NO)_4_Fe_4_(μ-S)_4_]^2–^, generated in the course of intermolecular NO radical-thiyl radical exchange (or/and nitroxyl–thiolate exchange) between [(NO)_4_Fe_2_(μ-S)­(μ-SEt)]^−^/[(NO)_7_Fe_4_(μ-S)_3_]^2–^ and [SEt]^−^/[Fe­(SPh)_4_]^2–^ accompanied by oxidative addition of sulfur-donor source (HSCPh_3_ or S_8_). Along with the formation of {Fe­(NO)_2_}^9^ DNICs as byproducts, the subsequent thiyl/thiolate capture of [Fe­(SR)_4_]^−^ (R = Et, SPh) by [(NO)_4_Fe_2_(μ-S)_2_]^2–^ and [(NO)_4_Fe_4_(μ-S)_4_]^2–^ generates [2Fe–2S] and [4Fe–4S] clusters, respectively.^64,65^ Thanks to the uniqueness of the noninnocent nature of NO/[SR]^−^ soft ligands and covalent character of ON–Fe–SR bonds, the biomimetic degradation–repair cycle demonstrates that DNICs might function not only as a “conservator” of the damaged [Fe–S] clusters but also as a “pacificator” to relieve nitrosative stress for cellular redox homeostasis.

In HS-Fe-NO chemistry, {Fe­(NO)_2_}^9^ [(HS)_2_Fe­(NO)_2_]^−^ DNIC (Fe K-edge pre-edge energy at 7113.5 eV; an isotropic EPR signal with *g*_av_ = 2.028, *a*_NO_ = 2.8 G) was stable in THF and isolated as thermally stable solid at room temperature. Further, DFT computation shows that the triplet NO^–^ bound to [Fe^III^–SH] motif in {Fe^III^(NO^–^)_2_}^9^ contribution acts as an effective regulator to reduce the *Z*_eff_ of iron to prevent the coordinate hydrosulfide from reductive elimination. More importantly, hydrosulfide-bound DNIC, [(HS)_2_Fe­(NO)_2_]^−^, rapidly transforms into Roussin’s red salt [(μ-S)­Fe­(NO)_2_]_2_^2–^, the building precursor of [2Fe–2S] clusters, upon dissolution in water, concomitantly releasing H_2_S characterized/quantified by the H_2_S probe NBD–SCN (NBD = nitro-benzofurazan). Meanwhile, the reversible transformation of [(μ-S)­Fe­(NO)_2_]_2_^2–^ to [(HS)_2_Fe­(NO)_2_]^**®**^ was observed in the presence of H_2_S. In cooperation with NO radical-thiyl radical exchange (or/and nitroxyl–thiolate exchange) pathways, this finding, the reversible H_2_S binding at DNIC, signifies that NO–H_2_S crosstalk hold by DNIC might be at the very inception of repairing [Fe–S] clusters while hydrosulfide-bound {Fe­(NO)_2_}^9^ DNIC also behaves as an alternative (hydro)­sulfide source/carrier for synthesis of [Fe–S] clusters.^66^

### 2.7 DNIC as a Translational Model of Flavodiiron NO Reductase

As supported by the concept that fluctuation in electron density of the Fe center imposed by the hard/soft nature of the coordinate [OR]^−^/[SR]^−^ ligands is buffered by the noninnocent character of the NO ligand, the high covalent contribution of Fe–S bonds effectuates full delocalization of the mixed-valence {Fe­(NO)_2_}^9^–{Fe­(NO)_2_}^10^ [(NO)_4_Fe_2_(μ-SR)_2_]^−^ (R = Et, *tert*-Bu) RREs (IRν_NO_ at 1673, 1655 cm^–1^, the axial EPR signal with *g* = 2.006, 1.977 at 77 K).^39,67^ Contrarily, the dominant ionicity of the Fe–O_μ‑bdmap_ bond polarizes electronic communication of the adjacent [Fe­(NO)_2_] units and then builds/stabilizes the electronically localized, singly alkoxide-bridged {Fe­(NO)_2_}^10^–{Fe­(NO)_2_}^9^ dinuclear DNIC [(NO)_2_Fe­(μ-bdmap)­Fe­(NO)_2_(THF)] (bdmap = 1,3-bis­(dimethylamino)-2-propanolate) with the [Fe­(NO)_2_]^9^ unit coordinated by THF, as corroborated by the isotropic EPR signal with *g* = 2.016 (77 K) and IRν_NO_ at 1762, 1701, 1682, 1650 cm^–1^. The poor covalency of the Fe–O_μ‑bdmap_ bond makes the {Fe­(NO)_2_}^10^ unit serve as an electron transfer channel for electrophilic attack of an exogenous NO molecule and the THF-bound {Fe­(NO)_2_}^9^ unit acts as an electron buffering reservoir for self-accommodation of electron redistribution during NO activation, guiding electron transfer from the {Fe­(NO)_2_}^10^ center to the bridging NO radical to furnish the proposed {Fe­(NO)_2_}^9^–{Fe­(NO)_2_}^9^ dinuclear DNIC [(NO)_2_Fe­(μ-bdmap)­(κ^2^-N,O–NO^–^)­Fe­(NO)_2_] intermediate. In contrast to free NO, DFT computation unravels that buildup of spin density on a side-on nitroxyl-bridged ligand (*N*_NO_ = −1.01 spins, *O*_NO_ = −0.40 spins) initiates intermolecular [^−^ON–NO^–^] coupling, establishing a facile pathway for NO-to-[N_2_O_2_]^2–^ conversion to generate a *trans*-*N*,*O*-hyponitrite-bridged {[Fe­(NO)_2_]_2_(μ-bdmap)}_2_(κ^4^-N_2_O_2_) complex characterized by IR spectroscopy (ν_N–O(κ4‑N2O2)_, 1081 cm^–1^ (KBr)) and single-crystal X-ray diffraction. The Fe K-edge pre-edge energy at 7113.9 eV demonstrates that the hard nature of bdmap/hyponitrite ligands not only polarize the Fe center to enhance the {Fe^III^(NO^–^)_2_}^9^ population but also justifies the weak antiferromagnetic coupling (*J*_μ‑bdmap_ = −69.8 ± 4.4 cm^–1^, *J*_κ4–N2O2_ = −33.8 ± 2.2 cm^–1^) among the four {Fe­(NO)_2_}^9^ centers along with a broad EPR signal with *g* = 2.025 and a half-field signal with *g* = 4.28. Subsequent treatment of 2 equiv of Hbdmap to complex {[Fe­(NO)_2_]_2_(μ-bdmap)}_2_(κ^4^-N_2_O_2_) regenerates {Fe­(NO)_2_}^9^–{Fe­(NO)_2_}^9^ DNIC [(NO)_2_Fe­(μ-bdmap)_2_Fe­(NO)_2_] and completes the synthetic cycle for NO-to-[N_2_O_2_]^2–^-to-N_2_O transformation with N_2_O yield as 86% quantified by GC.^68,69^

### 2.8 DNIC as an Active Platform for NO Reduction to Yield Nitroxyl (NO^–^)

In response to ligand-controlled NO^–^/^•^NO balance in the [Fe­(NO)_2_] unit, the interaction between NO and a covalent [Fe­(NO)_2_] unit may hold the potential to explore the impact of the hard supporting ligand on NO-to-HNO/NO^–^ conversion. In the case of the mononuclear {Fe­(NO)_2_}^10^ DNIC [(AMP)­Fe­(NO)_2_] (AMP = 2-(aminomethyl)­pyridine), binding of NO toward the tetrahedral iron center is proposed to afford a {Fe­(NO)_3_}^11^ [(AMP)­Fe­(NO)_3_] intermediate. Powered by the hard nature of the AMP supporting ligand, the reduction capability of the {Fe­(NO)_2_}^10^ motif further enhances the nitroxyl character of the third NO ligand, leading to the buildup of a {Fe­(NO)_2_}^9^–[NO^–^] electronic structure. The chemistry, poor covalency of Fe–N_AMP_ bonds that promotes intramolecular electron transfer from the {Fe­(NO)_2_}^10^ core to the NO radical, is similar to the reductive activation of NO by the electronically localized {Fe­(NO)_2_}^10^–{Fe­(NO)_2_}^9^ DNIC [(NO)_2_Fe­(μ-bdmap)­Fe­(NO)_2_(THF)] described above. The prevailing population of the {Fe­(NO)_2_}^9^ electronic state in the {Fe­(NO)_3_}^11^ [(AMP)­Fe­(NO)_3_] intermediate enhances the acidity of the amino group ready for protonation of the {Fe­(NO)_2_}^9^-bound [NO^–^] followed by the release of HNO_(g)_, of which the rapid dimerization yields N_2_O_(g)_ (GC, 80% yield) characterized by IR spectroscopy (2223 cm^–1^). At the same time, the bridging tendency of deprotonated amino group initiates the dissociation of the chelating pyridyl moiety and facilitates the assembly of amido-bridged dinuclear {Fe­(NO)_2_}^9^–{Fe­(NO)_2_}^9^ DNIC [(NO)_2_Fe­(μ-dAMP)_2_Fe­(NO)_2_] (dAMP = deprotonated AMP).^70^

## 3 Conclusion

The integration of N K-edge, Fe L-edge XAS, Fe K-edge HERFD-XANES, Kβ_1,3_ mainline, VtC Kβ_2,5_ XES, and DFT computation authenticates the impact of Fe–ER_(2)_ bonding character (ionicity vs covalency) on the NO oxidation state and the noninnocent character of the Fe-bound NO^δ−^ ligand to alleviate electron deficit of the polarized Fe center imposed by ionic dominance of Fe–(N/O)­R_(2)_ bonds. The correlation among Δ*E*_σ2s*−σ2p_, Kβ_1,3_–Kβ′ energy split and Fe L-edge total intensity per hole unveils that resonance hybrid of {Fe^II^(NO^–^)­(^•^NO)}^9^ and {Fe^III^(NO^–^)_2_}^9^ electronic structures in the LUMO orbital is indicated by Fe K-edge pre-edge HERFD–XANES spectra. VtC Kβ_2,5_ XES spectra suggest that {Fe^II^(NO^–^)­(^•^NO)}^9^ and {Fe^I^(^•^NO)_2_}^9^ electronic contributors are resonantly hybridized in the HOMO orbital of the {Fe­(NO)_2_}^9^ moiety. While the electronic structure in HOMO/LUMO orbitals of the {Fe­(NO)_2_}^10^ motif is delineated as {Fe^II^(NO^–^)_2_}^10^, the [N/OR_(2)_]^−^-induced polarization imposed on the Fe center through the delocalized covalent nature of Fe–NO bonds enhances the population of {Fe^III^(NO^–^)_2_}^9^ and {Fe^II^(NO^–^)­(^•^NO)}^9^ electronic contributors in LUMO and HOMO orbitals, respectively. Collectively, NO functions as an electron sponge to buffer the hard/soft polarization imposed on the Fe center by the coordinate ligand(s), leading to electronic homeostasis of Fe center(s) to preserve the highly covalent [Fe­(NO)_2_] unit(s) in DNICs/RREs. Through delicate competition between ionicity and covalency in Fe–ER_(2)_ bondings (E = O, N, S), the balance between NO^–^ → Fe π-donating and Fe → ^•^ NO π-backbonding interactions enables the covalent [Fe­(NO)_2_] units to imitate/execute H_2_S/NO communication, degradation/repair of [Fe–S] clusters, and translational functionality for NO-to-[N_2_O_2_]^2–^-to-N_2_O and NO-to-HNO/NO^–^ transformations, underscoring the enduring power of electronic control as a unifying concept to shape DNIC chemistry and beyond.

## 4 Experimental Section

Manipulations, reactions, and transfers were conducted under N_2_ according to Schlenk techniques or in a glovebox (N_2_ gas). Solvents were distilled under N_2_ from appropriate drying agents (acetonitrile from CaH_2_–P_2_O_5_; diethyl ether, hexane, and tetrahydrofuran (THF) from sodium benzophenone) and stored in dried, N_2_-filled flasks over 4 Å molecular sieves. N_2_ was purged through these solvents before use. The solvent was transferred to the reaction vessel via a stainless cannula under positive pressure of N_2_. Potassium, methanol, phenol, thiophenol, aniline, 4-(aminomethyl)­pyridine, 2-pyridinemethanethiol, nitrosonium tetrafluoroborate (Aldrich), tetraphenylphosphonium chloride (Alfa), bis­(triphenylphosphoranylidene)­ammonium chloride ([PPN]­[Cl], Fluka), iron pentacarbonyl (Strem), tetramethylethylenediamine, and 18-crown-6-ether (TCI) were used as received. Complexes [Fe­(CO)_2_(NO)_2_], [(TMEDA)­Fe­(NO)_2_], [Fe_2_(μ-SPh)_2_(NO)_4_] (**9–9 SPh RRE**), [Fe_2_(μ-SPh)_2_(NO)_4_]^−^ (**9–10 SPh RRE**), [Fe_2_(μ-SPh)_2_(NO)_4_]^2–^ (**10–10 SPh RRE**), [Fe_2_(μ-OPh)_2_(NO)_4_]^2–^ (**10–10 OPh RRE**), [Fe_2_(μ-OMe)_2_(NO)_4_]^2–^ (**10–10 OMe RRE**), [Fe_2_(μ-NHPh)_2_(NO)_4_] (**9–9 NHPh RRE**), [Fe_2_(μ-AMP)_2_(NO)_4_] (**9–9 AMP RRE**) were synthesized based on the previous literature reports.^33,34,70–72^ No uncommon hazards were noted. Infrared spectra of the ν­(NO) stretching frequencies were recorded on a PerkinElmer model spectrum one B spectrophotometer with KBr pellet. UV–vis spectra were recorded on a Jasco V-570. Analyses of carbon, hydrogen, and nitrogen were conducted with a CHN analyzer (Heraeus).

### 4.1 Preparation of [Fe_2_(μ-PMT)_2_(NO)_4_] (**9–9 PMT RRE**)

THF solution (20 mL) of as-prepared [Fe­(CO)_2_(NO)_2_] (1 mmol) was added to 50 mL of Schlenk flask containing 2-pyridinemethanethiol (125 mg, 1 mmol) by a cannula under a N_2_ atmosphere. The reaction mixture was allowed to stir for 3 h at ambient temperature. The resulting brown solution was concentrated under vacuum, and the solution was filtered through Celite to remove the insoluble solid. The addition of hexane (30 mL) led to the precipitation of the dark brown solid [Fe_2_(μ-PMT)_2_(NO)_4_] (232 mg, 0.48 mmol, 48%). Dark brown crystals suitable for X-ray diffraction analysis were grown from the THF solution of complex **9–9 PMT RRE** layered with hexane at room temperature for 1 week. IR ν_NO_ (cm^–1^) 1811 (w), 1778 (s), 1751 (s) (THF); 1781 (s), 1733 (vs) (KBr). Absorption spectrum (THF) [λ_max_, nm (ε, M^–1^ cm^–1^)]: 350 (6560), 366 (6020). Anal. Calcd for Fe_2_C_12_H_12_N_6_O_4_S_2_: C, 30; N, 17.5; O, 13.3. Found. C, 30.7; N, 16.9; O, 13.6; H, 2.73.

### 4.2 Preparation of [PPN]_2_[(μ-SPh)­Fe­(SPh)_2_]_2_

To a Schlenk flask containing FeCl_2_·4H_2_O (200 mg, 1 mmol) and [Na]­[SPh] (420 mg, 3.2 mmol) was added 20 mL of CH_3_CN solution under positive N_2_ pressure. The reaction mixture was stirred for 30 min at ambient temperature. The CH_3_CN solution (5 mL) of [PPN]­[Cl] (600 mg, 1.05 mmol) was added and then the solution kept under stirring overnight. The resulting black solution was concentrated under vacuum and filtered through Celite to remove the insoluble solid. Diethyl ether (30 mL) was added to precipitate the dark black solid. Dark brown crystals suitable for X-ray diffraction analysis were grown from the CH_3_CN solution of complex [PPN]_2_[(μ-SPh)­Fe­(SPh)_2_]_2_ layered with diethyl ether at room temperature for 1 week. Absorption spectrum (CH_3_CN) [λ_max_, nm (ε, M^–1^ cm^–1^)]: 225 (4080), 230 (3900), 268 (1208), 275 (1072). Anal. Calcd for Fe_2_C_108_H_90_N_2_S_6_P_4_: C, 70.30; N, 1.50; H, 4.88. Found. C, 70.67; N, 1.63; H, 4.79.

### 4.3 Preparation of [PPN]­[Fe­(OPh)_4_]

FeCl_3_ (162 mg, 1 mmol), [K]­[OPh] (662 mg, 5 mmol), and [PPN]­[Cl] (574 mg, 1 mmol) were loaded into a Schlenk tube and dissolved in dry acetonitrile (10 mL). The reaction mixture was stirred for 2 h at ambient temperature. The resulting red-orange solution was filtered through Celite to remove the insoluble solid, presumably, KCl. Diethyl ether (25 mL) was then added slowly to layer above the filtrate. The flask was tightly sealed and kept in the refrigerator at −20 °C for 2 weeks to yield the dark orange crystals **[PPN]­[Fe­(OPh)**_4_ **]** (0.74 g, 76%) suitable for X-ray diffraction analysis. Absorption spectrum (CH_3_CN) [λ_max_, nm (ε, M^–1^ cm^–1^)]: 222 (6510), 265 (1143), 272 (1355) 278 (1042). Anal. Calcd for C_60_H_50_FeNO_4_P_2_: C, 74.54; H, 5.21; N, 1.45; O, 6.62. Found. C, 74.07; H, 5.35; N, 1.49; O, 7.01.

### 4.4 X-ray Absorption Measurements

The N K-edge and Fe L-edge X-ray absorption experiments were carried out at BL-20A high-energy spherical grating monochromator beamline, National Synchrotron Radiation Research Center, Hsinchu, Taiwan. All samples were ground to powder from single crystals, stuck to a conducting tape, and then put into an ultrahigh vacuum chamber (10^–9^ Torr). The spectra were recorded in total electron yield mode with a microchannel plate as the detector. Every spectrum was calibrated against the known N_1s_ → π* absorption energy at 401.0 eV of BN reference and Fe L_3_-edge absorption peak at 708.5 eV of the Fe_2_O_3_ standard. For the N K-edge spectrum, the background was removed by fitting a straight line to the pre-edge region and subtracting this straight line from the entire spectrum. Based on the repeated calibration and sample measurements during different experimental runs, we estimate the standard deviation of energy as 0.1 eV. Fe L-edge XAS data were collected over the range from 695 to 760 eV with a step size of 0.1 eV. Two arc tangents were subtracted from the normalized spectrum: absorption = [tan^–1^(*k*(energy-*E*_1_) + π/2) × 2/3 × 1/π] + [tan^–1^(*k*(energy-*E*_2_) + π/2) × 1/3 × 1/π], where *k* was obtained from experimental fit and *E*_2_ = *E*_1_ + 12.3 eV (*E*_2_ is the energy of L_2_ feature relative to *E*_1_ energy of L_3_ feature split by spin–orbit coupling) was used to model L_2_ and L_3_ edge jumps.^49–51^ According to multiple calibration and measurements of the same sample on different scans, standard deviation of energy as 0.15 eV was estimated.

### 4.5 Kβ X-ray Emission Measurements

The measurements of Fe K-edge HERFD–XANES, Kβ_1,3_ mainline, and VtC Kβ_2,5_ XES spectra were carried out at beamline BL 12XU of Japan Synchrotron Radiation Research Institute at the Super Photon ring-8 GeV (SPring-8) in Harima Science Park City, Hyogo Prefecture, Japan. The incident energy was selected using a Si(111) double-crystal monochromator, leading to a photon flux of 1.2 × 10^13^ photon/s on the sample position. The energy resolution Δ*E*/*E* was about 2 × 10^–4^, and the associated spectrometer energy resolution was around 0.35 at 7112.0 eV. Incident energy calibration was conducted using the first inflection point at 7112.0 eV of the Fe foil spectrum. Measurements were conducted with a Johann-type X-ray emission spectrometer in the horizontal plane, where the sample, Ge crystal analyzers, and photodiode detector were arranged in vertical Rowland circles. The HERFD–XANES spectra at Fe K-edge were obtained by recording the intensity of the Kβ_1,3_ emission-line maximum as a function of the incident energy. Emission energy discrimination was performed using the (620) reflection of spherical Ge crystal analyzers. The partial fluorescent yield was monitored by a photodiode detector installed at a 90° scattering angle and at 45° to the sample surface. During each XANES scan, the undulator gap was kept at a fixed position, and only the monochromator angle was changed.

Kβ_1,3_ mainline spectra were recorded off-resonance at an incident energy of 7.5 keV in the range of 7020.0–7080.0 eV with a step width of 0.1 eV. For VtC Kβ_2,5_ XES data collection, the energy scans were performed in the range from 7070.0 to 7130.0 eV with an incident energy of 7.2 keV and a step width of 0.1 eV. To achieve a satisfactory signal-to-noise ratio, four 10 min scans measured were averaged, utilizing different spots for each scan on the homogeneous sample. A helium-filled bag with Kapton windows was placed in between the sample, the analyzers, and the detector to minimize attenuation of the fluorescence signal due to the air absorption. Since VtC XES region is superimposed with the slope of Kβ_1,3_ mainline, all four VtC Kβ_2,5_ XES spectra have to be background corrected. For this purpose, the Kβ_1,3_ mainline slope was fitted by a pseudo-Voigt function using Origin-2016 software, and the experiment was subtracted by the resulting fit.

### 4.6 Computation Method

All crystal structures were downloaded from Cambridge Crystallographic Data Centre (CCDC). The geometric structures were first optimized in periodic DFT program of CRYSTAL23,^73^ and then the electronic structure calculations and simulation of XES were carried out in ORCA6.1 programs.^74^ Due to the unperfect experimental single crystal diffraction data, like absorption correction, data resolution, experimental temperature, disorder or twin, electronic calculations only on the RREs part could not give consistent results. Thus, all crystal structures were optimized under fixed cell constants and using r2SCAN^75^ exchange functional accompany with an extra-large grid setting and with a Gaussian-type POB-TZVP-rev2 basis set.^76^ The total energy convergence was set to 10^–7^ hartree. To ensure enough sampling in *k*-space (reciprocal space), the shrinking factors were set to 4 × 4 × 4, which makes the *k*-points in the irreducible brillouin zone 36, 30, and 18 for *P*1̅, *P*2_1_/*c* and *P*4̅, respectively. Spin polarized calculations were examined in **9–9 SPh RRE** and **10–10 SPh RRE**, but there were no differences in the final results due to no net unpaired electrons in the whole crystal. However, in the case of **9–10 SPh RRE**, spin polarization of 1 at Fe­(NO)_2_}^9^–{Fe­(NO)_2_}^10^ was used and the final spin densities at two Fe sites were 0.715 e/Å^3^ and 0.716 e/Å^3^. The selected bond distances are listed in Table S1. In ORCA calculation, the unrestricted Kohn–Sham (uks) calculations were performed in TPSSh^77^ and r2SCAN exchange functionals including dispersion correction of D3 ^78^ and Conductor-like Polarizable Continuum Model with a water solvent. The relativistic effect was approximated by a zeroth order regular approximation (ZORA), and the ZORA-def2-TZVP­(-f) basis set and the SARC/J auxiliary basis set were used for all calculations. The broken symmetry state of every compound was obtained by using the Yamaguchi approximate spin projection method.^79–83^ The spin-obit coupling effect was included in the calculation of XES. The spectra intensities were calculated by including the complete semiclassical multipolar moments. Because the TPSSh functional exhibits the best consistency with experimental data, we will primarily discuss these calculated results.

### 4.7 Crystallography

The ORTEP drawings, crystallographic data and structure refinement parameters are summarized in Figure S20 and Table S5–S7. The crystals of complexes **9–9 PMT RRE**, **[PPN]­[Fe­(OPh)**_4_ **]**, and **[PPN]**_2_ **[(μ-SPh)­Fe­(SPh)**_2_ **]**_2_ chosen for X-ray diffraction studies are measured in sizes of 0.16 × 0.03 × 0.02 mm, 0.15 × 0.14 × 0.05 mm, and 0.11 × 0.07 × 0.02 mm, respectively. Each crystal was mounted on a glass fiber and quickly coated in epoxy resin. Unit-cell parameters were obtained by least-squares refinement. Diffraction measurement for all complexes was carried out on a Bruker Kappa APEXII CCD diffractometer with graphite-monochromated Cu Kα radiation (λ = 1.54184 Å). Least-squares refinement of the positional and anisotropic thermal parameters of all non-H atoms and fixed H atoms was based on *F* ^2^. A SADABS absorption correction was made. The SHELXTL structure refinement program was employed.

The crystal structure of **[PPN]**_2_ **[(μ-SPh)­Fe­(SPh)**_2_ **]**_2_ exhibits significant crystallographic disorder involving both the PPN cations and the dinuclear iron complex. Accordingly, the appropriate SADI distance restraints and SIMU displacement restraints were applied during the refinement to model the disordered components while making geometries and atomic displacement parameters chemically reasonable. The complete refinement details are provided in the deposited CIF file.

## Supplementary Material



## Notes

N K-edge XAS, Fe L-edge XAS, Kβ_1,3_ mainline XES, VtC Kβ_2,5_ XES, the calculated VtC Kβ_2,5_ XES spectra and the corresponding orbitals of all RREs as well as ORTEP drawings/labeling schemes of **9–9 PMT RRE, [PPN]­[Fe­(OPh)**_4_ **], [PPN]**_2_ **[(μ-SPh)­Fe­(SPh)**_2_ **]**_2_; and optimized geometry, Fe (3d) and NO (π*) contributions of selected corresponding orbitals, statistics values of Fe (d) and NO (π*) contributions in the corresponding orbitals, and crystallographic data (PDF)
