# Supplementary material for: Impact of the Metal–Ligand Bonding Nature on Electronic States of {Fe(NO)2}9/10–{Fe(NO)2}9/10 RREs Unraveled by the Combined X‑ray Spectroscopic Analyses: Relevance to DNIC-Mediated Translational Functionality
Source: Inorg Chem. 2026 Oct 19;65(43):25300–14. doi: 10.1021/acs.inorgchem.6c03317 (PMC13733267; doi:10.1021/acs.inorgchem.6c03317)
Supplement: Supplementary file 1 [file ic6c03317_si_001.pdf]

Supporting Information

Impact of Metal-Ligand Bonding Nature on Electronic States of $\{Fe(NO)_2\}^{9/10}$-$\{Fe(NO)_2\}^{9/10}$ RREs Unraveled by the Combined X-Ray Spectroscopic Analyses: Relevance to DNIC-Mediated Translational Functionality

Sen-Hung Hsieh,[†] Jia-Yu Lin,[‡] Feng-Tian Chang,[†] Chi-Yen Tung,[†] Nozomu Hiraoka,[§,#] Shu-Chih Haw,[§] Fu-Te Tsai,*[†¶] I-Jui Hsu,*[‡] Wen-Feng Liaw*[†]

[†] Department of Chemistry, National Tsing Hua University, Hsinchu 300044, Taiwan

[‡] Research and Development Center for Smart Textile Technology, Department of Molecular Science and Engineering, National Taipei University of Technology, Taipei 106344, Taiwan

[¶] Department of Applied Chemistry, National Chiayi University, Chiayi, 600355, Taiwan

[§] National Synchrotron Radiation Research Center (NSRRC), Hsinchu 300092, Taiwan

[#] Japan Synchrotron Radiation Research Institute (JASRI/SPring-8), Hyogo 6795198, Japan

E-mail: wfliaw@mx.nthu.edu.tw; ijuihsu@ntut.edu.tw; fttsai@mail.ncyu.edu.tw

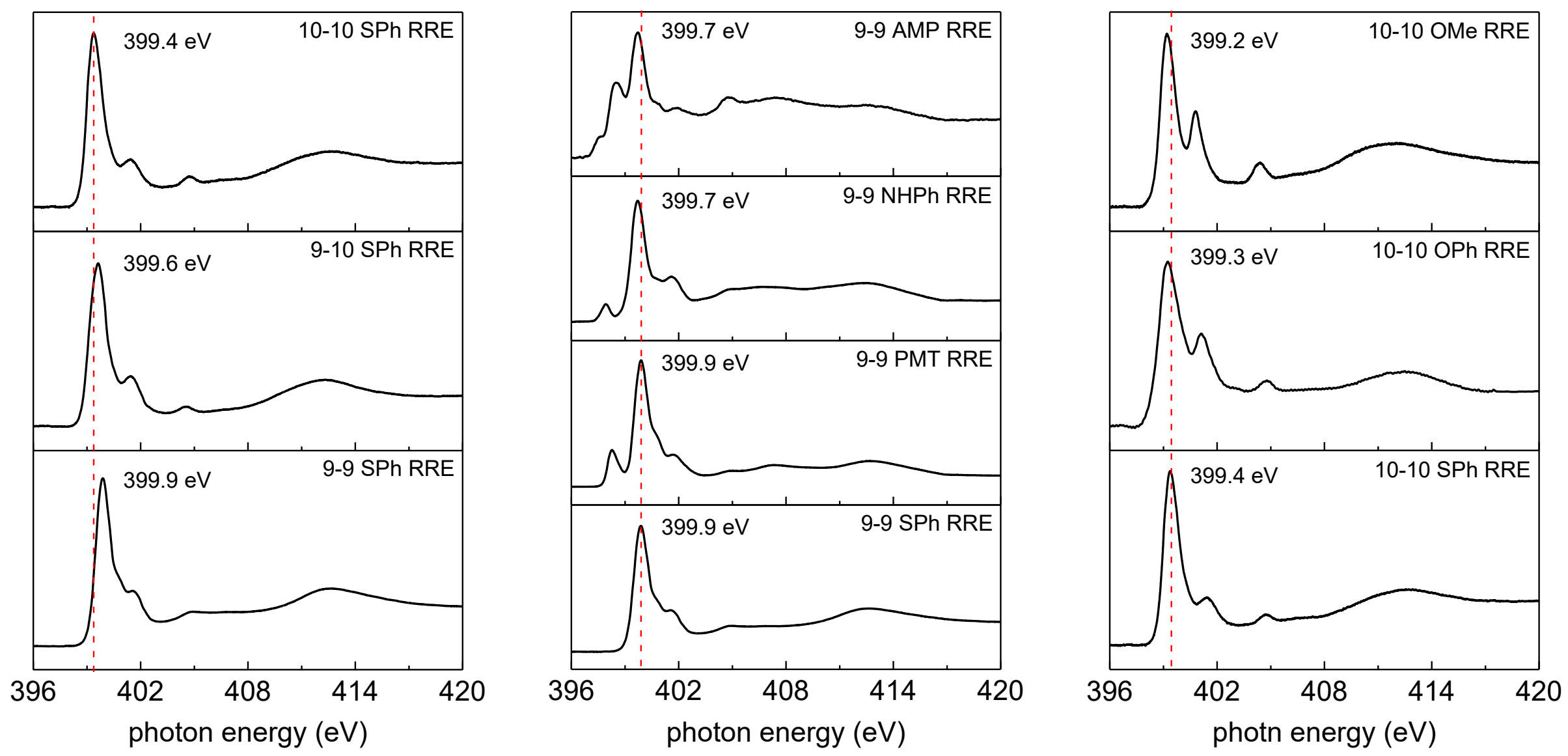

Figure S1. N K-edge XAS spectra for a series of $\{Fe(NO)_2\}^{9/10}$-$\{Fe(NO)_2\}^{9/10}$ RREs.

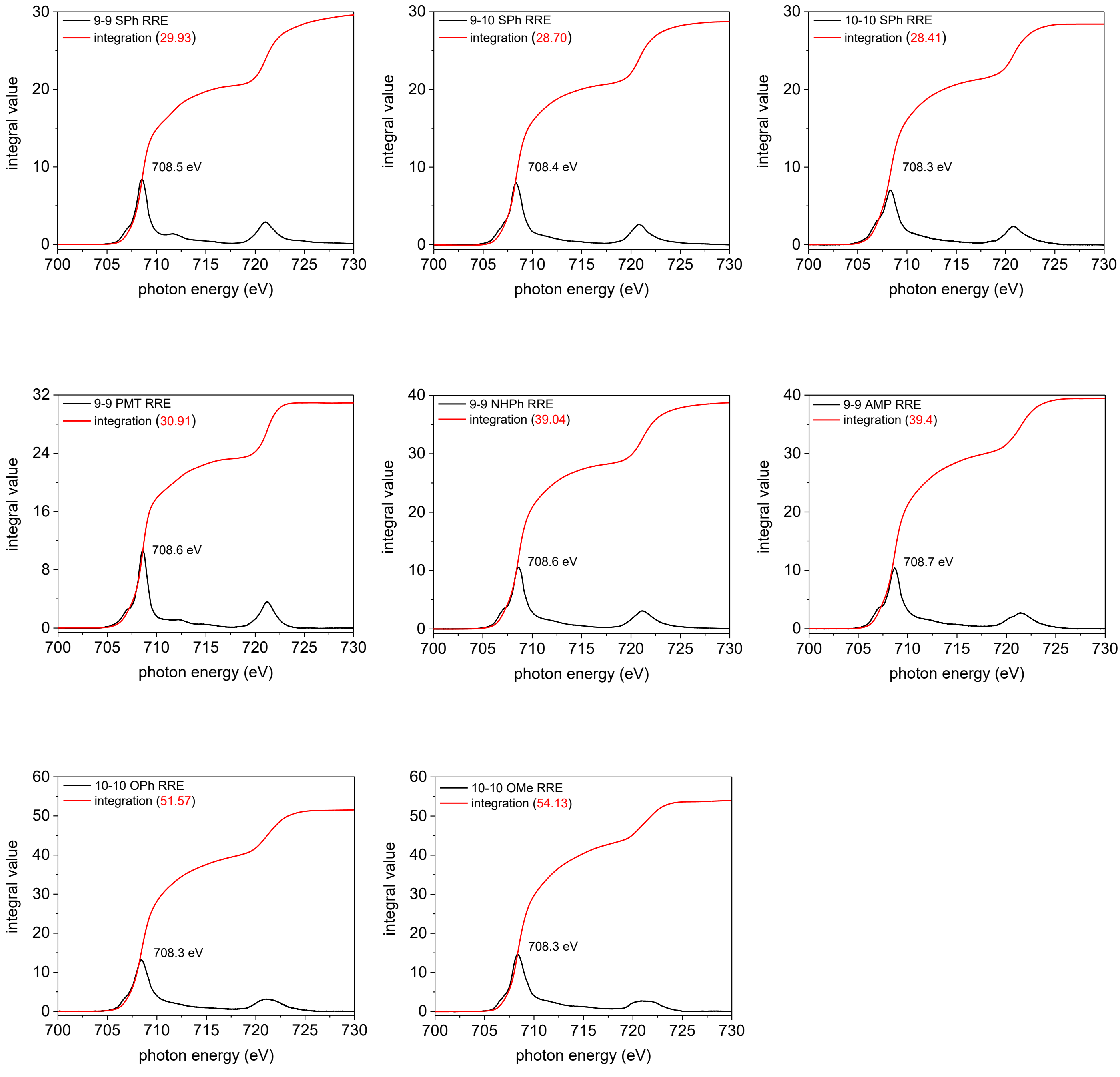

Figure S2. Fe L-edge XAS spectra and the corresponding integrated intensity for a series of $\{Fe(NO)_2\}^{9/10}$-$\{Fe(NO)_2\}^{9/10}$ RREs.

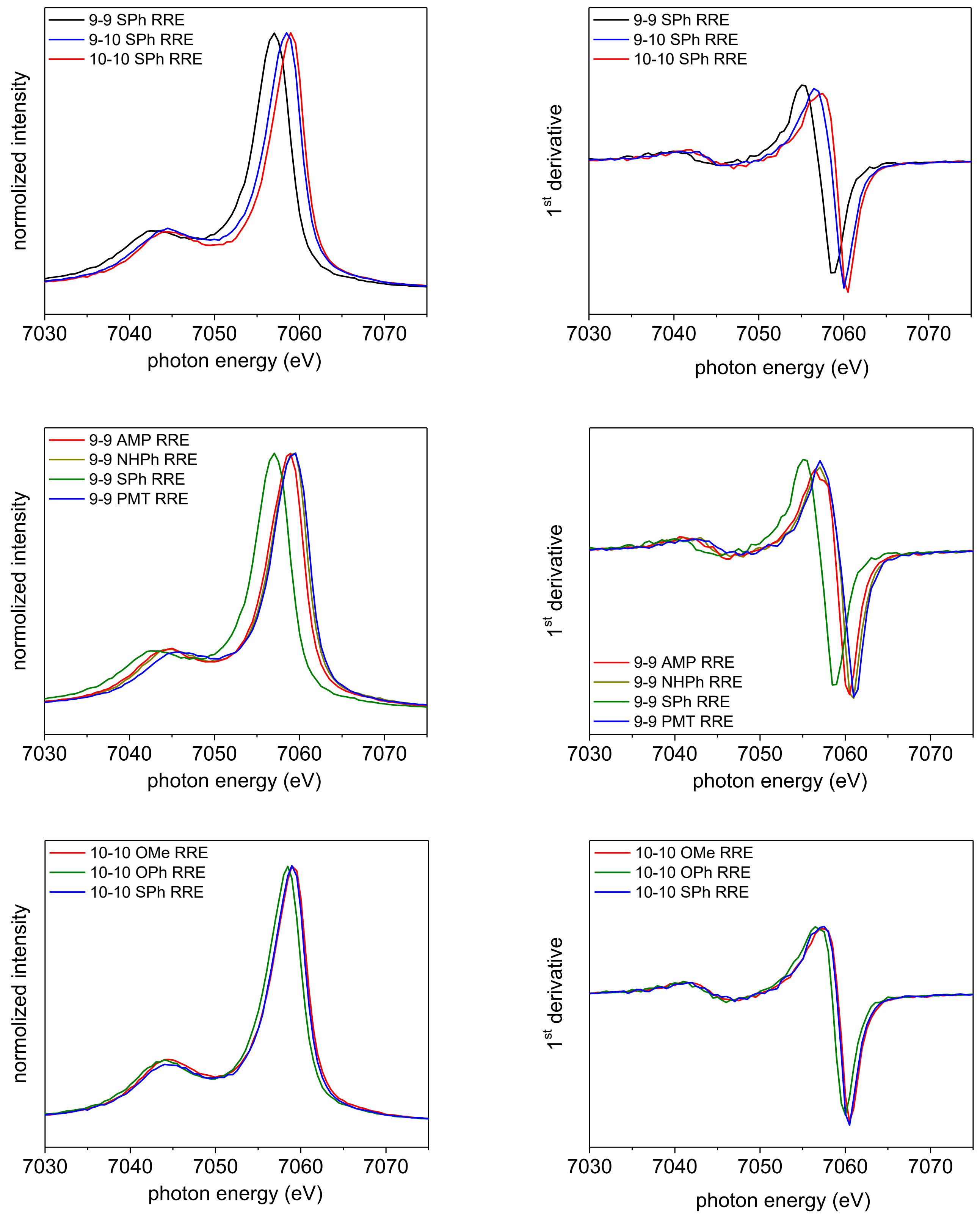

Figure S3. Fe K-edge Kβ$_{1,3}$ mainline XES spectra and the corresponding first derivative for a series of $\{Fe(NO)_2\}^{9/10}$-$\{Fe(NO)_2\}^{9/10}$ RREs.

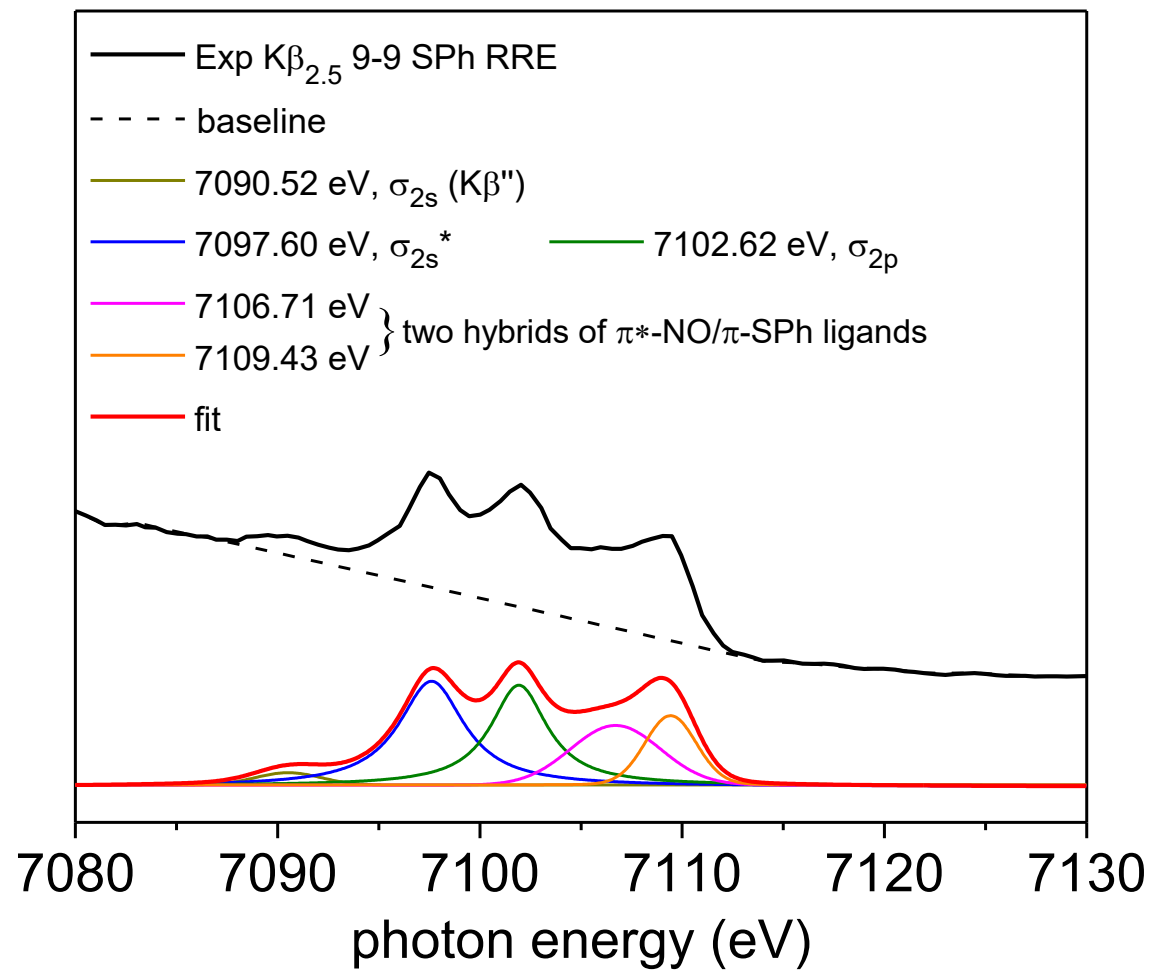
Exp $K\beta_{2,5}$ 9-9 SPh RRE
baseline
7090.52 eV, σ_{2s} (Kβ'')
7097.60 eV, σ_{2s}*
7102.62 eV, σ_{2p}
7106.71 eV
7109.43 eV
two hybrids of π*-NO/π-SPh ligands
fit
7080 7090 7100 7110 7120 7130
photon energy (eV)

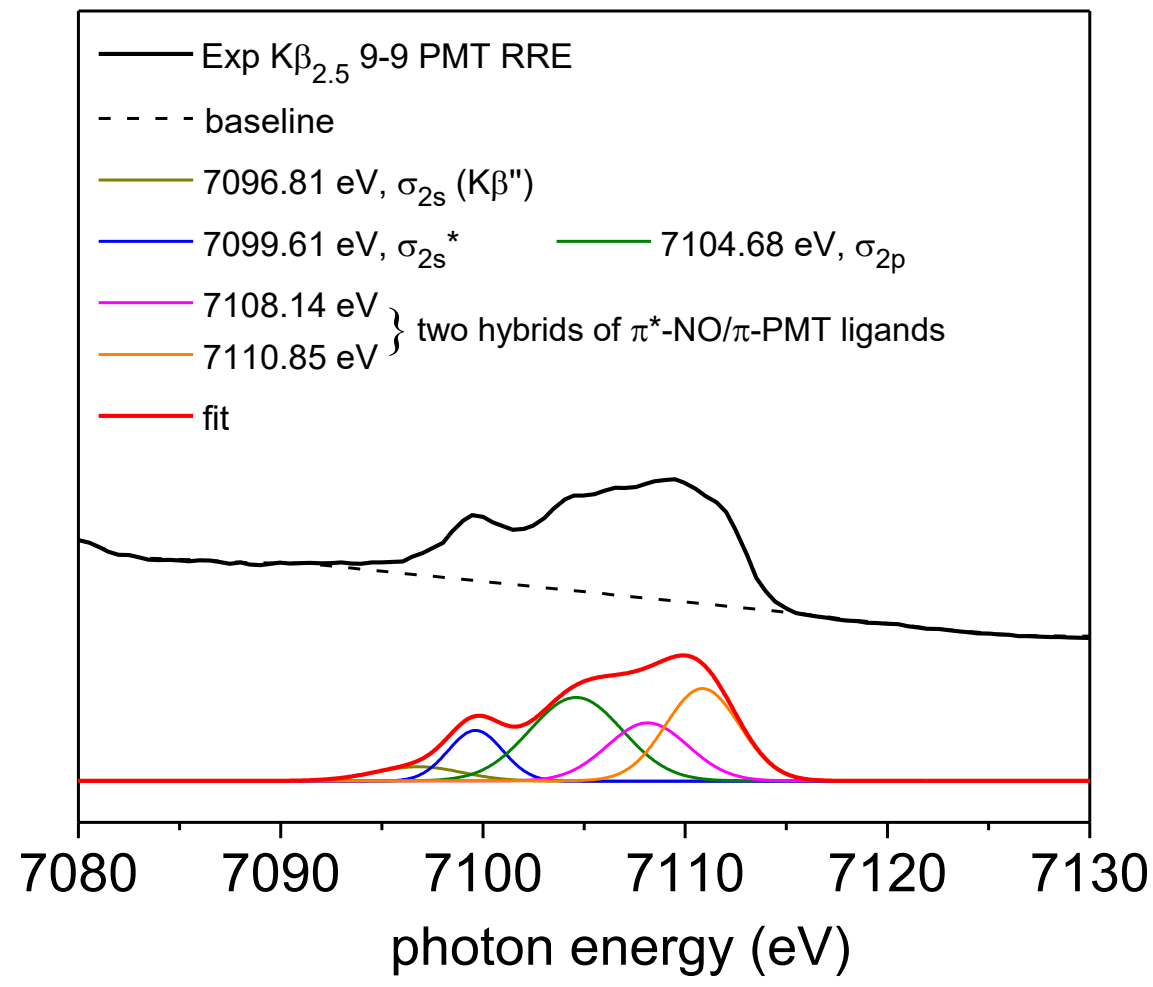
Exp $K\beta_{2,5}$ 9-9 PMT RRE
baseline
7096.81 eV, σ_{2s} (Kβ'')
7099.61 eV, σ_{2s}*
7104.68 eV, σ_{2p}
7108.14 eV
7110.85 eV
two hybrids of π*-NO/π-PMT ligands
fit
7080 7090 7100 7110 7120 7130
photon energy (eV)

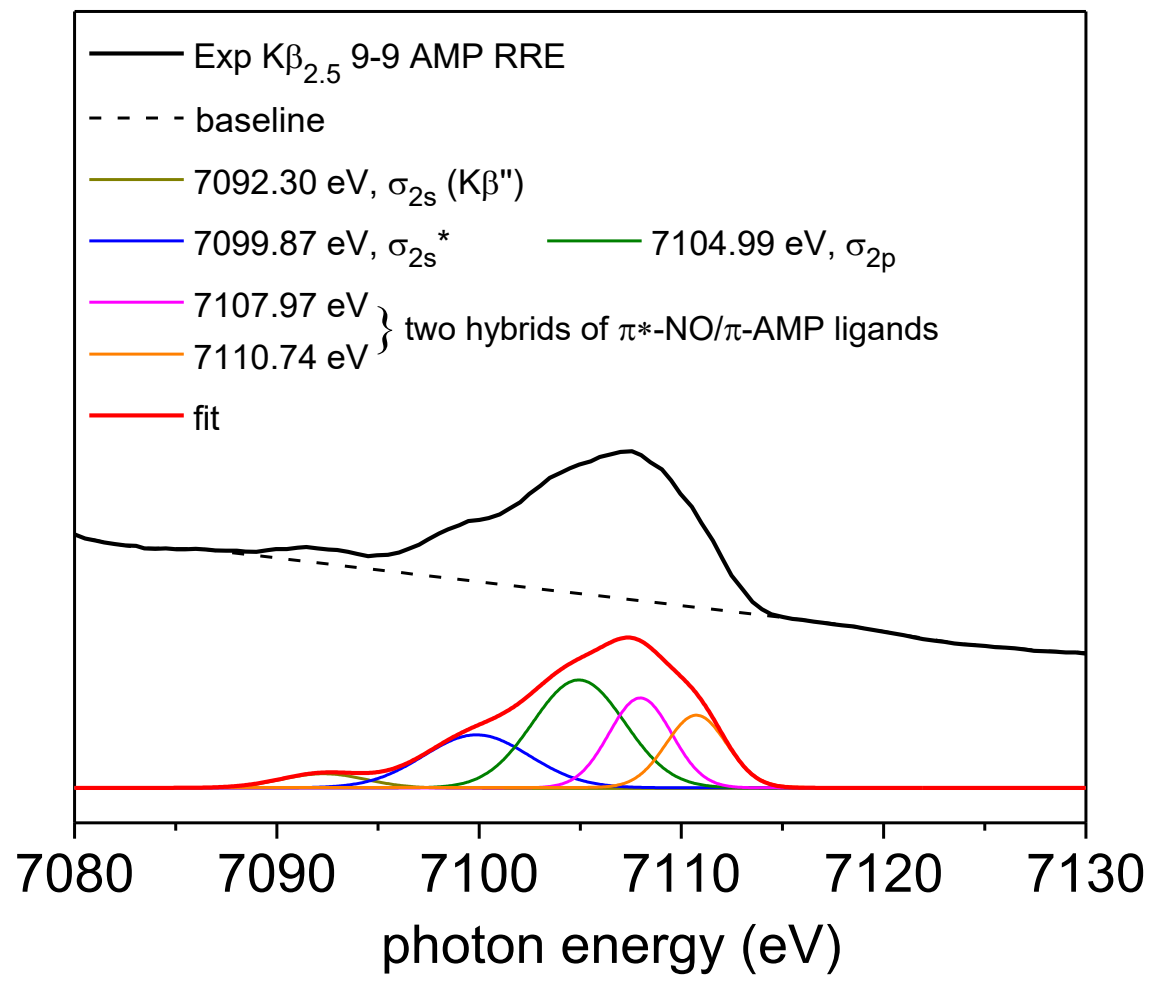
Exp $K\beta_{2,5}$ 9-9 AMP RRE
baseline
7092.30 eV, σ_{2s} (Kβ'')
7099.87 eV, σ_{2s}*
7104.99 eV, σ_{2p}
7107.97 eV
7110.74 eV
two hybrids of π*-NO/π-AMP ligands
fit
7080 7090 7100 7110 7120 7130
photon energy (eV)

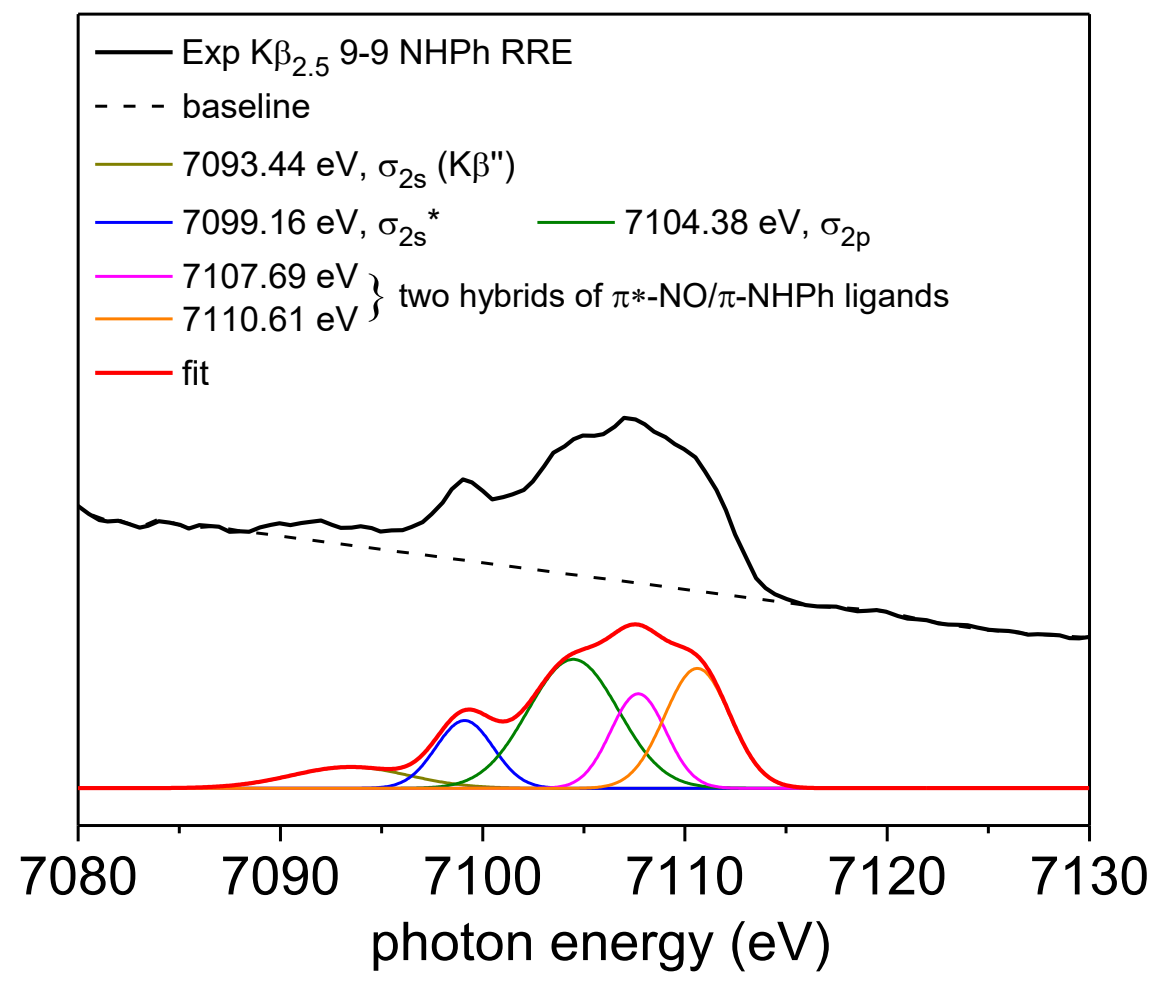
Exp $K\beta_{2,5}$ 9-9 NHPh RRE
baseline
7093.44 eV, σ_{2s} (Kβ'')
7099.16 eV, σ_{2s}*
7104.38 eV, σ_{2p}
7107.69 eV
7110.61 eV
two hybrids of π*-NO/π-NHPh ligands
fit
7080 7090 7100 7110 7120 7130
photon energy (eV)

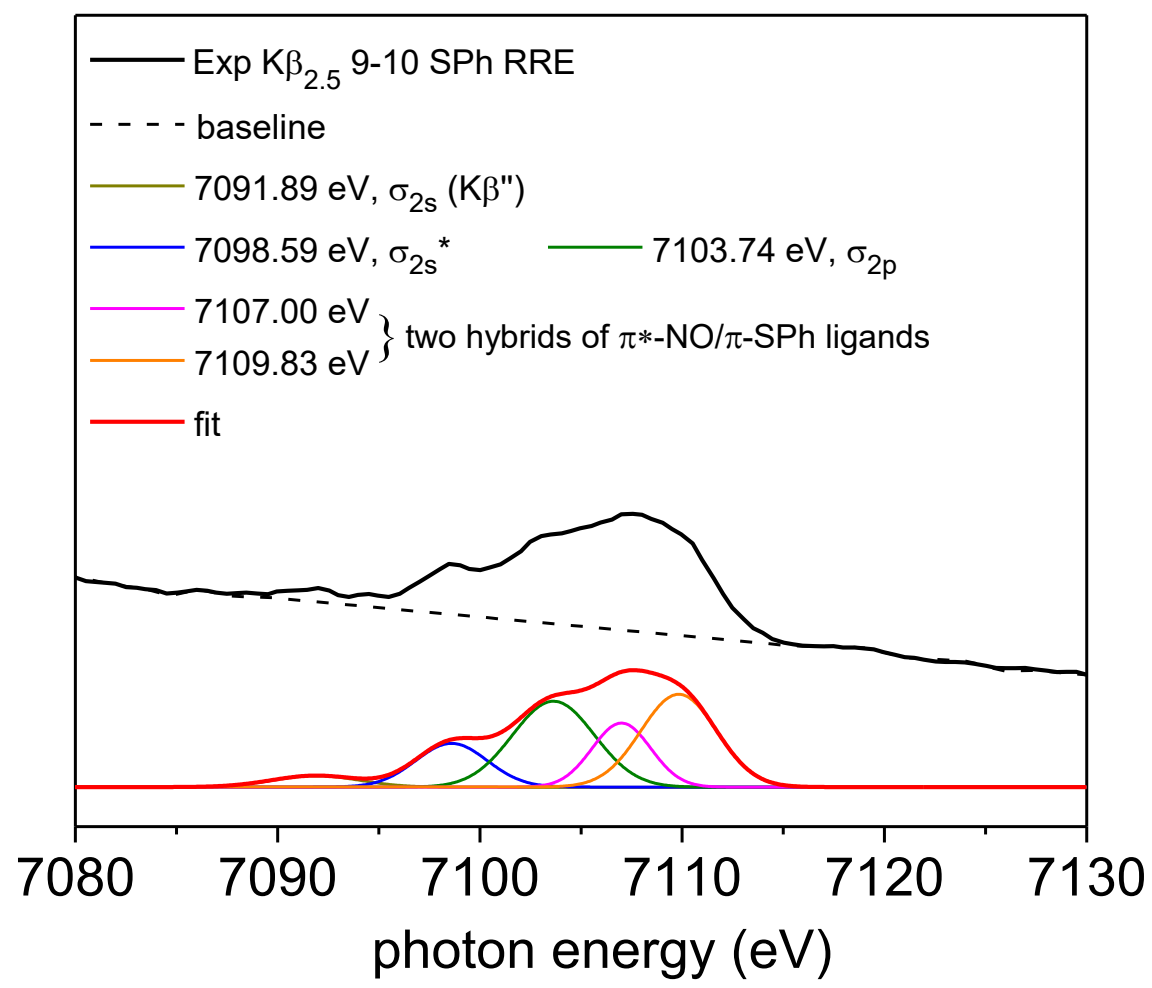
Exp $K\beta_{2,5}$ 9-10 SPh RRE
baseline
7091.89 eV, σ_{2s} (Kβ'')
7098.59 eV, σ_{2s}*
7103.74 eV, σ_{2p}
7107.00 eV
7109.83 eV
two hybrids of π*-NO/π-SPh ligands
fit
7080 7090 7100 7110 7120 7130
photon energy (eV)

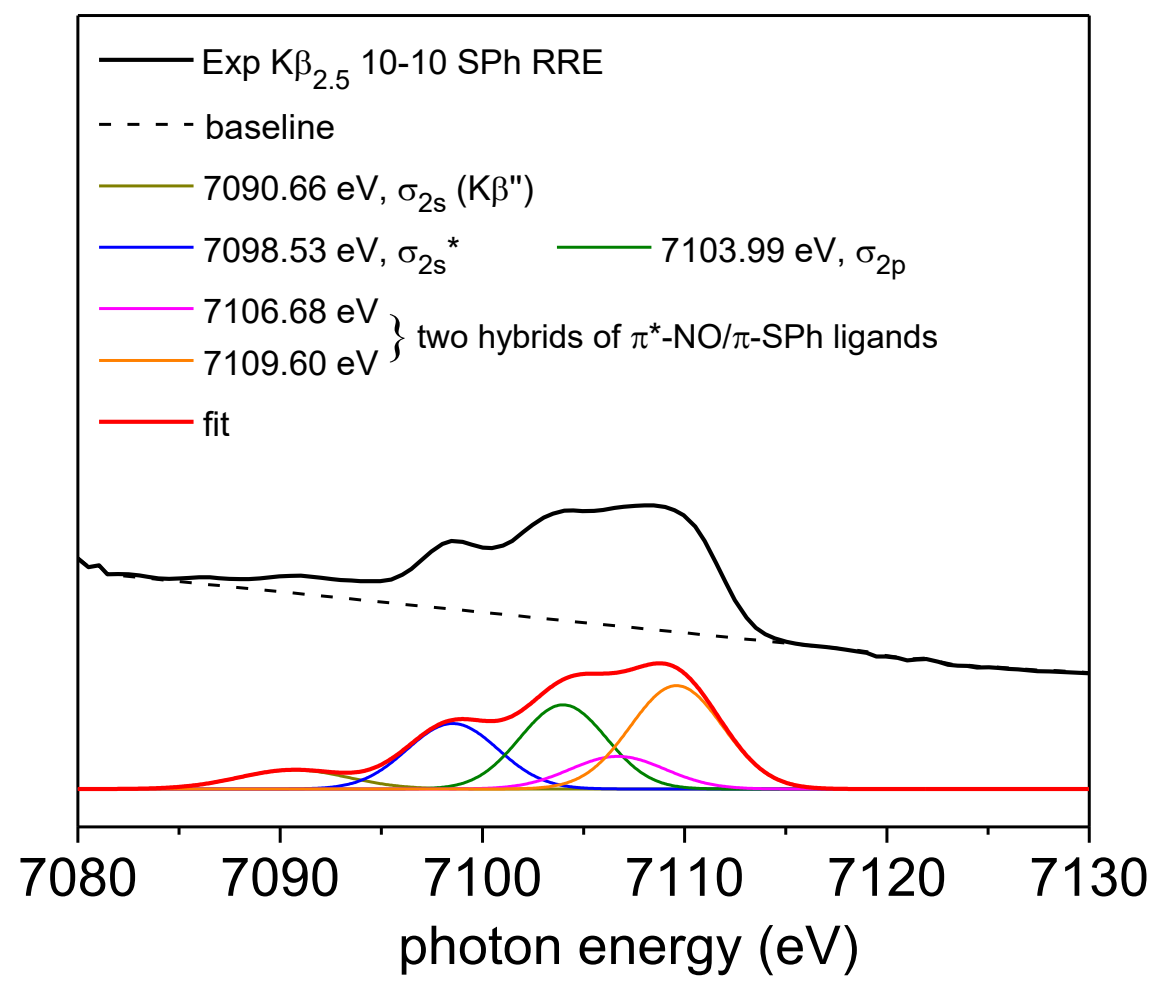
Exp $K\beta_{2,5}$ 10-10 SPh RRE
baseline
7090.66 eV, σ_{2s} (Kβ'')
7098.53 eV, σ_{2s}*
7103.99 eV, σ_{2p}
7106.68 eV
7109.60 eV
two hybrids of π*-NO/π-SPh ligands
fit
7080 7090 7100 7110 7120 7130
photon energy (eV)

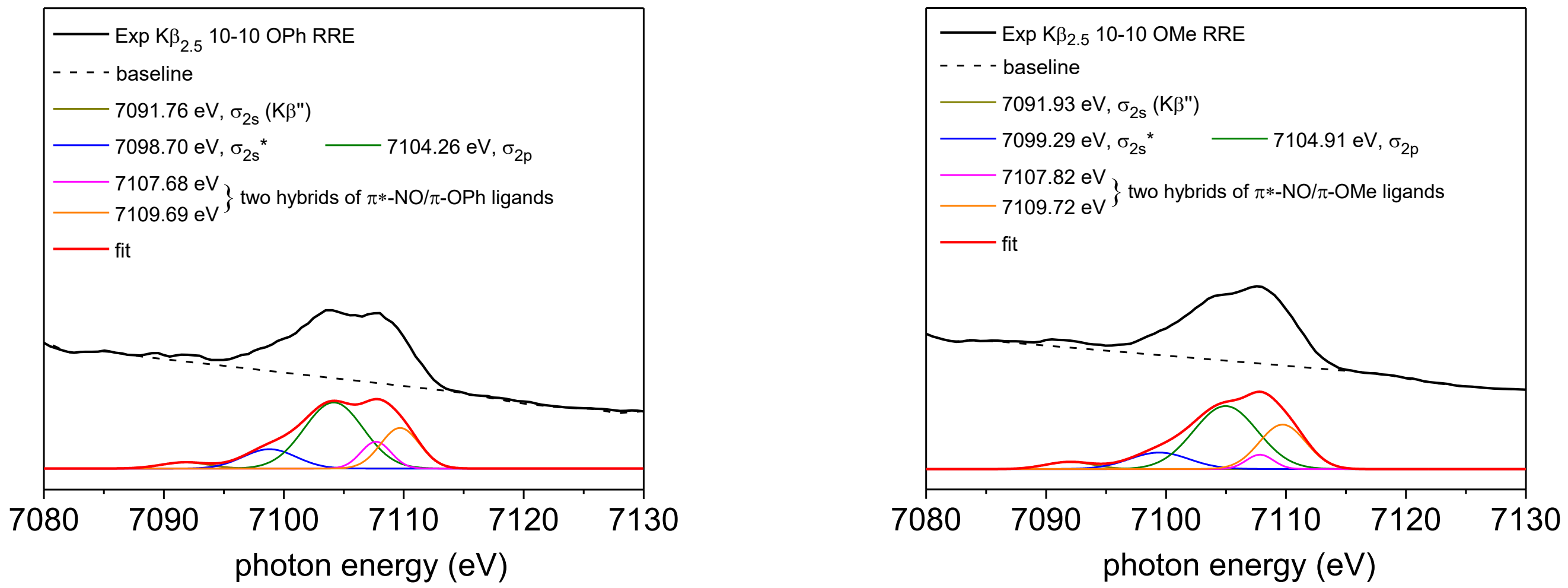

Figure S4. Fe K-edge VtC K$\beta_{2,5}$ XES spectra and the corresponding fitting results for a series of $\{Fe(NO)_2\}^{9/10}$-$\{Fe(NO)_2\}^{9/10}$ RREs

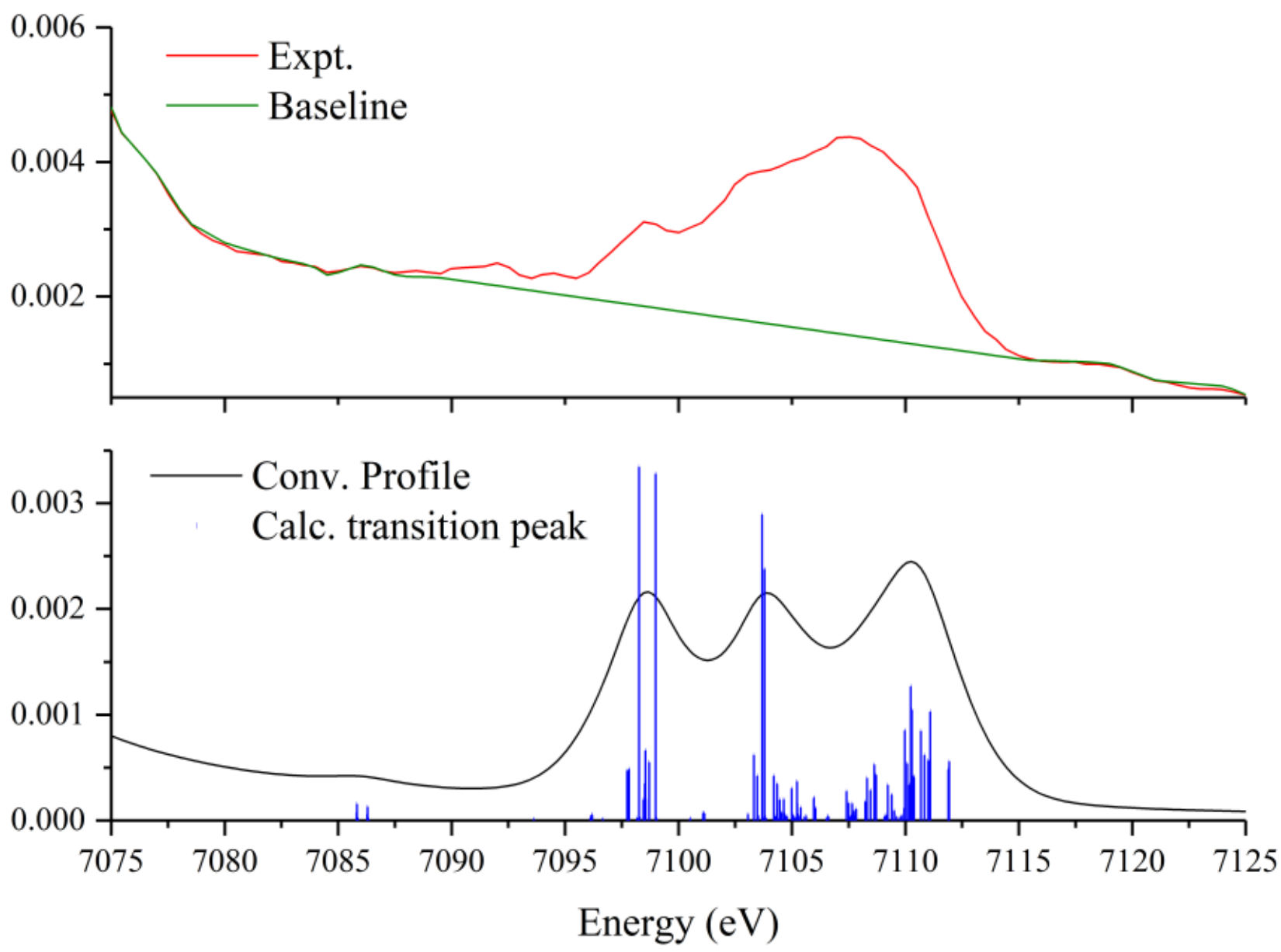

Figure S5. Experimental and calculated VtC K$\beta_{2,5}$ XES of **9-10 SPh RRE**. The blue lines represent the calculated transitions, while the black line is the convoluted spectrum using a Voigt profile with a 3 eV Lorentzian and 5 eV Gaussian linewidth.

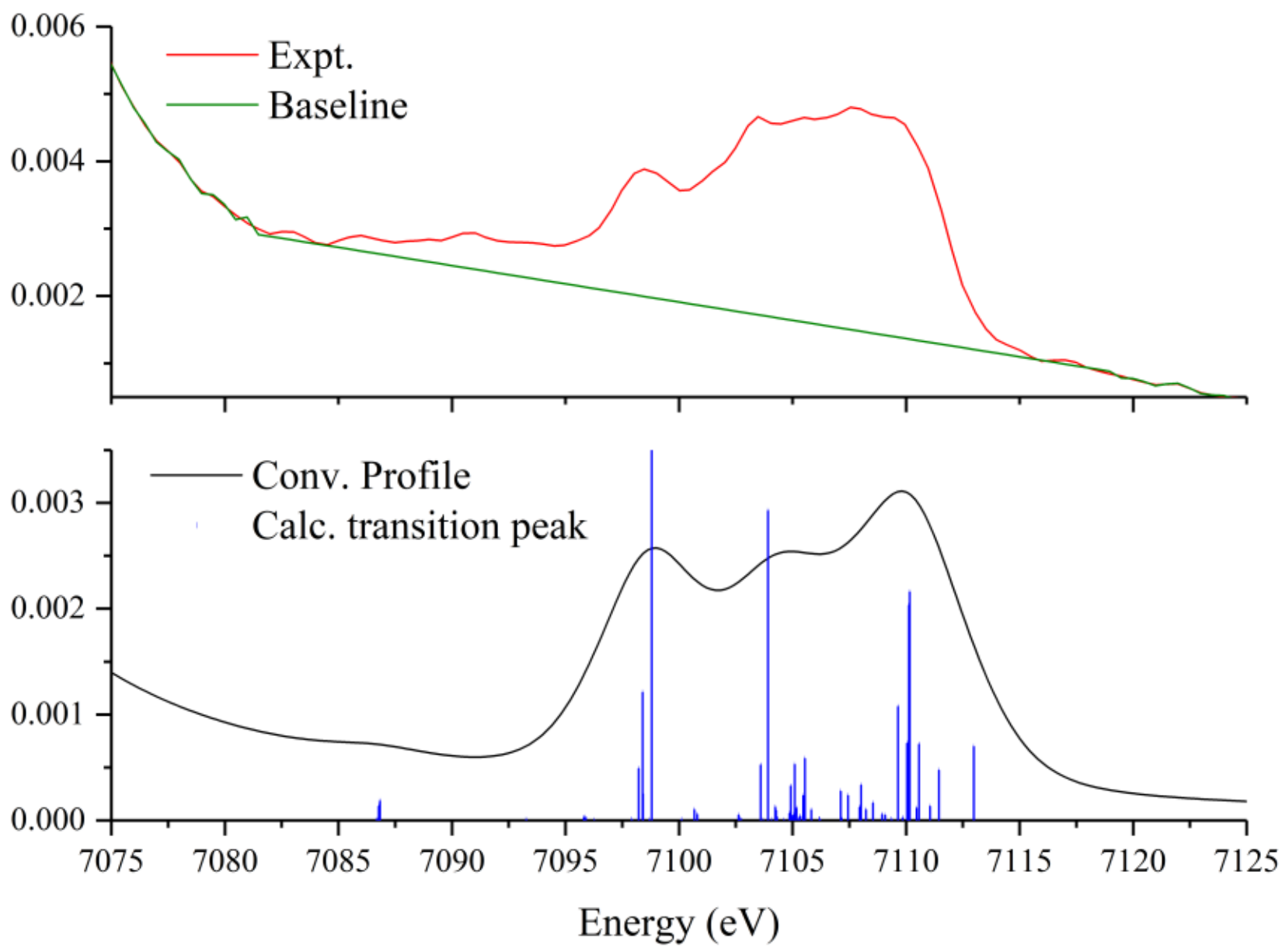

Figure S6. Experimental and calculated VtC K$\beta_{2,5}$ XES of **10-10 SPh RRE**. The blue lines represent the calculated transitions, while the black line is the convoluted spectrum using a Voigt profile with a 5 eV Lorentzian and 5.5 eV Gaussian linewidth.

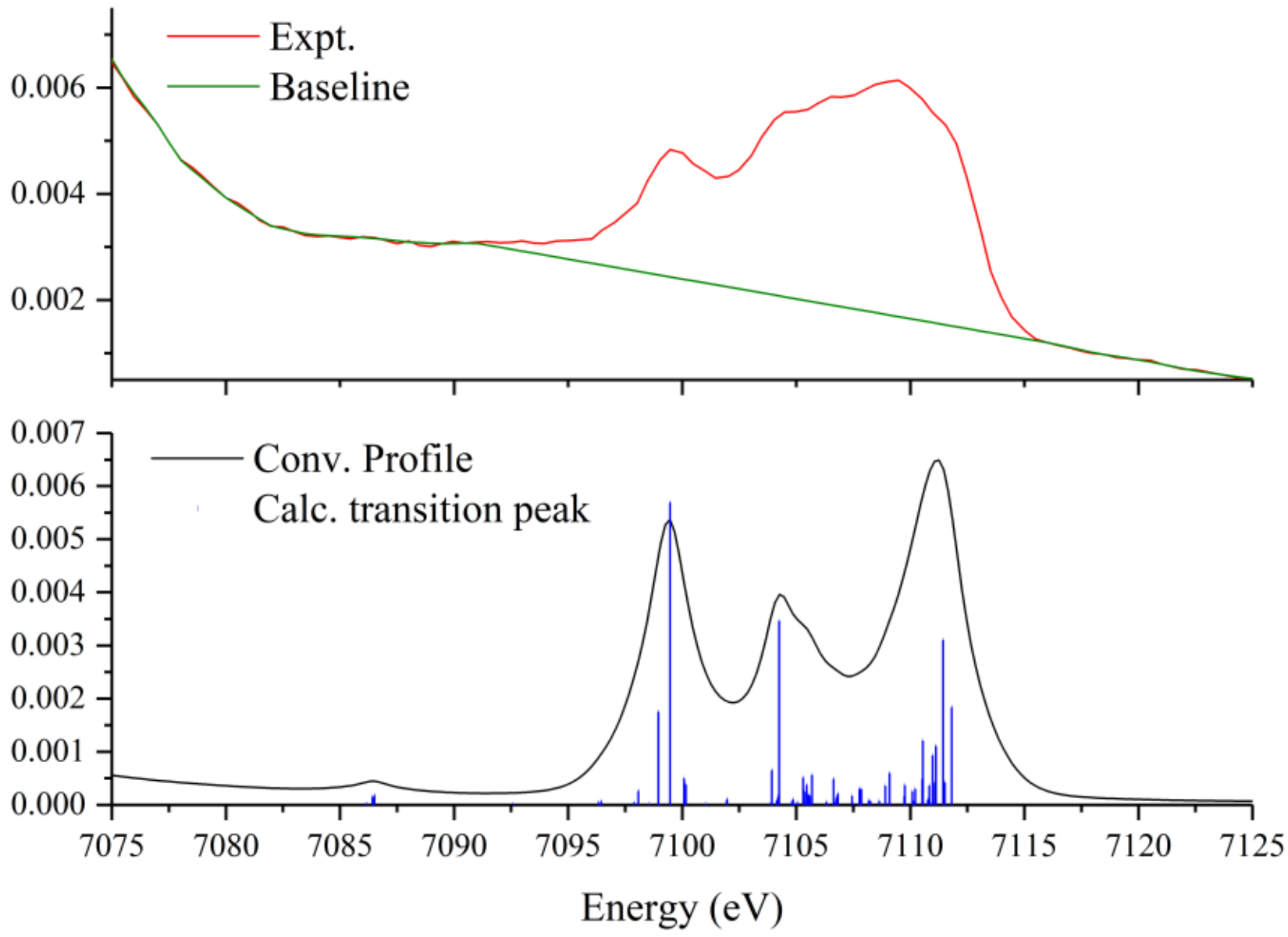

Figure S7. Experimental and calculated VtC K$\beta_{2,5}$ XES of **9-9 PMT RRE**. The blue lines represent the calculated transitions, while the black line is the convoluted spectrum using a Voigt profile with a 1.5 eV Lorentzian and 4 eV Gaussian linewidth.

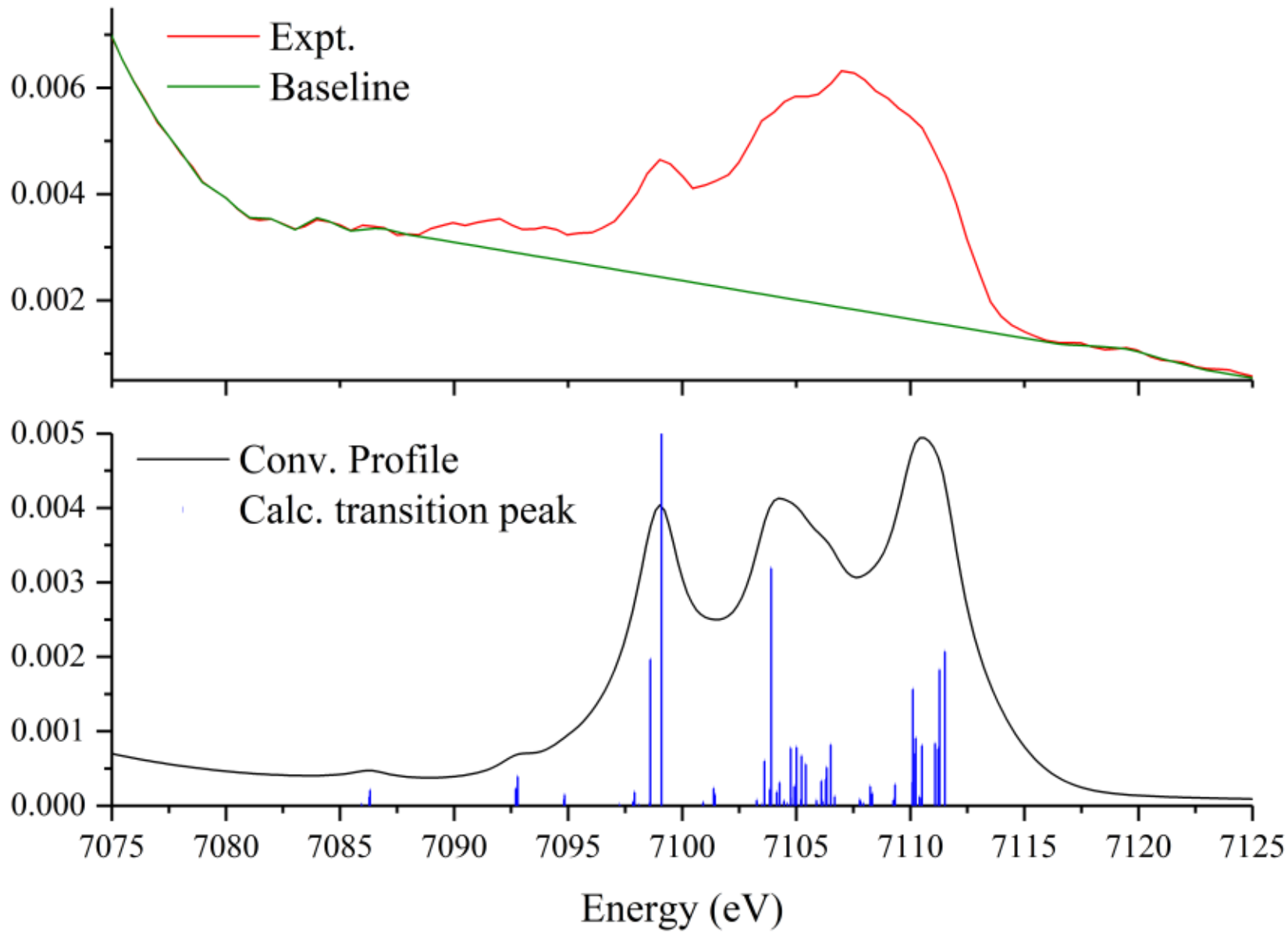

Figure S8. Experimental and calculated VtC K$\beta_{2,5}$ XES of **9-9 NHPh RRE**. The blue lines represent the calculated transitions, while the black line is the convoluted spectrum using a Voigt profile with a 2 eV Lorentzian and 6 eV Gaussian linewidth.

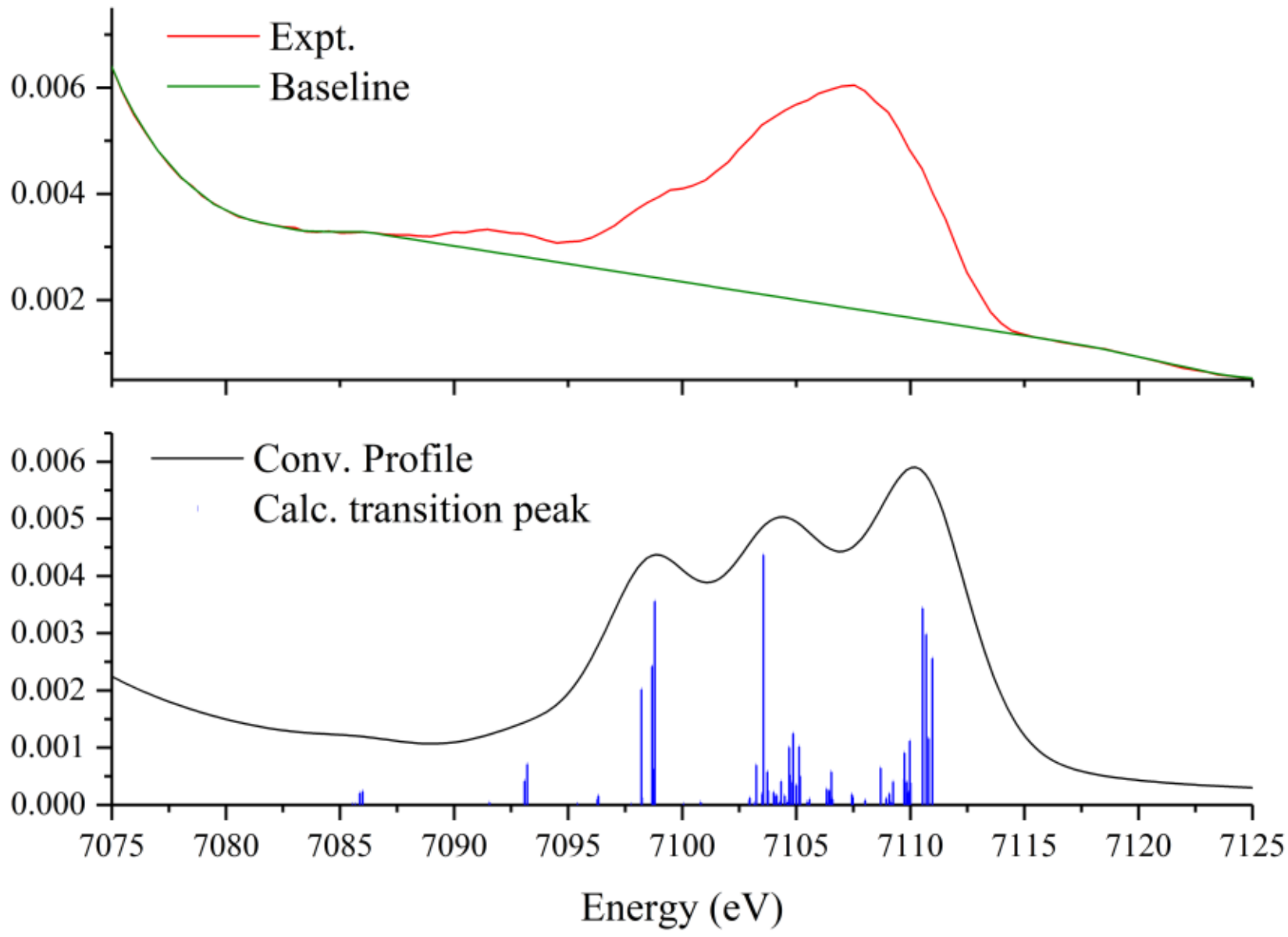

Figure S9. Experimental and calculated VtC K$\beta_{2,5}$ XES of **9-9 AMP RRE**. The blue lines represent the calculated transitions, while the black line is the convoluted spectrum using a Voigt profile with a 5 eV Lorentzian and 10 eV Gaussian linewidth.

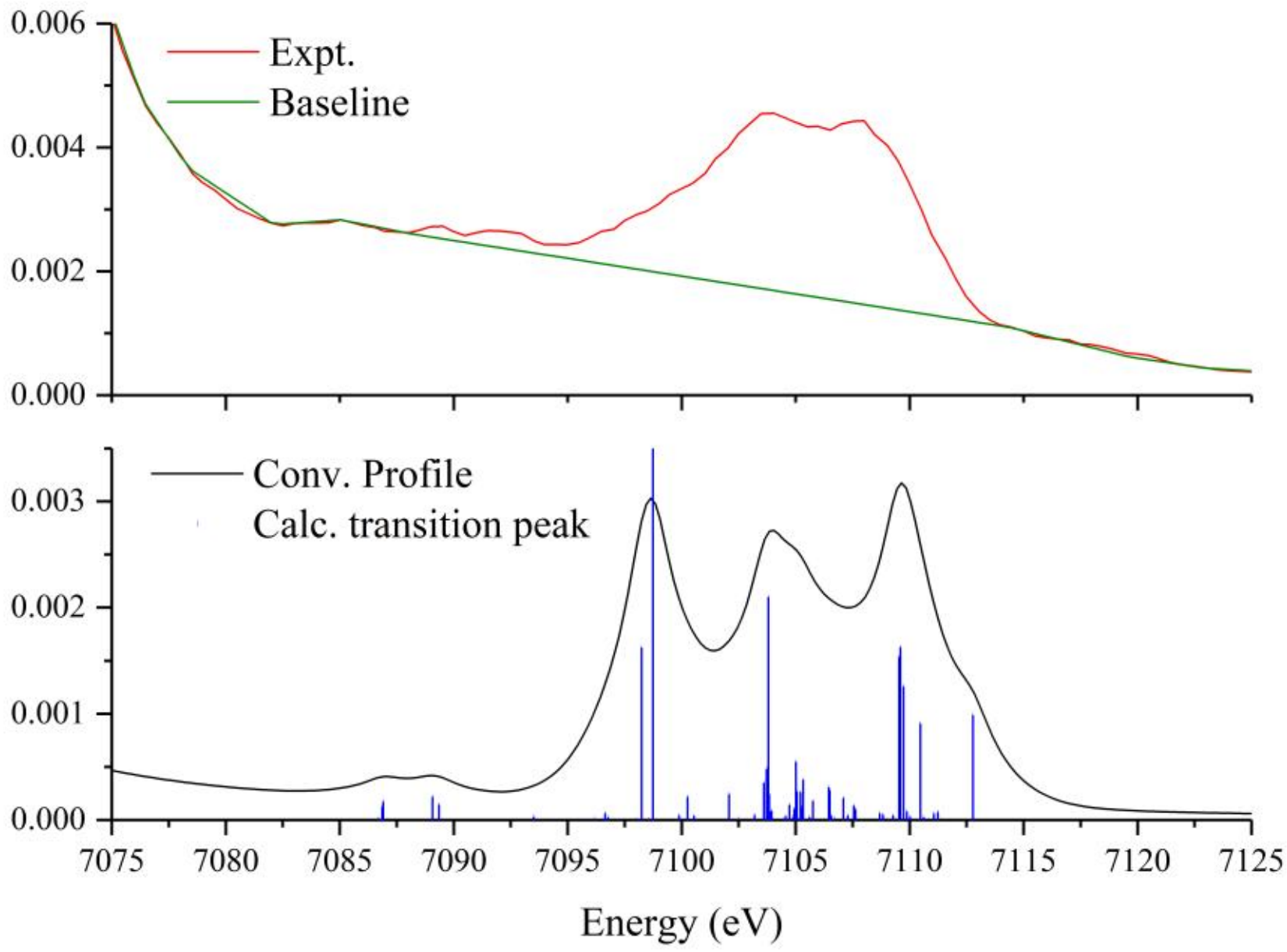

Figure S10. Experimental and calculated VtC K$\beta_{2,5}$ XES of **10-10 OPh RRE**. The blue lines represent the calculated transitions, while the black line is the convoluted spectrum using a Voigt profile with a 2 eV Lorentzian and 5 eV Gaussian linewidth.

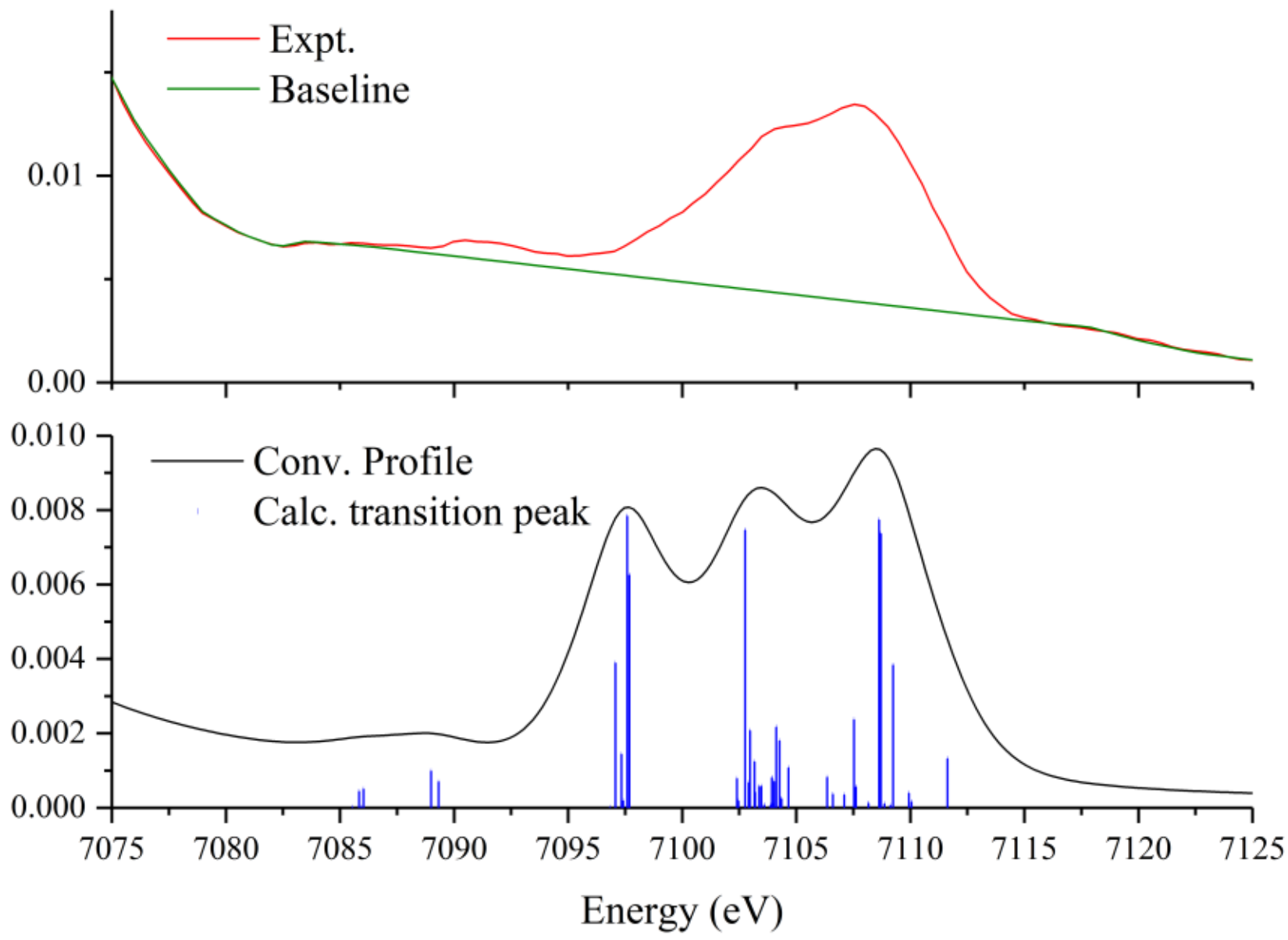

Figure S11. Experimental and calculated VtC K$\beta_{2,5}$ XES of **10-10 OMe RRE**. The blue lines represent the calculated transitions, while the black line is the convoluted spectrum using a Voigt profile with a 4 eV Lorentzian and 5 eV Gaussian linewidth.

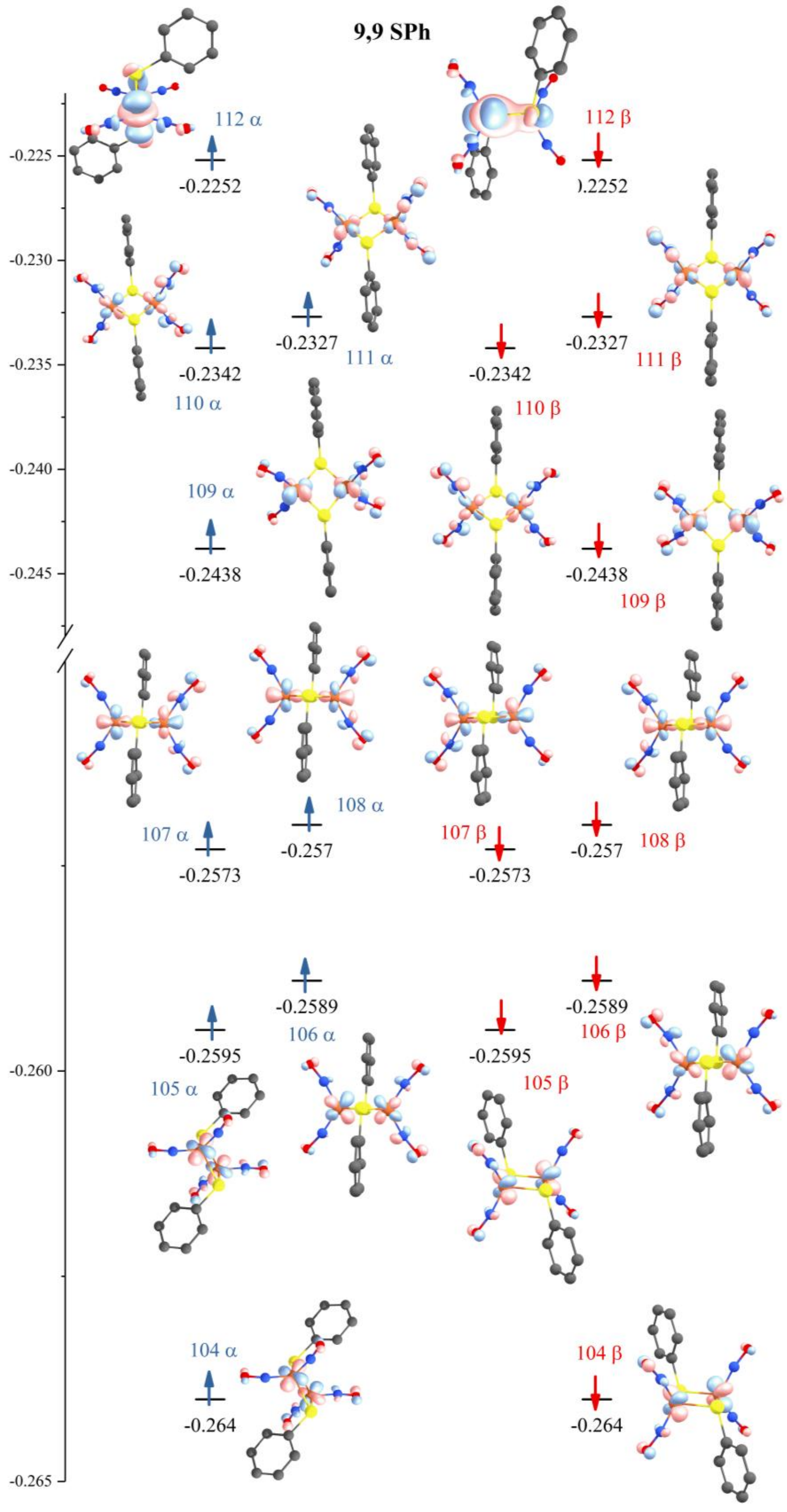

Figure S12. The corresponding orbitals of **9-9 SPh RRE**. The unit of orbital energy is Hartree.

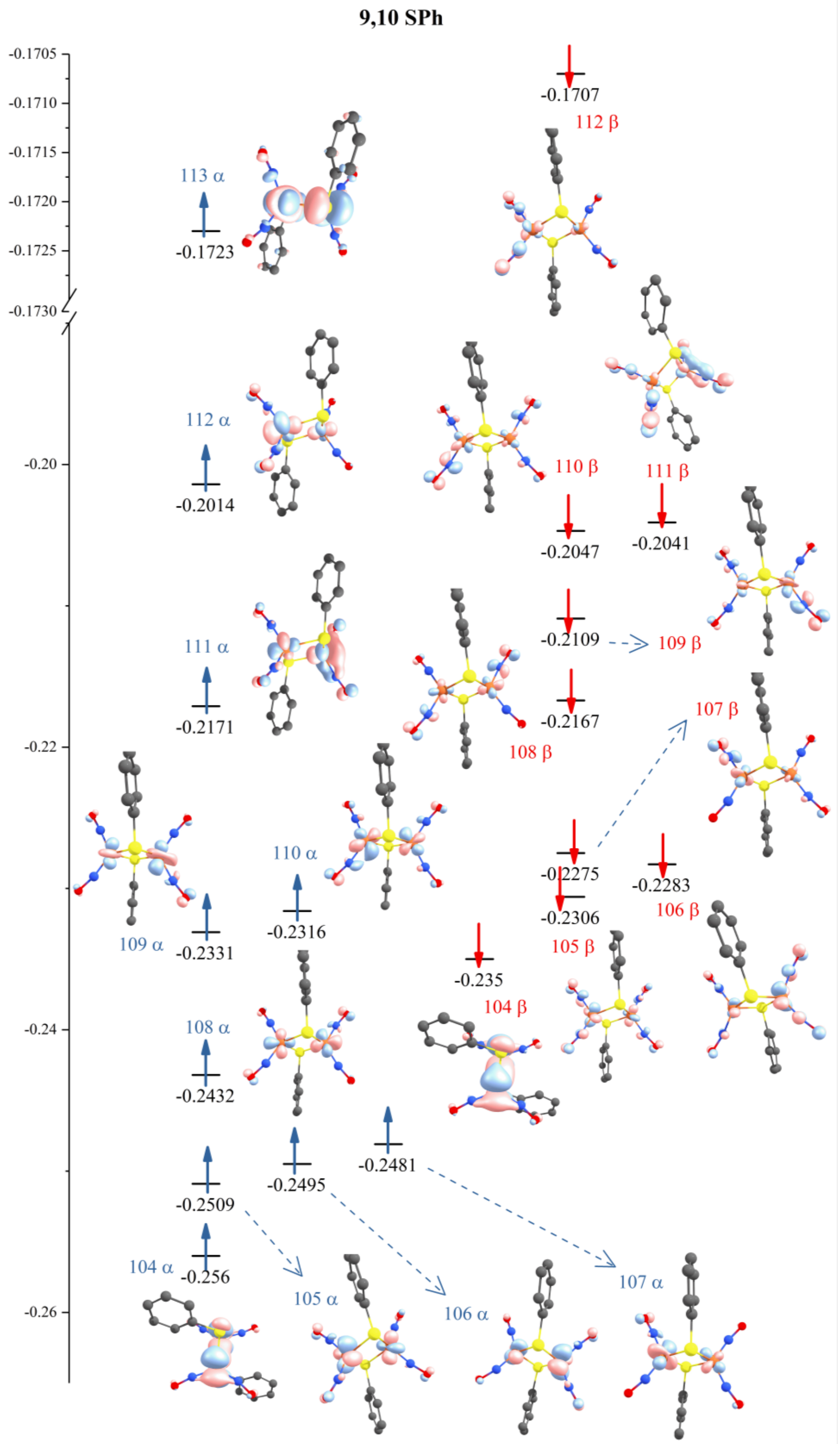

Figure S13. The corresponding orbitals of **9-10 SPh RRE**. The unit of orbital energy is Hartree.

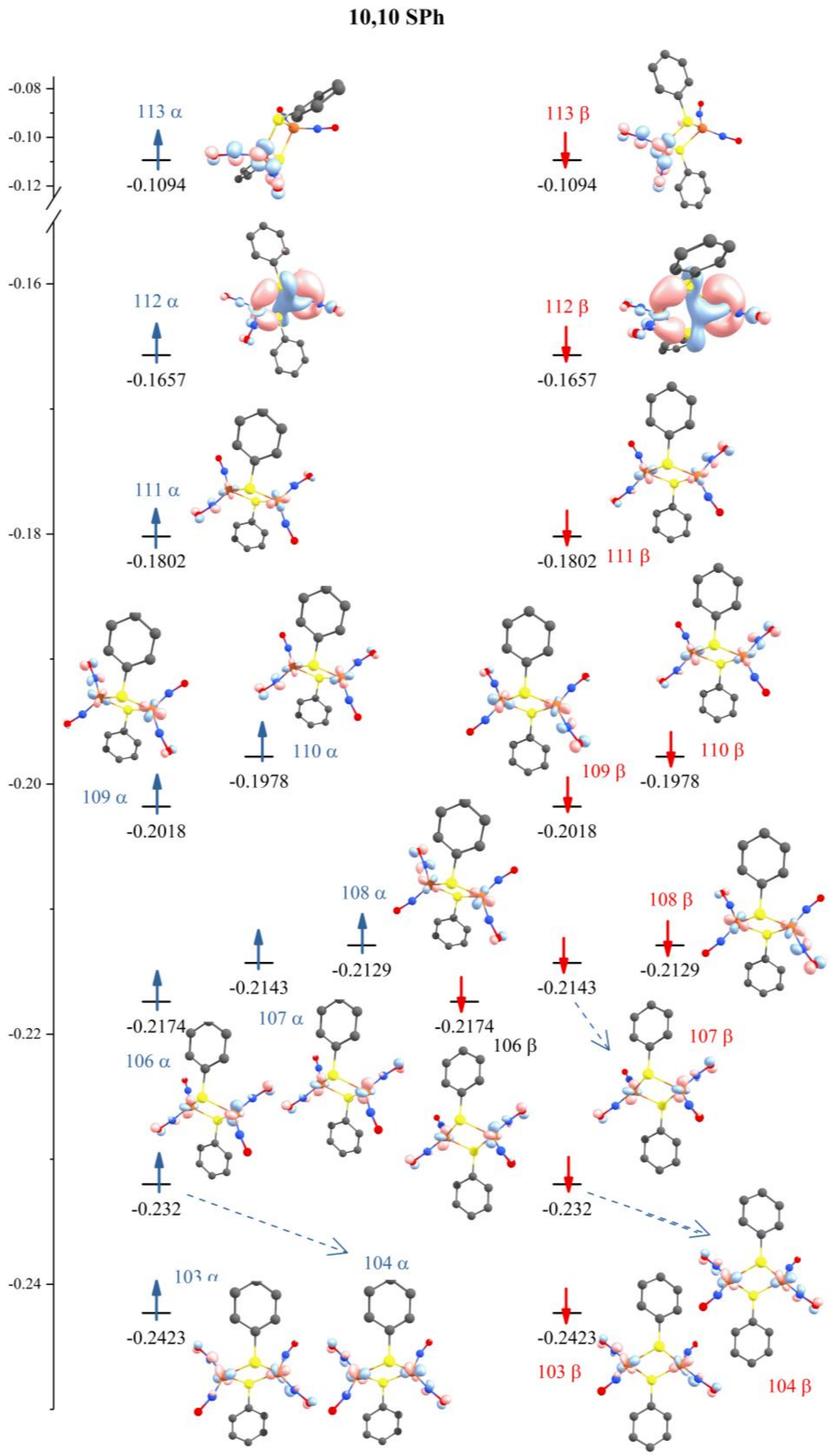

Figure S14. The corresponding orbitals of **10-10 SPh RRE**. The unit of orbital energy is Hartree.

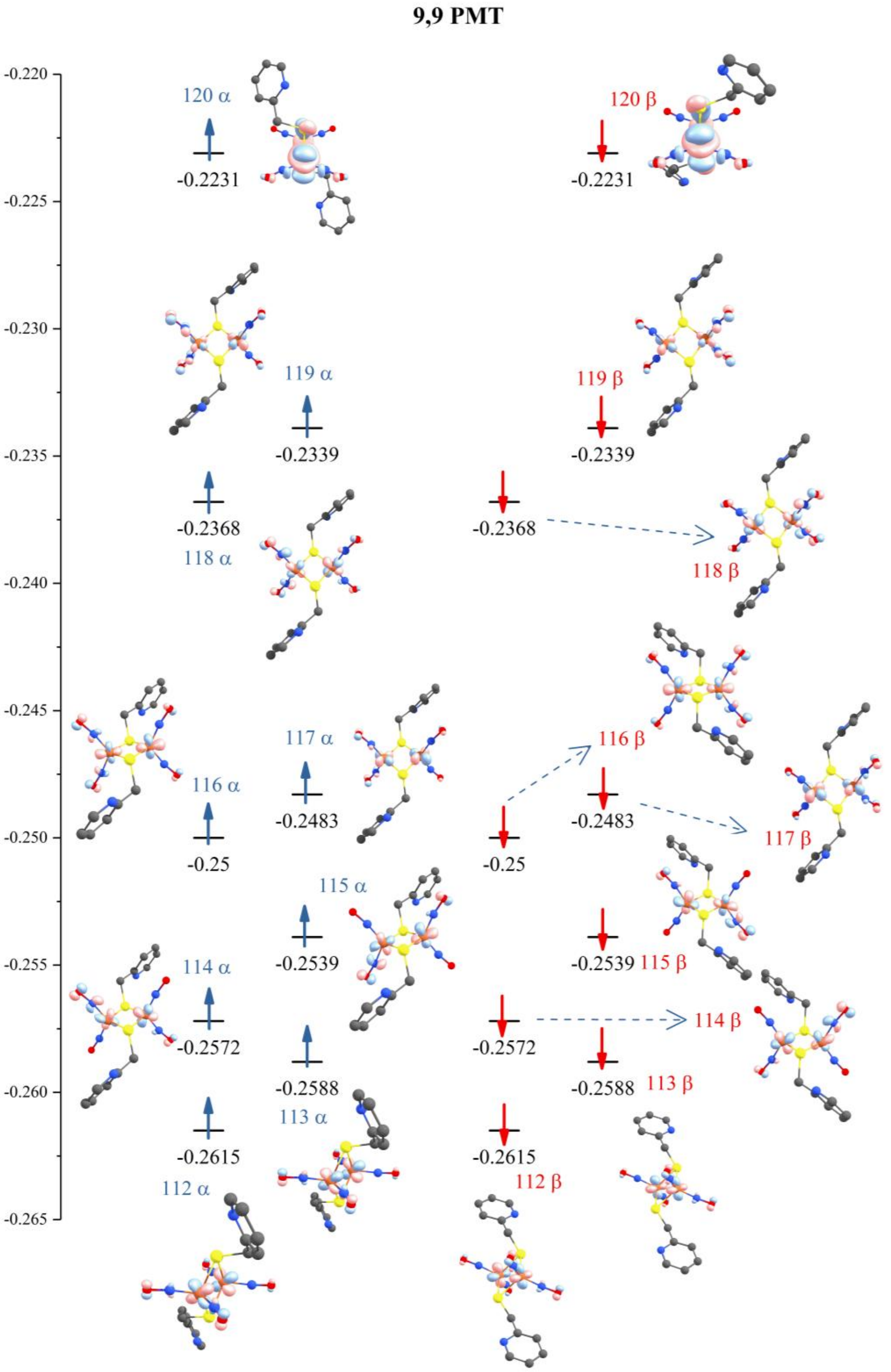

Figure S15. The corresponding orbitals of **9-9 PMT RRE**. The unit of orbital energy is Hartree.

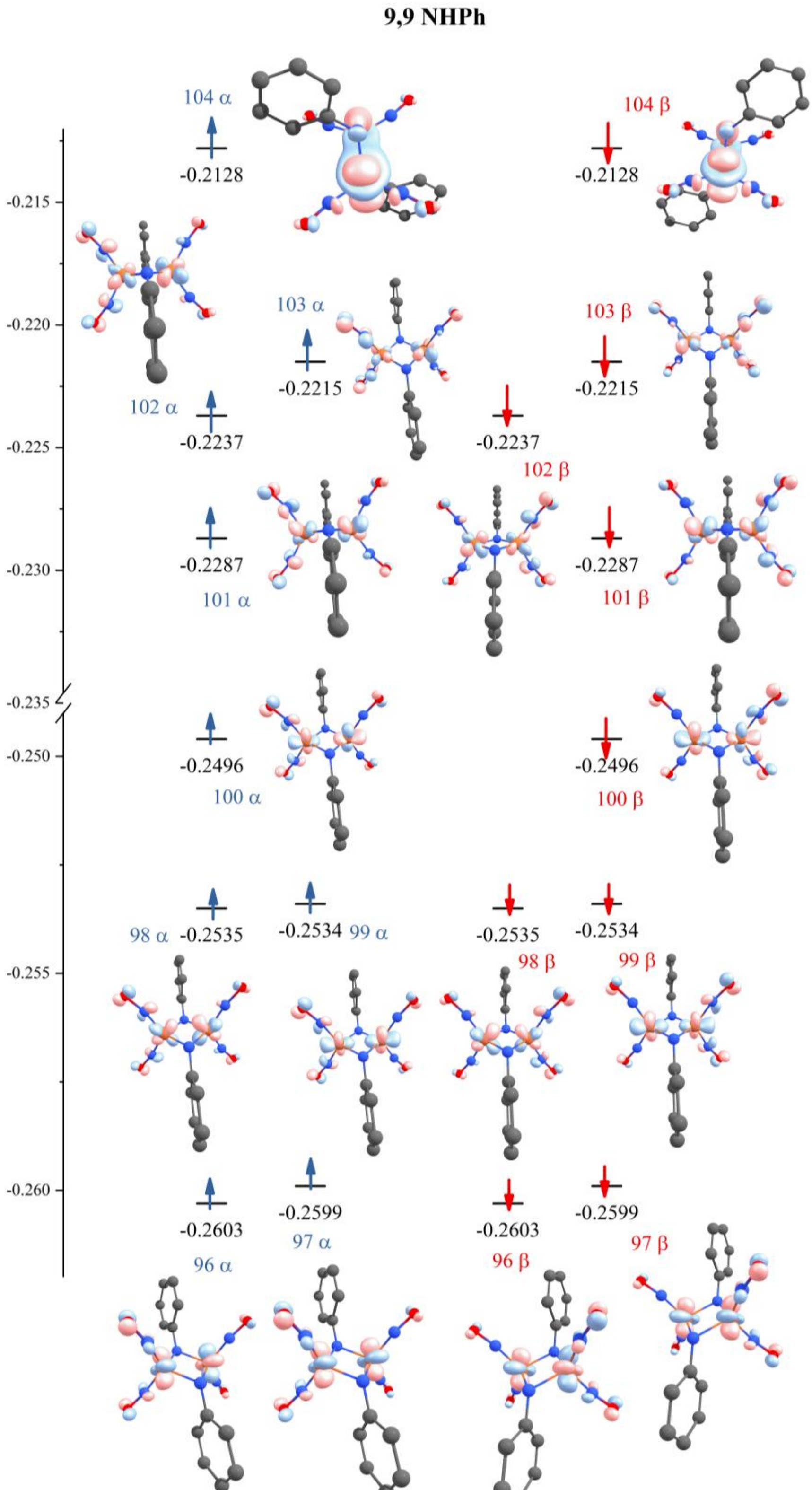

Figure S16. The corresponding orbitals of **9-9 NHPh RRE**. The unit of orbital energy is Hartree.

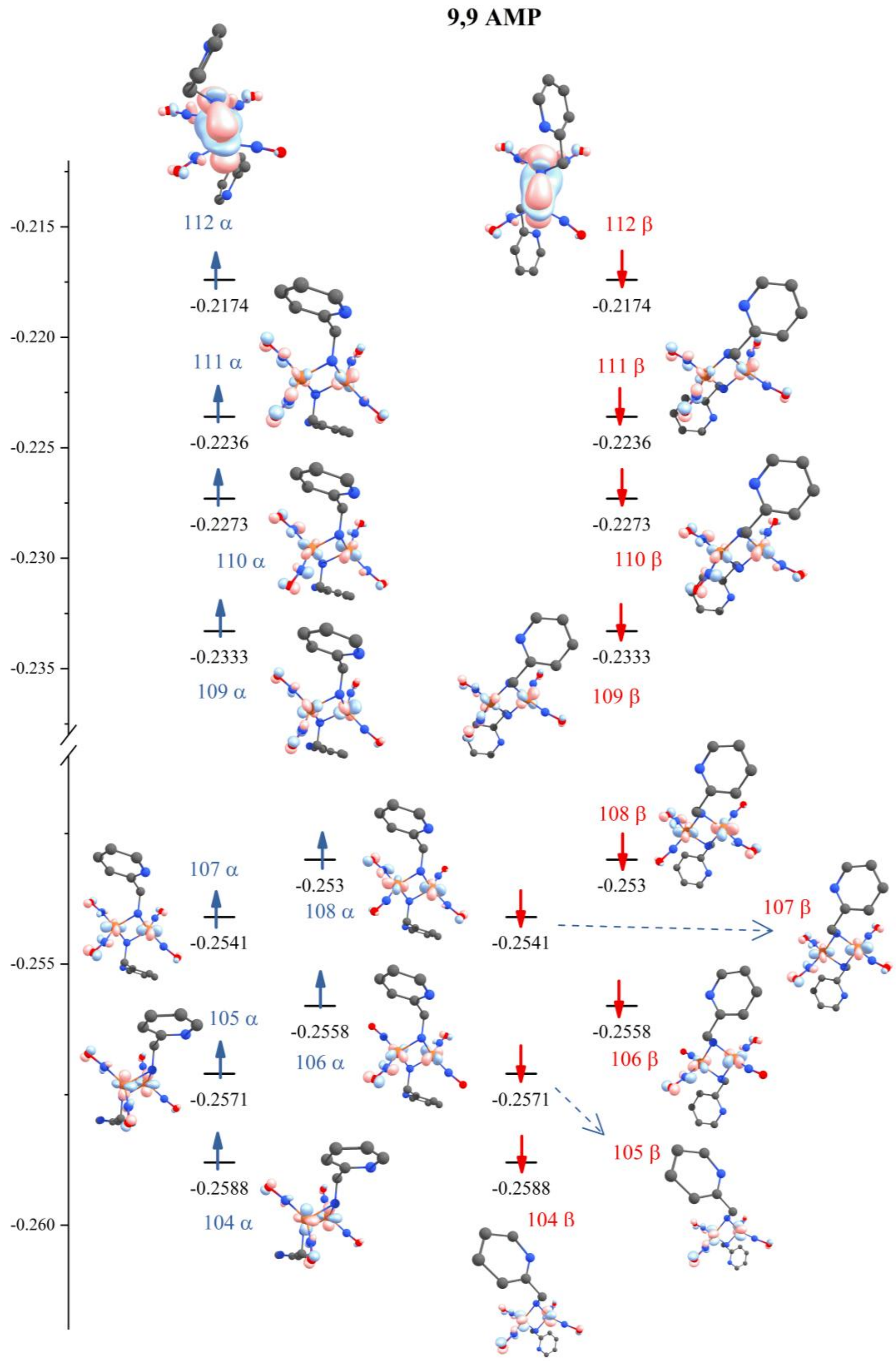

Figure S17. The corresponding orbitals of **9-9 AMP RRE**. The unit of orbital energy is Hartree.

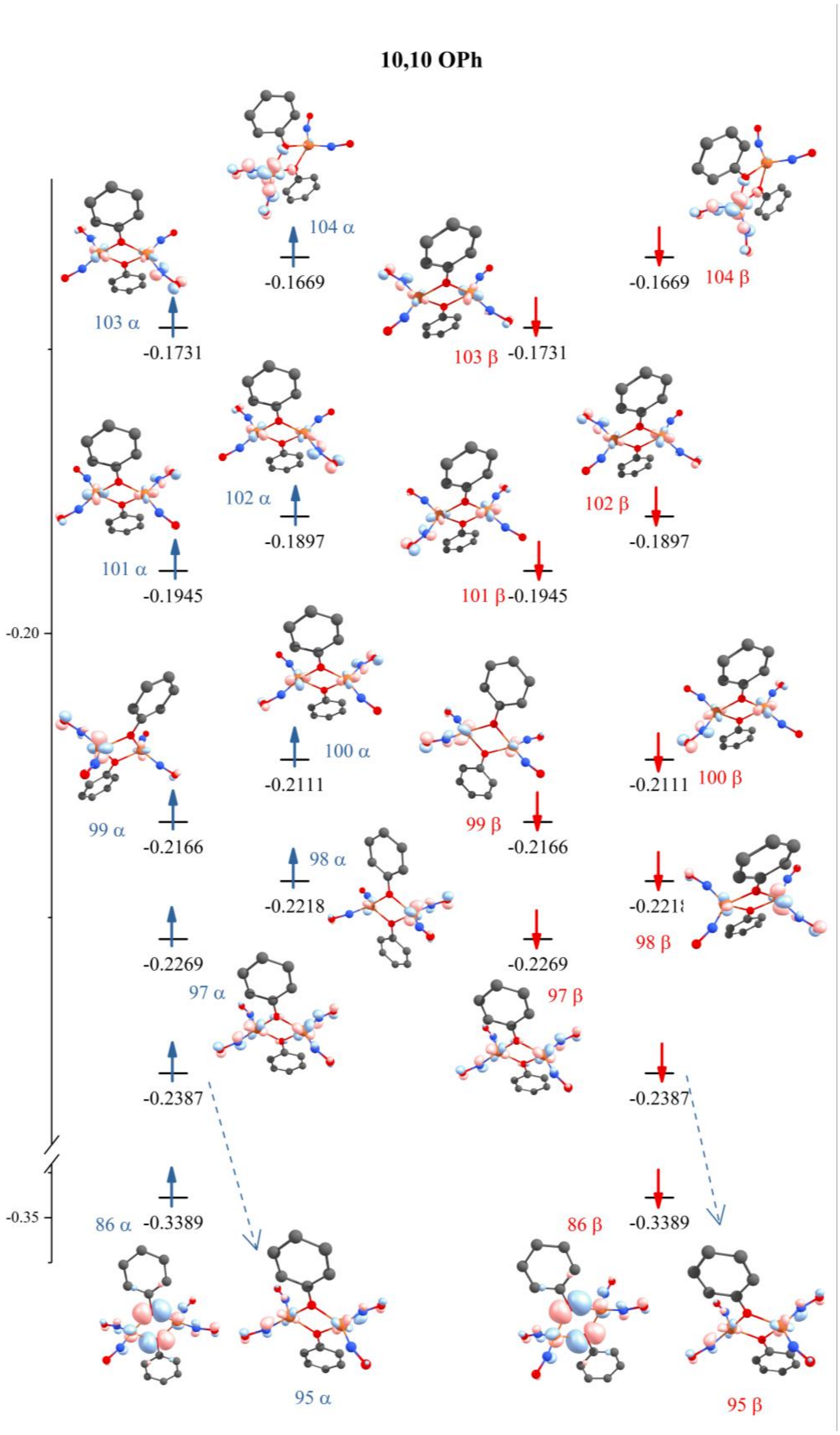

Figure S18. The corresponding orbitals of **10-10 OPh RRE**. The unit of orbital energy is Hartree.

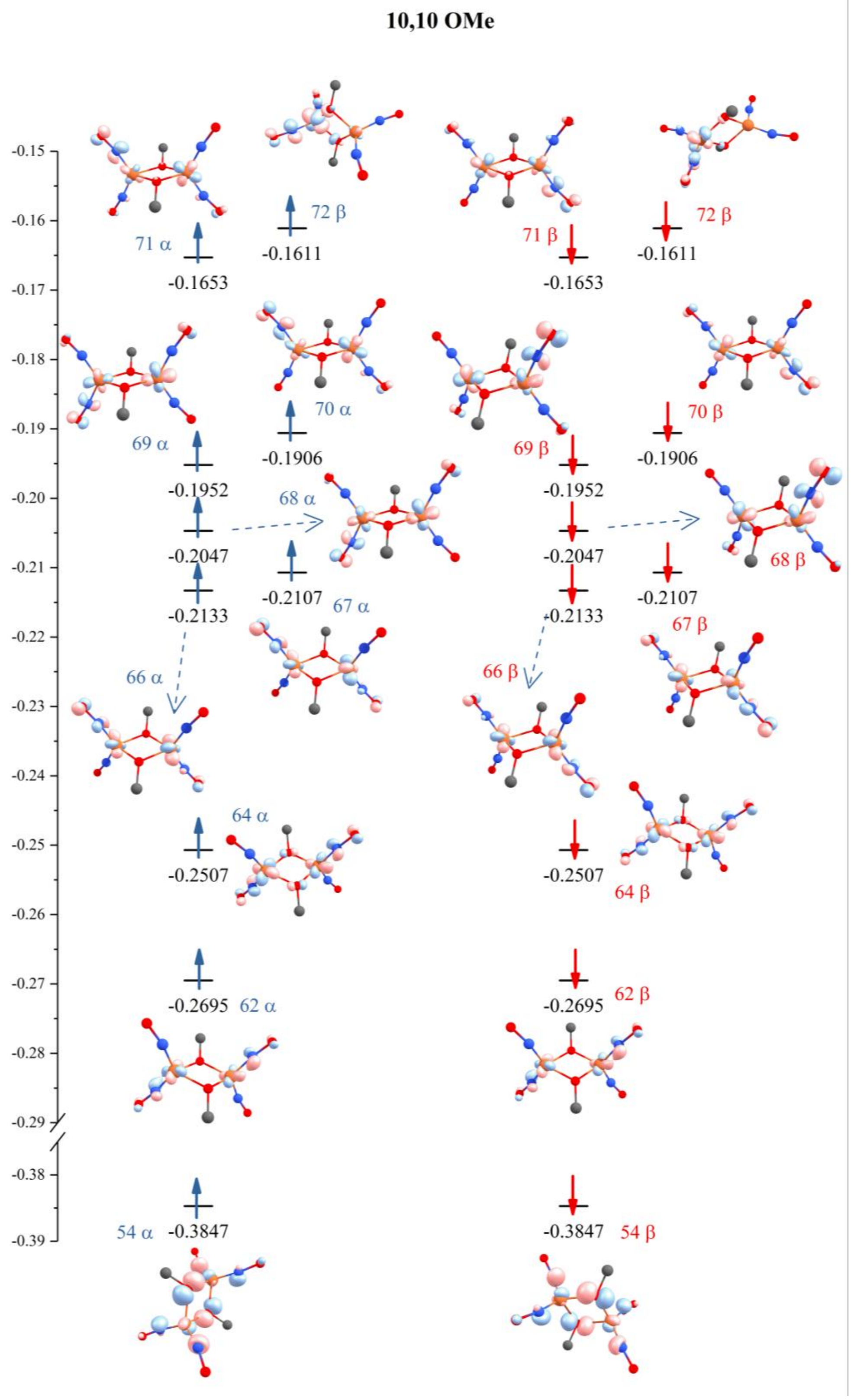

Figure S19. The corresponding orbitals of **10-10 OMe RRE**. The unit of orbital energy is Hartree.

(a)

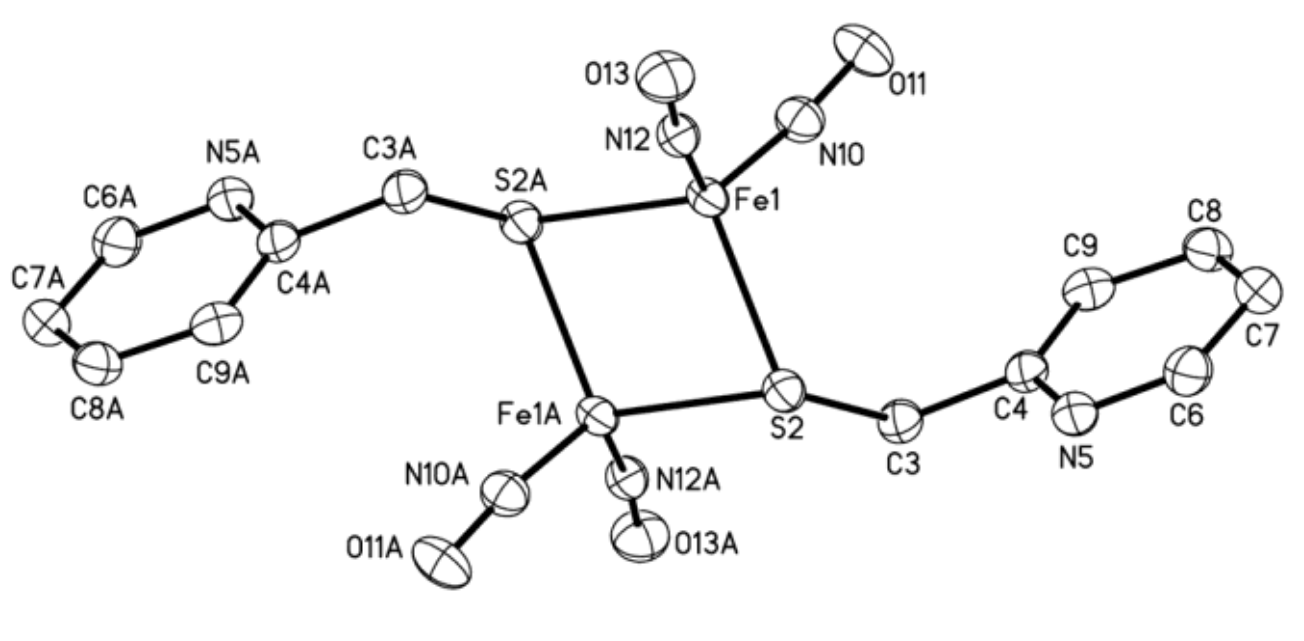

(b)

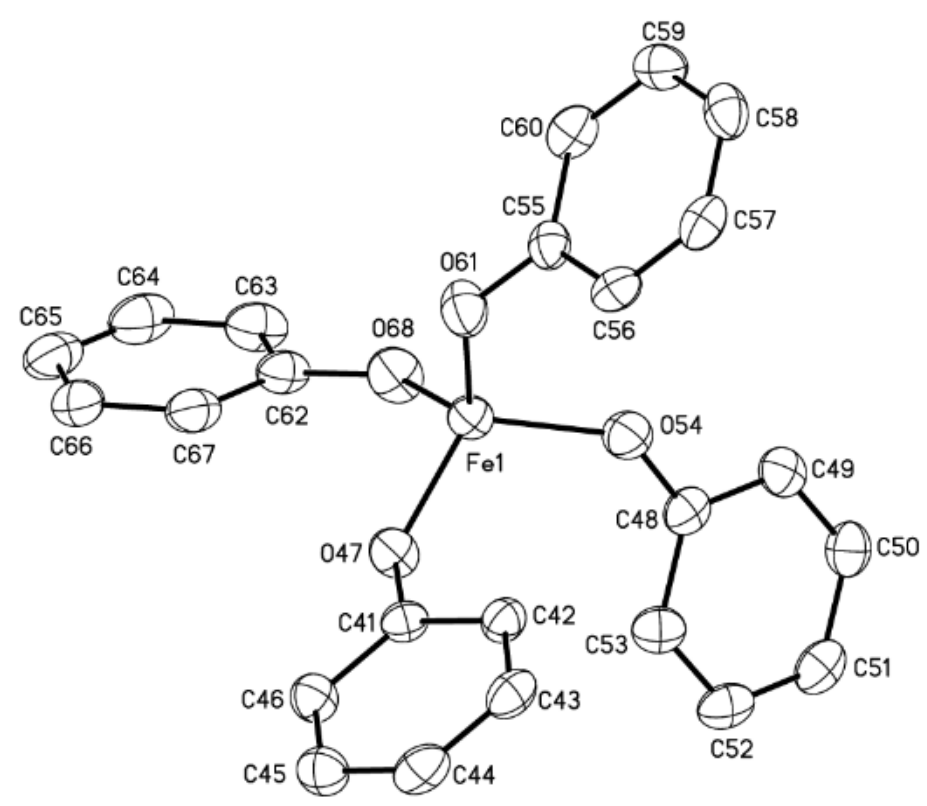

(c)

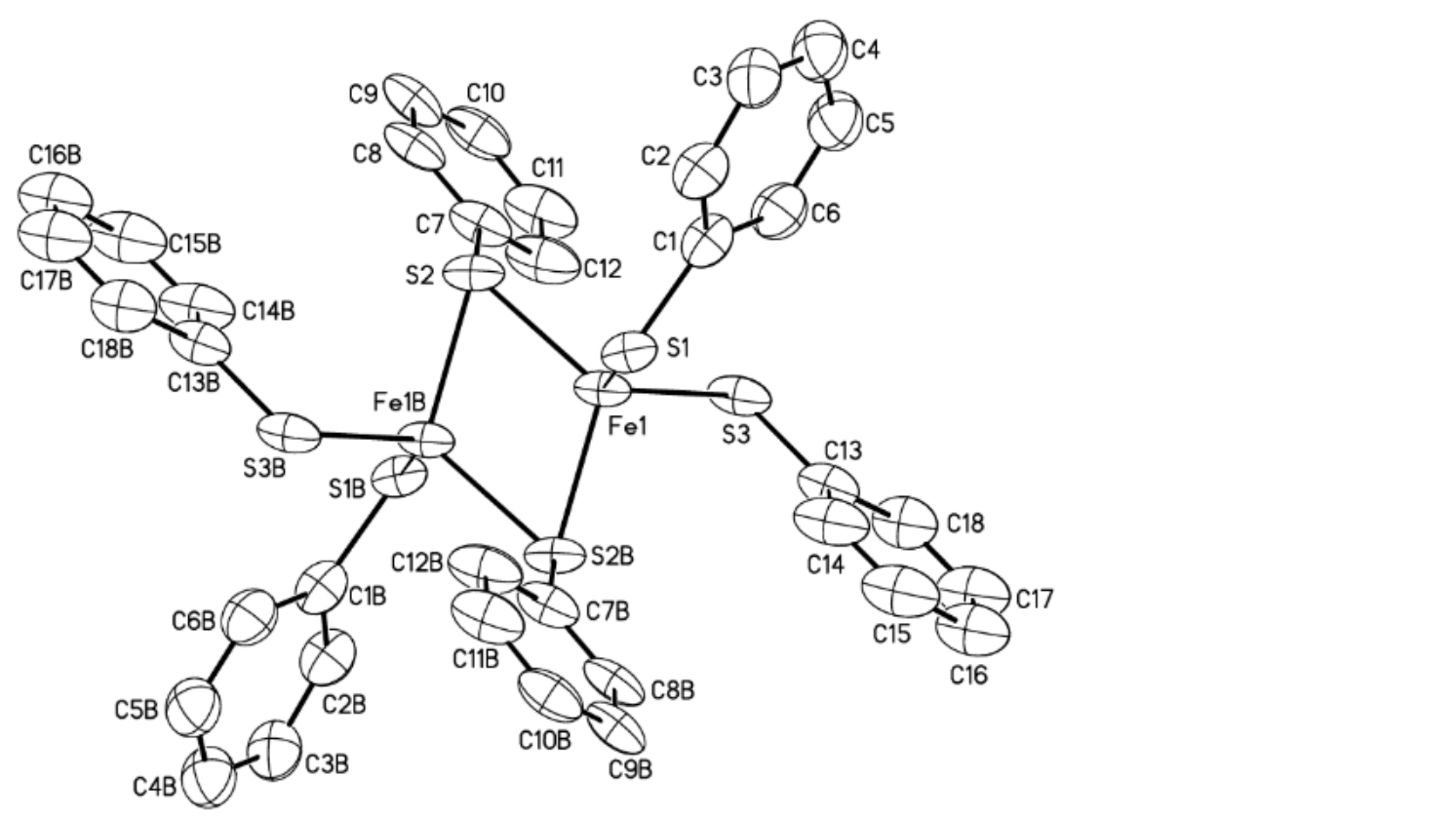

Figure S20. ORTEP drawings and labeling schemes of (a) **9-9 PMT RRE** with thermal ellipsoids drawn at 50% probability. Selected bond distances (Å) and angles (deg): Fe(1)-N(10) 1.667(3), Fe(1)-N(12) 1.662(3), Fe(1)-S(2) 2.2547(9), N(10)-O(11) 1.179(4), N(12)-O(13) 1.179(3), Fe(1)⋯Fe(1A) 2.7075(9); N(10)-Fe(1)-N(12) 118.73(1), S(2)-Fe(1)-S(2A) 106.21(3), Fe(1)-N(10)-O(11) 170.4(3), Fe(1)-N(12)-O(13) 171.0(3). (b) **[PPN][Fe(OPh)$_4$]** with thermal ellipsoids drawn at 50% probability. Selected bond distances (Å) and angles (deg): Fe(1)-O(47) 1.861(2); Fe(1)-O(54) 1.847(2); Fe(1)-O(68) 1.869(2); O(47)-Fe(1)-O(68) 103.44(9); O(54)-Fe(1)-O(47) 114.67(10); O(54)-Fe(1)-O(61) 104.31(10). (c) **[PPN]$_2$[(μ-SPh)Fe(SPh)$_2$]$_2$** with thermal ellipsoids drawn at 50% probability. Selected bond distances (Å) and angles (deg): Fe(1)-S(1) 2.315(4); Fe(1)-S(2) 2.364(4); Fe(1)-S(3) 2.315(4); Fe(1)-S(2B) 2.289(4); S(1)-Fe(1)-S(2) 102.72(15); S(2B)-Fe(1)-S(1) 102.05(16); S(3)-Fe(1)-S(1) 119.59(16); S(3)-Fe(1)-S(2) 115.35(15).

Supplementary Note

Since Fe K-edge VtC $K\beta_{2,5}$ XES spectrum stems from transition from the filled NO valence 2s/2p orbitals to the Fe 1s core hole, the examination of O $K\alpha$ ligand emission, similar to photoemission spectroscopy, is critical because it provides a measure of NO electronic structure that can be correlated to Fe K-edge VtC $K\beta_{2,5}$ XES spectrum. In accordance with the reported O $K\alpha$ XES spectra and the corresponding DFT computation (Figure S21), the energy difference of the calculated σ_{2s}* and σ_{2p} orbital of NO radical, 5.0 eV, is identical to that of reported binding energies of NO derived from X-ray photoelectron spectroscopy (*Surf. Sci.* **1994**, *317*, 397-406; *Angew. Chem. Int. Ed.* **2014**, *53*, 11562-11566). The interesting contribution to Fe K-edge VtC $K\beta_{2,5}$ XES spectrum is seen when it is overlaid with the calculated O $K\alpha$ spectrum; the dominant contribution to the $K\beta_{2,5}$ region come from NO σ_{2s}* and σ_{2p} orbitals. Because the filled π orbitals of end-on NO^{-}/$^{\bullet}NO$/NO^{+} ligand are localized between the nitrogen and oxygen, it is presumed that there is no significant contribution from NO π system owing to the poor overlay with the Fe center. When the energy levels of these MOs are aligned with NO σ_{2p} orbital, addition of electron(s) into the NO π* orbitals attenuates the energy separation between the bonding and antibonding orbitals derived from the σ- and π-bonding interaction between nitrogen and oxygen atoms. This attenuation, meanwhile, increases the energy separation between σ_{2s}* and σ_{2p} orbitals of NO and thus validates the utilization of the linear relationship between $\Delta E_{\sigma 2s^*-\sigma 2p}$ of NO and its oxidation state (Figures 3b and S21b). Further, the experimental Fe K-edge VtC $K\beta_{2,5}$ XES spectrum and spectral deconvolution of **9-9 SPh RRE** are presented in Figure 4. The DFT-computed Fe K-edge VtC $K\beta_{2,5}$ XES spectrum resembles the experimental features, which rationalizes that, at the lower-energy region, the two distinctive VtC transition peaks at ~ 7097.6 and ~ 7102.5 eV are derived from the dominant contribution from end-on NO σ_{2s}* and σ_{2p} orbitals.

(a) 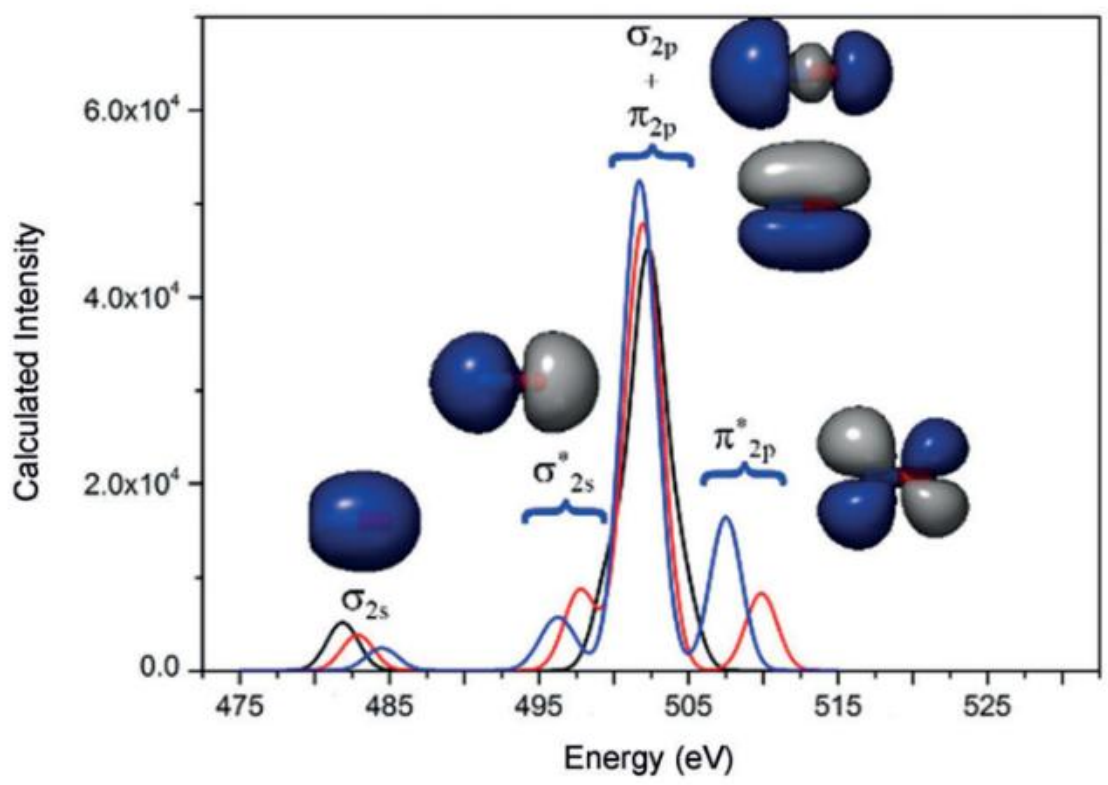

(b) 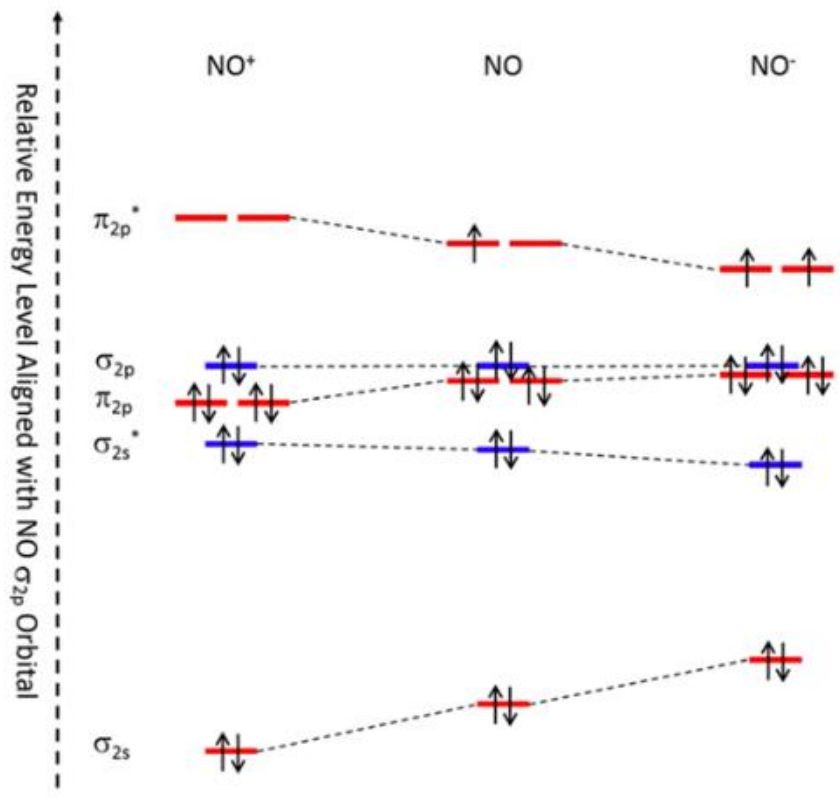

Figure S21. (a) The DFT-calculated O Kα XES spectra of NO^+ (black), $^{\bullet}NO$ (red), and NO^- (blue). (b) The qualitative molecular orbital diagrams present the corresponding molecular orbitals for each of the transitions (*Angew. Chem. Int. Ed.* **2014**, *53*, 11562-11566).

Table S1. A comparison table of the selected bond distances with and without geometry optimization in CRYSTAL23 program.

Bond distances									
OPT structure		r2SCAN							
		99amp	99nhph	99pmt	99sph	910sph	1010ome	1010oph	1010sph
Fe····Fe	Origin	2.536	2.559	2.703	2.693	2.848	3.160	3.26	3.613
	OPT	2.454	2.466	2.652	2.648	2.882	3.118	3.231	3.607
	Diff	-0.082	-0.093	-0.051	-0.045	0.034	-0.042	-0.029	-0.006
Fe-N (NO)	Origin	1.669	1.674	1.671	1.677	1.665	1.651	1.654	1.650
	OPT	1.638	1.638	1.637	1.636	1.664	1.623	1.621	1.625
	Diff	-0.031	-0.036	-0.034	-0.041	-0.001	-0.028	-0.033	-0.025
Fe-L(N/S/O)	Origin	1.966	1.990	2.253	2.262	2.311	2.003	2.029	2.365
	OPT	1.927	1.949	2.254	2.258	2.352	1.980	2.007	2.380
	Diff	-0.039	-0.041	0.001	-0.004	0.041	-0.023	-0.022	0.015
N-O	Origin	1.181	1.174	1.174	1.159	1.184	1.223	1.209	1.207
	OPT	1.207	1.209	1.201	1.199	1.217	1.247	1.239	1.235
	Diff	0.026	0.035	0.027	0.04	0.033	0.024	0.030	0.028

Table S2. Fe (3d) and NO (π*) contributions (spin up, in %) of selected corresponding orbitals.

		Spin up					
**Sample**	**MO**	**Fe (3d)**	**NO (π*)**	**Sample**	**MO**	**Fe (3d)**	**NO (π*)**
**9-9 SPh**	104	67.8	25.7	**9-9 PMT**	112	67.6	25.6
	105	65.4	24		113	64.7	24.2
	106	52.5	27		114	53.3	28
	107	55.4	32.7		115	54.7	31.3
	108	54.9	34.5		116	53.3	35.1
	109	62.5	29.6		117	66.3	25.6
	110	51.7	36.3		118	49	37.9
	111	38.3	44.2		119	37.4	44.4
	112	80.1	3.5		120	81	2.4
**10-10 SPh**	103	44.8	32.3	**9-10 SPh**	104	91.3	1.9
	104	56.8	32		105	77	14.5
	106	60.1	32.4		106	76.6	13.7
	107	60.3	32		107	69.1	15.5
	108	50.6	37.2		108	71.9	17.9
	109	54.5	35.1		109	66.1	19.9
	110	49.3	36.6		110	68.2	18.9
	111	46.2	35.6		111	65	21.2
	112	85.4	2		112	76.7	13.7
	113	51.9	31.5		113	70.2	1.8
**9-9 NHPh**	96	67.2	26	**9-9 AMP**	104	67.4	26.2
	97	66	25.8		105	65.3	25.4
	98	55.3	26.8		106	55.8	27.7
	99	55.8	33.4		107	52.3	35.4
	100	54.4	33		108	54.2	33
	101	52.4	36.3		109	56.7	33.7
	102	52.5	36.3		110	47.7	40
	103	35.7	47.1		111	34.8	48.9
	104	80.4	4.7		112	82.6	4.1
**10-10 OMe**	54	6.7	19.3	**10-10 OPh**	86	3.1	10.8
	62	43.5	43.4		95	36.4	50.1
	64	51.7	32.2		97	47	39.9
	66	53.6	37.1		98	59	34
	67	52.2	38.7		99	64.6	27.6
	68	49.4	37.5		100	51.2	36.9
	69	53.9	35.7		101	53.4	35.9
	70	48.4	38.9		102	49.4	38.1
	71	43.1	40.6		103	43.5	39.8
	72	63.4	23.7		104	62.1	26

Table S3. Fe (3d) and NO (π*) contributions (spin down, in %) of selected corresponding orbitals.

Spin down							
**Sample**	**MO**	**Fe (3d)**	**NO (π*)**	**Sample**	**MO**	**Fe (3d)**	**NO (π*)**
**9-9 SPh**	104	67.8	25.8	**9-9 PMT**	112	67.7	25.6
	105	65.5	24		113	64.7	24.2
	106	52.5	27		114	53.3	28
	107	55.4	32.9		115	54.7	31.3
	108	55.2	34.3		116	53.2	35.1
	109	62.5	29.6		117	66.3	25.6
	110	51.6	36.6		118	49	37.9
	111	38.2	44.1		119	37.5	44.6
	112	80.1	3.5		120	80.9	2.3
**10-10 SPh**	103	44.8	32.3	**9-10 SPh**	104	87.5	2.4
	104	56.8	32		105	45	45.6
	106	60	32.4		106	44	45.9
	107	60.2	32.1		107	38.5	46.5
	108	50.6	37.2		108	37.7	51.1
	109	54.4	35.1		109	33.1	52.8
	110	49.5	36.6		110	30.2	58
	111	46.2	35.6		111	23.2	64.8
	112	85.4	2		112	32	58.3
	113	51.9	31.5				
**9-9 NHPh**	96	67.2	25.9	**9-9 AMP**	104	67.4	26.2
	97	66	25.8		105	65.3	25.4
	98	55.3	26.8		106	55.8	27.7
	99	55.8	33.4		107	52.4	35.4
	100	54.5	33		108	54.2	32.9
	101	52.4	36.3		109	56.7	33.7
	102	52.5	36.3		110	47.7	40
	103	35.7	47.1		111	34.8	48.9
	104	80.4	4.7		112	82.6	4.1
**10-10 OMe**	54	6.7	19.3	**10-10 OPh**	86	3.1	10.8
	62	43.5	43.4		95	36.4	50.1
	64	51.7	32.2		97	47	39.9
	66	53.5	37.1		98	66.3	27.3
	67	52.3	38.7		99	57.4	34.3
	68	49.5	37.5		100	51.2	36.8
	69	53.9	35.7		101	53.5	35.7
	70	48.4	38.9		102	49.4	38.1
	71	43.1	40.6		103	43.5	39.9
	72	63.3	23.7		104	62	26

Table S4. Statistics values of Fe (d) and NO (π*) contributions in the corresponding orbitals.

RREs	Fe (d)			NO (π*)		
	spin up	spin down	sum	spin up	spin down	sum
**9-9 SPh**	5.286	5.288	10.574	2.575	2.578	5.153
**9-10 SPh**	7.321	3.712	11.033	1.39	4.254	5.644
**10-10 SPh**	5.599	5.598	11.197	3.067	3.068	6.135
**9-9 PMT**	5.273	5.273	10.546	2.545	2.546	5.091
**9-9 NHPh**	5.197	5.198	10.395	2.694	2.693	5.387
**9-9 AMP**	5.168	5.169	10.337	2.744	2.743	5.487
**10-10 OPh**	4.666	4.667	9.333	3.283	3.281	6.564
**10-10 OMe**	4.659	4.659	9.318	3.471	3.471	6.942

Table S5. Crystallographic data for Complex **9-9 PMT RRE**.

Empirical formula	$C_{12}H_{12}Fe_2N_6O_4S_2$
Formula weight	480.10
Temperature/K	100.00(10)
Crystal system	triclinic
Space group	P-1
a/Å	6.4864(4)
b/Å	8.3299(4)
c/Å	8.6108(5)
α/°	75.597(5)
β/°	86.515(5)
γ/°	77.184(5)
Volume/Å^3	439.39(4)
Z	1
ρ_{calc}g/cm^3	1.814
μ/mm^{-1}	15.767
F(000)	242.0
Crystal size/mm^3	0.13 × 0.02 × 0.02
Radiation	Cu Kα (λ = 1.54184)
2θ range for data collection/°	10.608 to 146.21
Index ranges	$-7 \le h \le 8$, $-10 \le k \le 7$, $-10 \le l \le 10$
Reflections collected	4677
Independent reflections	1659 [R_{int} = 0.0408, R_{sigma} = 0.0461]
Data/restraints/parameters	1659/0/118
Goodness-of-fit on F^2	1.090
Final R indexes [I>=2σ (I)]	R_1 = 0.0361, wR_2 = 0.0885
Final R indexes [all data]	R_1 = 0.0443, wR_2 = 0.0918
Largest diff. peak/hole / e Å^{-3}	0.38/-0.35

Table S6. Crystallographic data for Complex **[PPN][Fe(OPh)$_4$]**.

Empirical formula	$C_{60}H_{50}FeNO_4P_2$
Formula weight	966.80
Temperature/K	100.00(14)
Crystal system	triclinic
Space group	P-1
a/Å	10.4267(3)
b/Å	13.4128(4)
c/Å	18.6323(6)
α/°	81.741(3)
β/°	76.560(3)
γ/°	72.606(3)
Volume/Å^3	2410.76(14)
Z	2
ρ_{calc}g/cm^3	1.332
μ/mm^{-1}	3.532
F(000)	1010.0
Crystal size/mm^3	0.15 × 0.14 × 0.05
Radiation	Cu Kα (λ = 1.54184)
2θ range for data collection/°	6.928 to 153.808
Index ranges	$-11 \leq h \leq 13$, $-16 \leq k \leq 16$, $-23 \leq l \leq 23$
Reflections collected	29378
Independent reflections	9501 [R_{int} = 0.0474, R_{sigma} = 0.0458]
Data/restraints/parameters	9501/0/613
Goodness-of-fit on F^2	1.041
Final R indexes [I>=2σ (I)]	R_1 = 0.0597, wR_2 = 0.1657
Final R indexes [all data]	R_1 = 0.0654, wR_2 = 0.1707
Largest diff. peak/hole / e Å^{-3}	0.84/-1.16

Table S7. Crystallographic data for Complex **$[PPN]_2[(\mu\text{-}SPh)Fe(SPh)_2]_2$**.

Empirical formula	$C_{54}H_{45}FeNP_2S_3$
Formula weight	921.88
Temperature/K	99.9(2)
Crystal system	triclinic
Space group	P-1
a/Å	14.4171(2)
b/Å	14.6535(2)
c/Å	15.0444(2)
α/°	69.3590(10)
β/°	62.0250(10)
γ/°	72.7990(10)
Volume/Å^3	2593.85(7)
Z	2
ρ_{calc}g/cm^3	1.180
μ/mm^{-1}	4.297
F(000)	960.0
Crystal size/mm^3	0.11 × 0.07 × 0.02
Radiation	Cu Kα (λ = 1.54184)
2θ range for data collection/°	6.878 to 134.156
Index ranges	$-11 \leq h \leq 16, -17 \leq k \leq 17, -17 \leq l \leq 17$
Reflections collected	31441
Independent reflections	9195 [R_{int} = 0.0214, R_{sigma} = 0.0176]
Data/restraints/parameters	9195/2139/1100
Goodness-of-fit on F^2	1.033
Final R indexes [I>=2σ (I)]	R_1 = 0.0841, wR_2 = 0.2184
Final R indexes [all data]	R_1 = 0.0899, wR_2 = 0.2229
Largest diff. peak/hole / e Å^{-3}	0.53/-0.59
